# Supplementary material for: A green solvent enables precursor phase engineering of stable formamidinium lead triiodide perovskite solar cells
Source: Nat Commun. 2024 Nov 22;15:10110. doi: 10.1038/s41467-024-54113-4 (PMC11582696; doi:10.1038/s41467-024-54113-4)
Supplement: Supplementary file 1 — Supplementary Information [file 41467_2024_54113_MOESM1_ESM.pdf]

Supplementary Information

A green solvent enables precursor phase engineering of stable formamidinium lead triiodide perovskite solar cells

Benjamin M. Gallant[1,2], Philippe Holzhey[1], Joel A. Smith[1], Saqlain Choudhary[1], Karim A. Elmestekawy[1], Pietro Caprioglio[1], Igal Levine[3,4], Alexandra A. Sheader[1], Esther Y-H. Hung[1], Fengning Yang[1], Daniel T.W. Toolan[5,6], Rachel C. Kilbride[6], Karl-Augustin Zaininger[1], James M. Ball[1], M. Greyson Christoforo[1], Nakita K. Noel[1], Laura M. Herz[1,7], Dominik J. Kubicki[2], Henry J. Snaith[1]*

[1] Clarendon Laboratory, Department of Physics, University of Oxford, Parks Road, Oxford, OX1 3PU, United Kingdom
[2] School of Chemistry, University of Birmingham, B15 2TT, Birmingham, UK
[3] Solar Energy Division, Helmholtz-Zentrum Berlin für Materialien und Energie GmbH, Berlin, 12489, Germany
[4] Institute of Chemistry and The Center for Nanoscience and Nanotechnology, The Hebrew University, Jerusalem 91904, Israel
[5] Department of Materials, University of Manchester, Manchester, M13 9PL, UK
[6] Department of Chemistry, University of Sheffield, Sheffield, S3 7HF, UK
[7] Institute for Advanced Study, TU Munich, Lichtenbergstr. 2a, 85748 Garching, Germany

Corresponding Author: Henry J. Snaith (henry.snaith@physics.ox.ac.uk)

Supplementary Methods

Materials

Fluorine-doped tin oxide-coated glass substrates (8 or 15 Ω cm^{-2}, AMG), tin (IV) oxide (15 wt. % in H_2O colloidal dispersion, Alfa Aesar), MeO-2PACz ([2-(3,6-Dimethoxy-9H-carbazol-9-yl)ethyl]phosphonic acid, >98.0%, Tokyo Chemical Industries), lead(II) iodide (99.99 %, trace metal basis, Tokyo Chemical Industries), methylammonium iodide (>99.99 %, Greatcell Solar Materials), formamidinium iodide (>99.99 %, Greatcell Solar Materials), phenethylammonium iodide (>99 %, Greatcell Solar Materials), ethylenediammonium diiodide (Sigma Aldrich), $PC_{60}BM$ ([6,6]-Phenyl-C61-butyric acid methyl ester), bathocuproine (BCP, >95.0%, Tokyo Chemical Industries), spiro-OMeTAD (2,2',7,7'-Tetrakis(N,N-di-p-methoxyphenylamino)-9,9'-spirobifluoren, >99.5 %, Luminescence Technology Corp.), FK209 Co(III) TFSI salt (tris(2-(1H-pyrazol-1-yl)-4-tert-butylpyridine)cobalt(III) tri[bis(trifluoromethane)sulfonimide], 98 %, Sigma Aldrich), bis(trifluoromethylsulfonyl)amine lithium salt (99.95 %, Sigma Aldrich), gold pellets (99.999 %, Kurt J. Lesker Company).

Ethanol (200 proof, anhydrous, >99.5%, Sigma Aldrich), *n*-butylamine (99.5 %, Sigma Aldrich), 2-methyltetrahydrofuran (Biorenewable, anhydrous, >99.0 %, contains 250 ppm BHT as inhibitor, Sigma Aldrich), tetrahydrofuran (anhydrous, >99.9 %, contains 250 ppm BHT as inhibitor, Sigma Aldrich), *n*-butanol (anhydrous, 99.8 %, Sigma Aldrich), aluminium oxide nanoparticles (20 wt.% in 2-propanol, Sigma Aldrich), 2-propanol (anhydrous, 99.5 %, Sigma Aldrich), chlorobenzene (anhydrous, 99.8 %, Sigma Aldrich), acetonitrile (anhydrous, 99.8 %, Sigma Aldrich), 4-*tert*-butylpyridine (98 %, Sigma Aldrich), toluene (anhydrous, 99.8%, Sigma Aldrich), chlorobenzene (anhydrous, 99.8%, Sigma Aldrich), 1,2-dichlorobenzene (anhydrous, 99%, Sigma Aldrich).

Prior to use, all FTO-coated substrates were scrubbed with an aqueous 2 vol % Decon 90 solution, rinsed with deionised water, sonicated in acetone, and subsequently sonicated in 2-propanol.

Besides aluminium oxide nanoparticles, all other non-aqueous chemicals were stored in a N_2-filled glovebox before use and protected from exposure to light.

Fabrication

n-i-p configuration

A colloidal suspension of tin oxide nanoparticles (400 μL) was diluted with ultrapure water (2,600 μL). In an atmosphere containing minimum moisture (<5 % relative humidity), 200 μL of this solution was placed statically on a UV-ozone treated (15 minutes) substrate coated with a fluorine-doped tin oxide layer (15 Ω cm^{-2}), then spun at 4,000 rpm (1,000 rpm s^{-1}) for 30 seconds before being immediately annealed at 150 °C for 30 minutes in the same environment. The substrates were allowed to cool, then immediately subjected to a further 15 minutes of UV-ozone treatment before being immediately used in the following processing step.

2-methyltetrahydrofuran (1,500 μL) was added to lead iodide (1.266 mmol, 583.5 mg) and methylammonium iodide (1.139 mmol, 181.1 mg), followed by *n*-butylamine (1.899 mmol, 187.6 μL), and agitated until all solids were fully dissolved. This corresponds to an $R_{BA\text{-}MA+}$ value of 1.67. 50 μL of this solution was spin-coated dynamically on top of the SnO_2 layer at 2,500 rpm for 45 seconds in an environment at between 20-22 % relative humidity and <22 °C. The substrates were immediately annealed at 70 °C for 10 minutes in the same environment. After cooling, the substrates were coated with 350 μL of a 0.1 M solution of formamidinium iodide (0.500 mmol, 86.0 mg) dissolved in *n*-butanol (5,000 μL). After 45 seconds of static soaking, the substrates were spun at 4,000 rpm (1,000 rpm s^{-1}) for 45 seconds, then immediately annealed for 10 minutes at 70 °C, followed by 30 minutes at 180 °C. We summarise this process schematically in **Supplementary Fig. 1**.

(Note: The full fabrication procedures for all other perovskite compositions utilised in this work are described in **Supplementary Note 6**).

The substrates were allowed to cool, then a 20 mM solution of phenethylammonium iodide (0.200 mmol, 49.8 mg) in 2-propanol (10,000 μL) was spin-coated dynamically on top at 5,000 rpm for 45 seconds, in a N_2-containing glovebox.

A solution of spiro-OMeTAD (0.070 mmol, 85.8 mg) dissolved in chlorobenzene (1,000 µL) and doped with 8.4 µL of a 0.250 M solution of tris(2-(1*H*-pyrazol-1-yl)-4-*tert*-butylpyridine)cobalt(III) tri[bis(trifluoromethane)sulfonimide] in acetonitrile, 19.4 µL of a 1.800 M solution of bis(trifluoromethylsulfonyl)amine lithium salt in acetonitrile, and *tert*-butyl pyridine (0.231 mmol, 38.0 µL). This solution was spin-coated on the PEAI-passivated substrates dynamically at 2,500 rpm for 30 seconds, in a N_2-containing glovebox.

Finally, 75 nm of gold was evaporated on top of the substrates at an initial rate of 0.1 A s^{-1} (to 5 nm, then ramped gradually to 1.0 A s^{-1}) at a pressure < 2 x 10^{-6} torr.

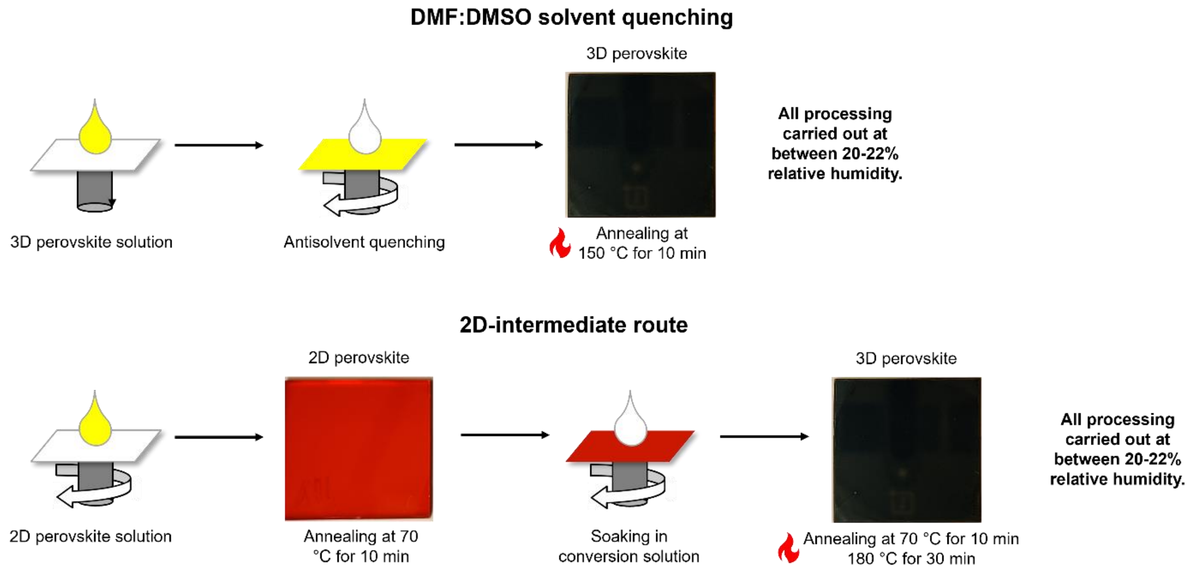

Supplementary Figure 1 | Processing schematic. Schematic highlighting differences between conventional antisolvent-quenched processing of $FAPbI_3$ from DMF:DMSO precursor inks and the 2D-intermediate processing route for $FAPbI_3$ developed in this work.

p-i-n configuration

In a N_2 glovebox, a filtered (0.22 µm PTFE syringe filter) 0.33 mg mL^{-1} solution of MeO-2PACz ([2-(3,6-Dimethoxy-9H-carbazol-9-yl)ethyl]phosphonic acid) in anhydrous ethanol (350 µL) was prepared and placed statically on a UV-ozone treated (30 minutes) substrate coated with a fluorine-doped tin oxide layer (15 Ω cm^{-2}), allowed to spread for 10 seconds, then spun at 3,000 rpm (600 rpm s^{-1}) for 30 seconds before being immediately annealed at 100 °C for 10 minutes in the same environment.

Separately, a colloidal suspension of alumina nanoparticles (50 µL) was diluted with 2-propanol (7,500 µL) and ultrasonicated for at least 30 minutes ("1:150 Al_2O_3 NP:IPA"). This solution was filtered immediately before use (0.22 µm PTFE syringe filter). Preparation of this solution was carried out in ambient air, but all spin-coating was carried out in a N_2 glovebox.

The MeO-2PACz-coated substrates were allowed to cool, then in the same environment the solution of alumina nanoparticles (80 µL) was dispensed dynamically whilst the substrate was spun at 5,000 rpm (5,000 rpm s^{-1}) for 20 seconds. The substrate was then annealed at 100 °C for 5 minutes in the same environment. We have included this layer in the p-i-n device architecture in response to the poor wettability of perovskite solutions on carbazole-based SAMs, as has been reported elsewhere[1]. The use of Al_2O_3 nanoparticles in this way forms a thin mesoporous layer on top of the SAM, improving wettability[2].

2-methyltetrahydrofuran (1,500 µL) and *n*-butylamine (1.688 mmol, 166.8 µL) were added to lead iodide (1.125 mmol, 518.6 mg) and methylammonium iodide (1.013 mmol, 161.0 mg) and agitated until all solids were fully dissolved. This corresponds to an $R_{BA\text{-}MA+}$ value of 1.67. 50 µL of this solution was spin-coated dynamically on top of the SnO_2 layer at 2,500 rpm for 40 seconds in an environment at between 20-22 % relative humidity and <22 °C. The substrates were immediately annealed at 70 °C for 10 minutes in the same environment. After cooling, the substrates were coated with 350 µL of a 0.1 M solution of formamidinium iodide (0.500 mmol, 86.0 mg) dissolved in *n*-butanol (5,000 µL). After 45 seconds of static soaking, the substrates were spun at 4,000 rpm (1,000 rpm s^{-1}) for 45 seconds, then immediately annealed for 10 minutes at 70 °C, followed by 30 minutes at 180-185 °C.

In ambient air, a colloidal suspension of alumina nanoparticles (50 µL) was diluted with 2-propanol (5,000 µL) and ultrasonicated for at least 30 minutes ("1:100 Al_2O_3 NP:IPA")[2]. In a N_2 glovebox, a 0.5 mg mL^{-1} solution of ethylenediammonium diiodide ($EDAI_2$) in a 1:1 mixture of anhydrous 2-propanol and toluene was prepared and heated for between 2 and 4 hours[3]. Also, a 30 mg mL^{-1} solution of $PC_{60}BM$ ([6,6]-Phenyl-C61-butyric acid methyl ester) in a 3:1 mixture of chlorobenzene:dichlorobenzene, and a 0.5 mg mL^{-1} solution of bathocuproine

(BCP) in anhydrous 2-propanol. All these solutions were filtered immediately before use (0.22 µm PTFE syringe filter).

In a N_2 glovebox, the solution of $EDAI_2$ (325 µL) was deposited on the on the previously deposited perovskite layer, and the substrate immediately spun at 3,000 rpm (1,333 rpm s^{-1}) to remove excess solution. The substrate was immediately annealed for 10 minutes at 100 °C in the same environment. $EDAI_2$ surface treatment has previously been reported as a highly effective strategy of improving both the V_{oc} and operational stability of PSCs[4].

The substrate was allowed to cool, then the 1:150 Al_2O_3 NP:IPA solution (80 µL) was spin coated dynamically at 5,000 rpm (5,000 rpm s^{-1}), before being further annealed at 100 °C for 5 minutes in the same environment. Analogously to our use of Al_2O_3 nanoparticles to form a mesoporous underlayer beneath the perovskite, here we use the same strategy to combat the roughness of the perovskite surface. This roughness is associated with the large (>1,500 nm) grains often observed in our $FAPbI_3$ material and leads to very poor adhesion and atomic-level contact between the PCBM electron transport layer and the perovskite. Recently in our group we have a developed a strategy by which a mesoporous alumina layer is introduced, and the PCBM solution allowed to intercalate into this structure prior to spin-coating (see below) to improve the contact between the perovskite and the fullerene layer [2]. We have also found that this layer is beneficial for long-term operational stability of the PSC, and as the aim of our p-i-n devices was to achieve the highest possible stability for control and target devices, this strategy was particularly selected for this reason[2].

The substrate was allowed to cool, then the $PC_{60}BM$ solution (200 µL) was deposited on the substrate and allowed to spread for 10 seconds[2], before being spin coated at 2,000 rpm (2,000 rpm s^{-1}). The substrate was then annealed at 100 °C for 5 minutes in the same environment. Finally, once the substrates had cooled, the BCP solution (100 µL) was spin coated dynamically at 5,000 rpm (5,000 rpm s^{-1}), and the substrate annealed at 100 °C for 2 minutes in the same environment.

Using a chromium-coated tungsten bar, 3.5 nm of chromium was deposited on top of the substrate at a rate of 0.2 A s^{-1}, followed immediately by 100 nm of gold, which was evaporated on top of the substrates at an initial rate of 0.1 A s^{-1} (to 5 nm, then ramped gradually to 1.2 A s^{-1}), all at a pressure < 2 x 10^{-6} torr. A layer of chromium was deposited prior to gold as this has previously been reported as reducing Au migration through to the perovskite layer thus increasing the long-term operational stability of the PV devices, which was our aim in fabricating these p-i-n configuration PSCs. We note, however, that use of this Cr layer typically reduces device PCE by ~1%.

Characterisation

Time-integrated Photoluminescence

PL spectra were acquired using a time-correlated single photon counting (TCSPC) setup (FluoTime 300, PicoQuant GmbH). Samples were photoexcited using a 507 nm laser (LDH-P-C-510, Pico Quant GmbH) pulsed at a frequency of 40 MHz. The PL was dispersed using a monochromator, detected with hybrid photomultiplier detector assembly (PMA Hybrid 40, PicoQuant GmbH) and integrated over time.

Ultraviolet-Visible Absorption Spectroscopy

Reflectance and transmittance spectra were recorded on a Varian Cary 1050 UV Vis spectrophotometer equipped with an integrating sphere. From these measurements, in combination with the photoactive layer thickness, absorption coefficients were calculated assuming a direct bandgap semi-conductor. Separately, absorbance spectra were measured with a Varian Cary 300 Bio UV-visible spectrophotometer with a 50x50 mm reflective neutral density filters with an optical density of 3.0 (made out of UV fused silica).

Visible Light Microscopy

Optical microscope images were taken on a Nikon Eclipse LV100ND microscope with Nikon TU Plan Fluor lenses (10x/0.30 A, 20x/0.45 A, 50x/0.60 B, 100x/0.90 A). The images are taken with an attached Nikon Digital Camera D6.10.

Time-Correlated Single Photon Counting (TCSPC)

Time-resolved PL of the thin films on quartz substrates was measured using TCSPC following excitation by a 398 nm picosecond pulsed diode laser at a repetition rate of 2.5 MHz (PicoHarp, LDH-D-C-405M). The resultant PL was collected and coupled into a grating spectrometer (Princeton Instruments, SP-2558), which directed the spectrally dispersed PL onto a photon-counting detector (PDM series from MPD), whose timing was controlled with a PicoHarp300 TCSPC event timer. The PL transients were measured at 810 nm, corresponding to the peak PL intensity in the steady-state PL spectrum of this composition.

The excitation fluence of 81 nJ cm^{-2}, corresponding to a charge-carrier density of the order of 10^{15} cm^{-3}, was used to ensure that the dominant recombination pathway is through monomolecular recombination. To account for both the time dependence and the local distribution of k_1 recombination rates, we fitted a stretched exponential function $I = I_0 e^{(\frac{t}{\tau_{\mathrm{char}}})^{\beta}}$ to the PL intensity to account for the local distribution of monoexponential decay rates, whose effective lifetime is given by $\tau_{\mathrm{eff}} = \frac{\tau_{\mathrm{char}}}{\beta}\Gamma(\frac{1}{\beta})$. τ_{char} is the characteristic lifetime corresponding to the time taken for the PL intensity to drop to I_0/e, and β is the distribution coefficient that encompasses the case for monoexponential decays for the case $\beta = 1$, and the cases for a range of decay times (or potentially higher-order effects) as it decreases[5,6]. To account for the PL originating from electron-hole recombination we further double the effective fitted lifetime in order to extract the monomolecular charge-carrier recombination rate k_1, such that $k_1 = 1/2\tau_{\mathrm{eff}}$, assuming $I \propto k_2 n^2$ at our excitation fluence of 81 nJ cm^{-2} [7,8]. The samples were all mounted in a vacuum cell under low pressure ($\sim 10^{-2}$ mbar). We note that the absence of oxygen prevents the possible passivation of the photo-generated defects in the film and could

have resulted in an underestimation of the lifetimes extractable under ambient operating conditions.

This spectroscopic technique initially generated charge-carriers closer to the surface of the film through which excitation occurs, owing to most of the 398 nm excitation being absorbed in the first 100 nm. This also accounts for the fast decay at very early times; most of the photogenerated charge-carriers will be initially generated closer to the surface where the density of trap states is higher than in the bulk, resulting in a rapid drop in PL intensity at very early times before the charge-carriers diffuse away further from the surface. The diffusion along the thickness of the film towards the bulk and perpendicular diffusion away from the excitation spot results in the local distribution of mono-exponential recombination rates assumed for our use of the stretch exponential fits.

Optical-Pump Terahertz-Probe (OPTP) Spectroscopy

An amplified laser system (Spectra Physics, MaiTai – Ascend – Spitfire), with a 5 kHz repetition rate, centre wavelength of 800 nm and pulse duration of 35 fs is used to generate the THz radiation using a spintronic emitter. The THz probe is then focused onto the sample, overlaid with a 400 nm excitation pump that is generated using a Beta Barium Borate (BBO) crystal. The THz radiation transmitted through the sample is then detected via free-space electro-optical sampling in a ZnTe (110) crystal of thickness 200 μm[9].

X-ray Diffraction (XRD)

The 1D-XRD patterns were obtained with a Panalytical X'Pert Pro X-Ray diffractometer and In-situ 2D-XRD patterns (**Fig. 5a-g**, **Supplementary Fig. 8** and **20**) using a Rigaku SmartLab X-ray diffractometer and a HyPix-3000 2D hybrid pixel array detector, both with $CuK_{\alpha1}$ (1.54060 Å) source. A heating stage was employed in conjunction with the Rigaku SmartLab diffractometer for these measurements.

Grazing-incidence wide-angle X-ray scattering (GIWAXS) data (**Supplementary Fig. 6**) was acquired at the I07 beamline at Diamond Light Source. X-rays with energy 10 keV were

incident on the samples at a grazing incidence angle α_i = 0.5-3°, with scattering collected by a Pilatus 2M (DECTRIS) hybrid photon-counting detector with a sample-to-detector distance (SDD) of 375 mm, with the geometry calibrated using LaB_6. *In-situ* GIWAXS data (**Figure 2b**) was measured using the same configuration, with spin-coated 2D intermediate samples aligned before FAI solutions were deposited on top using an *in-situ* blade coater in ambient conditions. The coating apparatus incorporates a syringe driver, motorised blade, integrated hotplate and an N2 outlet directed at the sample to remove excess solvent after conversion. Solutions were deposited via a microfluidic tube adjacent to the measured area, and coated perpendicular to the beam with the blade set with a shim height of 500 µm and coating speed of ~10 mm s^{-1}, and measured with a grazing incidence angle of 2°.

Additional GIWAXS data (**Supplementary Fig. 24**) was acquired with a SmartLab (RIGAKU) diffractometer with a 3 kW Cu X-ray source (8.04 keV) in parallel beam configuration with 0.5° pinhole optics, a 1° in-plane parallel slit collimator and 0.3° long collimator attachments. Scattering was collected with a HyPix-3000 (RIGAKU) 2D detector with 65 mm SDD, also at α_i = 2°. For each measurement, the detector goniometer arm was rotated through 2θ angles from 0° to 40° in 1° steps, with 15 min acquisition at each position, with detector images then remapped into Q-space and combined together. All data reduction was performed using scripts based on the PyFAI and pygix libraries[10,11].

Scanning Electron Microscopy (SEM)

A FEI Quanta 600 FEG Environmental Scanning Electron Microscope (ESEM) was employed to investigate perovskite layer morphology. Accelerating voltages between 4-15 kV were employed for various analyses.

Nuclear Magnetic Resonance (NMR) Spectroscopy

A two-channel Bruker Avance III HD Nanobay 400 MHz instrument running TOPSPIN 3 equipped with a 5 mm z-gradient broadband/fluorine observation probe is used. The signal from residual non-deuterated DMSO solvent is used for reference.

Nuclear Quadrupole Resonance (NQR) Spectroscopy

For ^{127}I NQR spectroscopy, thin films were mechanically exfoliated from FTO (2D-intermediate $FAPbI_3$) or glass ($FAPbI_3$, DMF:DMSO) substrates using a razor blade to produce powders. These thin film powders were packed into 2.5 mm zirconia rotors and compacted. ^{127}I NQR spectra were recorded on a Bruker Avance III spectrometer equipped with a 2.5 mm CPMAS probe using 42 kHz RF field amplitude, a recycle delay of 0.05 s and with the probe placed outside of any external magnetic field. The rotor was static during the measurement.

Thermal Desorption-Gas Chromatography-Mass Spectrometry (TD-GCMS)

Thin films (<1 μm) of perovskite material on fluorine-doped tin oxide-coated glass substrates were loaded into a thermal extractor unit (Micro-Chamber/Thermal Extractor M-CTE250, Markes International) and heated at 165 °C for 60 minutes under a flow of N_2 gas (50 mL min^{-1}). Volatile components released during extraction were collected onto sorbent-packed collection tubes. The sorbent tubes are then loaded into a thermal desorption unit and heated rapidly to desorb the volatiles concentrated in the tube, which are then passed *via* N_2 carrier gas into the gas chromatography-mass spectrometry instrument (Agilent 5977B GC/MSD). Identification of the volatile components was done by comparison to the NIST 17 Mass Spectral Library. All reported species showed a >85 % match with the database compound.

Optically-Modulated Surface Photovoltage Measurements

Modulated SPV spectra were measured in the configuration of a parallel plate capacitor (quartz cylinder partially coated with the SnO_2:F electrode, mica sheet as insulator), under ambient atmosphere. The SPV signal is defined as the change in the surface potential as a result of the illumination. In our case the Illumination was provided by a Halogen lamp, coupled to a quartz prism monochromator (SPM2), and modulated at a frequency of 8 Hz by using an optical chopper. The overall SPV amplitude (combined of the in-phase and out of phase SPV signals) were detected with a high-impedance buffer and a double phase lock-in amplifier (EG&G 5210).

Characterisation of Solar Cells

Current-voltage (J–V) and maximum power point (MPP) measurements were measured (2400 series source meter, Keithley Instruments) in ambient air under both light (simulated AM 1.5 irradiance generated by a Wavelabs SINUS-220 simulator) and in the dark. The active area of the solar cell was masked with a black-anodised metal aperture to either 0.25 or 1.00 cm^2, within a light-tight holder. The 'forward' J–V scans were measured from forward bias to short-circuit and the 'backward' scans were from short-circuit to forward bias, both at a scan rate of 245 $mV\ s^{-1}$. Active MPP tracking measurements using a gradient descent algorithm were performed for at least 30 s to obtain the steady-state power conversion efficiency. Some cells exhibited improvement over multiple measurements, in which case the peak performance was reported. This typically took two to five J–V scan plus MPP tracking iterations for the highest efficiency cells, in a measurement time of around 2–5 minutes. The intensity of the solar simulator was set periodically such that the short-circuit current density from a KG3-filtered Si reference photodiode (Fraunhofer ISE) matched its 1-sun certified value. A local measurement of the intensity before each batch of solar cell measurements were performed, was made by integrating the spectrum obtained from the solar simulator's internal spectrometer. By taking the ratio of this internal intensity measurement to one obtained at the time of calibration we determined the equivalent irradiance at the time of measurement. For the data presented in this publication, this gave values ranging from 0.985-1.005 suns equivalent, which have been applied to the calculation of power conversion efficiencies for each individual measurement. The spectral mismatch factor was estimated to be 1.022 according to a previously reported method[12]. This has also been applied to calculate power conversion efficiencies. We estimate the systematic error of this setup to be on the order of ± 5% (relative).

External Quantum Efficiency (EQE)

External quantum efficiency (EQE) measurements were performed using a custom setup. The solar cells were illuminated with a 250 W quartz-tungsten halogen lamp that was first passed through a monochromator (Princeton Instruments SP2150) with a filter wheel (Princeton

Instruments FA2448), then chopped with an optical chopper (Thorlabs MC2000B) at 280Hz, and finally focussed onto the sample with a smaller spot size than the solar cell area (as defined by the metallic top contact). The amplitude of the resulting AC current signal was measured with a lock-in amplifier (Stanford Research Systems SR830) as the voltage drop across a 50 Ohm resistor in series with the solar cell. A reference measurement of a calibrated Si photodiode of known EQE (Thorlabs FDS100-CAL) was used to correct the measured signal from the solar cell to absolute units. White light bias was applied using LEDs.

Operational Stability Accelerated-Aging Equipment

PSCs (p-i-n configuration) aged under ISOS-L-1 and ISOS-L-2 conditions were encapsulated using a cover glass and UV-curable epoxy adhesive (Everlight Eversolar AB341), which was spread across the full active area of the PSCs. As discussed in **Supplementary Note 9**, for ISOS-L-2 testing, in addition to the PSC fabrication described above, prior to on-cell encapsulation (where the encapsulation glue is spread across the full device area) a 250 nm layer of MoO_3 was thermally evaporated[13]. For ISOS-D-3 aging, an industry standard edge-sealant was employed to encapsulate PSCs, as described in **Supplementary Note 10**.

For ISOS-D-2 aging, PSCs were not encapsulated and were placed in an oven controlled at 85 ˚C in a N_2 filled glovebox.

ISOS-D-3 aging was carried out in a Weiss Technik LabEvent L C/64/40/3 environmental testing chamber at 85 ˚C and 85% relative humidity (RH).

For adapted ISOS-L-1 aging, a bespoke setup was used. RS Peltier Pro modules were employed to actively cool the PSCs to approximately 22 ˚C (measured by a photodiode encased in a black thermally conductive material to simulate the conditions in the photoactive layer). As shown in **Supplementary Fig. 29b**, an LED array positioned such as to provide 0.86 sun equivalents to the surface of the PSCs was employed. Aging was carried out at V_{oc} under these conditions.

For ISOS-L-2 aging an Atlas SUNTEST CPS+ light-soaking chamber under simulated full spectrum AM1.5 sunlight was employed (no UV filter was applied during the aging process). Aging was carried out at V_{oc} under these conditions. The temperature for the aging chamber was measured by a black standard temperature control unit.

To perform J-V characterisation and stabilised V_{oc}, J_{sc} and active MPP tracking measurements, all substrates were removed from the aging setups and immediately tested before being returned to the relevant aging condition.

2D-intermediate Conversion Schematic (Fig. 3i-k)

The authors highlight that Fig. 3i-k is a schematic representation only, not based on calculations, and constructed using VESTA software using .cif files taken from the publication of Stoumpos, et al.[1415]

Supplementary Note 1: The solvent system

The integral role of precursor solution chemistry in controlling the outcome of perovskite solution deposition has often been overlooked. Research has repeatedly shown that coordination complexes formed dynamically in such solutions directly influence the nucleation and growth of the perovskite layer, and thus on its crystallinity, morphology and optoelectronics[16–18]. Direct crystallisation of precursor solutions has demonstrated that metal-solvent coordination interactions are sufficiently robust to be retained into the solid-state and are therefore presumed to likewise play an important role controlling haloplumbate intermediates in parent solutions[19]88. A range of approaches to pseudo-quantify solvent coordinating ability have been reported, including dielectric constant, Hansen solubility parameters, Mayer bond order and the Gutmann donor number (D_N) (Supplementary Table 1). Although no single metric is expected to perfectly characterise the complex range of precursor-solvent interactions, perovskite solvent systems reported to-date appear to be best represented by D_N[20,21]. This suggests that the Lewis acid-base interaction between metal and solvent is the most significant for precursor dissolution and control of subsequent solution-state interactions. Moreover, out of the drive towards DMF-free processing, new and general models for perovskite solvation are beginning to crystallise. In reporting their DMSO/ACN/2-methoxyethanol (2-ME) ternary solvent mixture, Deng, et al., identified the role of two solvent classes; DMSO as a non-volatile coordinating solvent and both ACN and 2-ME as volatile non-coordinating solvents[22]. The key differentiator between solvents of these two classes is D_N (Supplementary Table 1). Although we contest Deng, et al.'s claim that ACN and 2-ME should be considered non-coordinating (as these solvents are at least capable of dissolution-reprecipitation type processes and thus catalyse the conversion of perovskite precursors, AX + BX_2, into ABX_3 powders) – and so prefer the terms poorly Pb^{2+}-coordinating solvent (PPS) and strongly Pb^{2+}-coordinating solvent (SPS) – we recognise in this analysis the potential for a general family of co-solvent systems, to which all our low-D_N PPS:high-D_N amine systems also belong. Critically, however, in contrast to other high-D_N coordinating solvents, the amines

used here are either included – *via* a Brønsted Lowry H^+ exchange in solution – as bulky ammonium cations within the perovskite structure or volatilised upon deposition due to their relatively high vapour pressure. In either case the need for subsequent antisolvent engineering is precluded. Doolin, et al., recently reported another example of SPS-PPS mixed solvent systems[23]. The authors present three quaternary solvent systems based on non-stoichiometric mixtures of DMSO, dimethylpropyleneurea (DMPU), ethanol (EtOH) and MeTHF; notably the first and presently only report of MeTHF use in the context of metal halide perovskites to-date. In this case, DMPU supports DMSO as an alternative SPS while MeTHF and EtOH serve as PPSs. Notably, in all three mixtures at least 70 vol % SPS was required, as compared with typically < 15 vol % BA in the systems presented herein.

Supplementary Table 1 | Perovskite solvents. Table summarising solvents reported to date commonly used in dissolution of lead (II) salts during lead halide perovskite solution processing. D_N = Gutmann donor number, MBO = Mayer bond order, ε = dielectric constant, δ_P = Hansen solubility parameter for dipolar intermolecular interactions, NME = nitromethane

Solvent	D_N / kcal mol^{-1}	MBO	ε	δ_P / MPa$^{1/2}$
BA	42.0	-	4.92	-
DMPU	33	-	36.1	-
DMSO	29.8	1.50	46.7	16.4
NMP	27.3	1.90	32.2	12.3
DMF	26.6	1.88	36.7	13.7
THF	20.0	-	7.6	-
GBL	18.0	1.99	40.2	16.6
ACN	14.1	3.03	37.5	18.0
MeTHF	12.0	-	7.0	-
NME	2.7	-	35.9	-

Supplementary Note 2: Solvents for scalable processing

A critical factor that has often been missed in assessing the suitability of a solvent for solution-processing applications is that the majority solvents necessarily must go through the vapour phase during extraction from the deposited layers. The more efficient the deposition technique is in minimising volume of wasted precursor ink, the higher the relative volume of solvent that must be vaporised (i.e. not lost as liquid phase precursor solution). The important implication of this observation is that most available solvent selection guides – which are typically based on pharmaceutical applications, where solvents are expected to remain in the liquid phase – are inappropriate aides in the selection of scalable solvent systems for solution processing.

Two commonly cited solvent selection guides are presented by Prat, et al.[24,25], and have been relied upon in a number of perovskite green solution processing reports[23,26,27]. However, Prat, et al., introduce a 'Safety' rating for common pharmaceutical solvents, which is principally based on the solvents' flash points; the lowest temperature at which its vapours ignite in the presence of an ignition source. Critically, the flash point is strongly dependent on the vapour pressure of the solvent. The higher the vapour pressure, the higher the proportion of a given volume of the (liquid) solvent that is expected to exist as vapours at the surface of the liquid, at a given temperature. As a result, solvents with high vapour pressures (THF, MeTHF, ACN) score poorly on such safety ratings. However, as the majority of liquid solvents – regardless of vapour pressure – must pass into the vapour phase during the solution processing of a thin film by scalable deposition techniques, flash point is clearly an inappropriate measure of relative solvent safety.

There is certainly an inflammation hazard associated with vaporised solvents. However, the most relevant vapour pressure-independent measure is the lower flammability limit (LFL), measured as the lowest v/v % of the flammable solvent in air that's capable of igniting. To our knowledge, no LFL has yet been determined for MeTHF, but for vast majority of common solvents (DMSO, DMF and THF included) the LFL is between 1 and 4 % (Supplementary Table 2).

Moreover, besides LFL the other key flammability concern for solution processing is the autoignition temperature (T_a) of solvent (mixtures) employed. As Supplementary Table 2 shows, the T_a for DMSO is relatively close to its boiling point. Wang, et al., have also reported that the onset temperature for exothermic decomposition events in solutions of DMSO is reduced by other chemicals including halides and a number of acids and bases[28]. Along with the skin-penetrating enhancement of DMSO, these findings show how – despite its widely positive reception – there are significant concerns regarding the use of this solvent in at-scale solution processing.

Supplementary Table 2 | Scalable perovskite solution processing. Parameters of significance for scalable solution processing for a range of solvents relevant to this work. LFL = lower flammability limit, T_a = autoignition temperature, PDE = permitted daily exposure. Note: a high value of 'Safety Score' corresponds to nominally 'less safe' solvent (see discussion above).

Solvent	Prat, et al., Safety Score [24]	Boiling Point / °C [24]	Vapour Pressure / hPa at 20 °C [29]	Flash Point / °C [24]	LFL / % by volume of air [30]	T_a / °C [30]	PDE / mg day^{-1} [31,32]
**THF**	6	66	200	-14	2	321	7.2
**MeTHF**	6	80.2	136	-11	unknown	270	50
**DMSO**	1	189	3.5	95	2.6	215	-
**DMF**	3	153	0.6	58	2.2	445	8.8
***n*-butanol**	3	118	5.8	35	1	343	>50

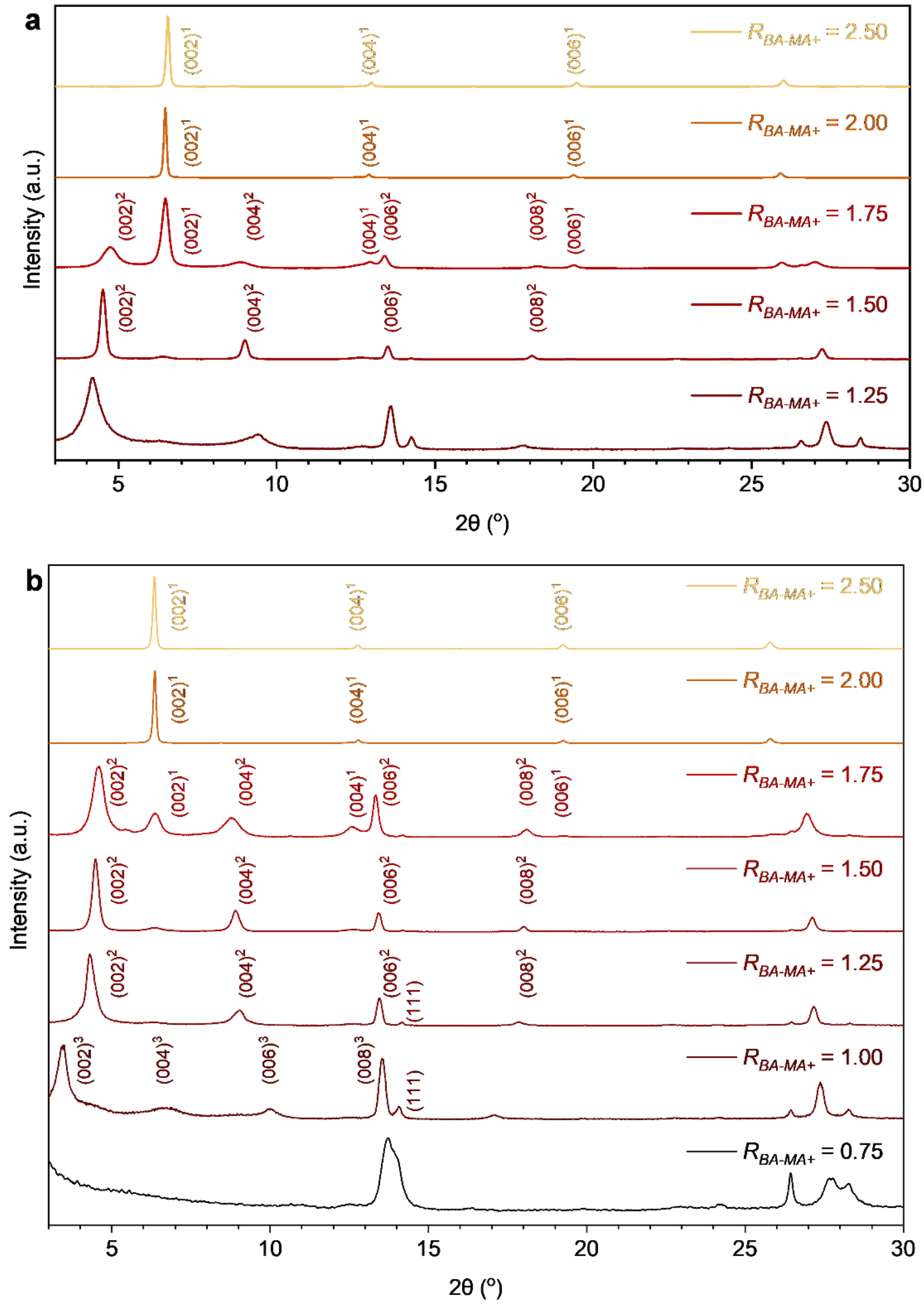

Supplementary Figure 2 | Mixed-phase 2D intermediates. 1D X-Ray Diffraction (XRD) patterns corresponding to phase-mixed 2D RPP ($BA_2MA_{n-1}Pb_nI_{3n+1}$) layers processed from MeTHF (**a**) and THF (**b**). $R_{BA\text{-}MA+}$ = BA/MA$^+$. Lower order reflections have been labelled with the (hkl) set of planes that gives rise to them, from a given pure *n* 2D RPP phase. $(00l)^n$ corresponds to a diffraction signal caused by the (00l) set of planes in the nth 2D RPP phase.

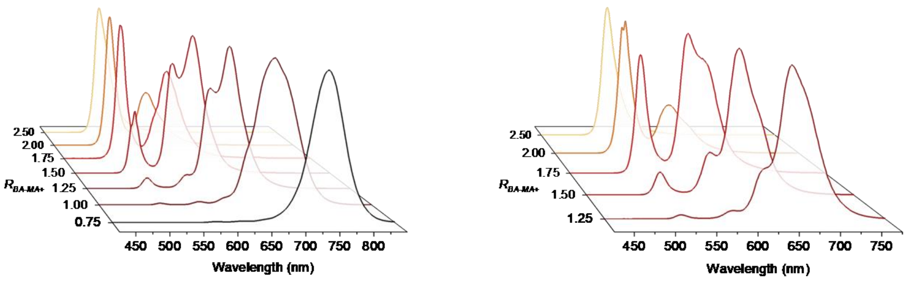

Supplementary Figure 3 | Mixed-phase 2D intermediates. Steady-state photoluminescence (SSPL) emission spectra (normalised) of phase-mixed 2D RPP layers deposited *via* THF (left) and MeTHF (right) with varying $R_{BA\text{-}MA+}$.

The inconsistency in apparent phase composition between SSPL and X-ray diffraction (XRD) (**Supplementary Fig. 2**) measurements is likely due to a combination of photon-recycling effects and charge carrier transfer (funnelling) to electronically-connected, lower energy states, leading to lower bandgap phases being overrepresented in emission spectra.

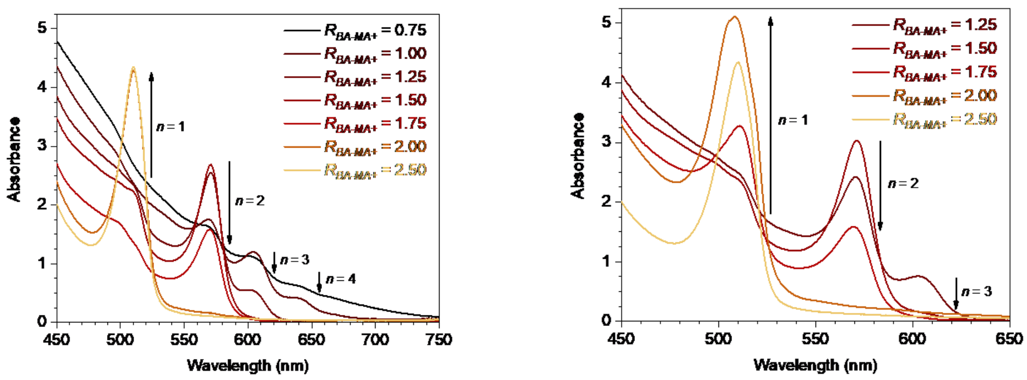

Supplementary Figure 4 | Mixed-phase 2D intermediate. Absorbance spectra of phase-mixed 2D RPP layers deposited *via* THF (left) and MeTHF (right) with varying $R_{\text{BA-MA+}}$. Arrows indicate general trend in phase composition as $R_{\text{BA-MA+}}$ is increased.

Supplementary Note 3: Relating $R_{BA\text{-}MA+}$ to <*n*>

<*n*> is determined by considering the molar ratio of BA^+ to MA^+ in the deposited RPP material. As such, although correlated, the precise relationship between $R_{BA\text{-}MA+}$ and the value of <*n*> for a given phase mixture is not straightforward. The inequivalent pK_{aH}'s of BA and MA and the apparent preferential formation of the thermodynamically favoured n = 1 phase in the RPP layers[99] result in significantly greater BA^+ inclusion than suggested by the precursor stoichiometry ($R_{BA\text{-}MA+}$). By dissolving RPP layers processed with varying $R_{BA\text{-}MA+}$ in deuterated (d_6) DMSO we conduct ^{1}H solution Nuclear Magnetic Resonance (NMR) spectroscopy measurements (Supplementary Fig. 4). From integration of signals corresponding to BA and MA we determine the BA^+:MA^+ stoichiometry in the deposited RPP layers and thus calculate true <*n*> values. A full description of the relationship between <*n*> and BA^+:MA^+ stoichiometry is provided below. For the convenience of the reader, however, in this work we describe the mixed-phase RPPs in terms of $R_{BA\text{-}MA+}$.

For a 2D phase mixture containing phases $n = 1, 2, 3 \ldots \mathrm{p}$, with each phase present in corresponding mole fraction $a, b, c \ldots q$ such that:

$$a + b + c + \cdots + q = 1 \tag{1}$$

where a is the mole fraction of $n = 1$ phase present, b is the mole fraction of $n = 2$ phase present, etc., then overall the phase mixture may be represented as:

$$< n > = a + 2b + 3c + \cdots + pq \tag{2}$$

From our ^{1}H NMR experiments we are able to determine, by integration of the signals corresponding to MA's and BA's methyl groups (δ = 2.38 (s), 0.89 (t) ppm, respectively), the molar proportion of BA^+ to MA^+ present in each 2D RPP intermediate, which we denote R_{exp}. Thus, for the 2D RPP family $BA_2MA_{n-1}Pb_nI_{3n+1}$, this quantity may be calculated as:

$$R_{exp} = \frac{[BA^+]}{[MA^+]} = \frac{2a + 2b + 2c + \cdots + 2q}{b + 2c + \cdots + [p-1]q} \tag{3}$$

Combining Eq. 1 and Eq. 3:

$$R_{exp} = \frac{2}{b + 2c + \cdots + [p-1]q} \quad (4)$$

Thus, by expressing Eq. 2 in the form:

$$< n > = (a + b + c + \cdots + q) + (b + 2c + \cdots + [p-1]q) \quad (5)$$

and combining Eq. 1, Eq. 4 and Eq. 5, it is clear that:

$$< n > = 1 + 2{R_{exp}}^{-1} = 1 + \frac{2[MA^{+}]}{[BA^{+}]} \quad (6)$$

for any mixture of RPP phases.

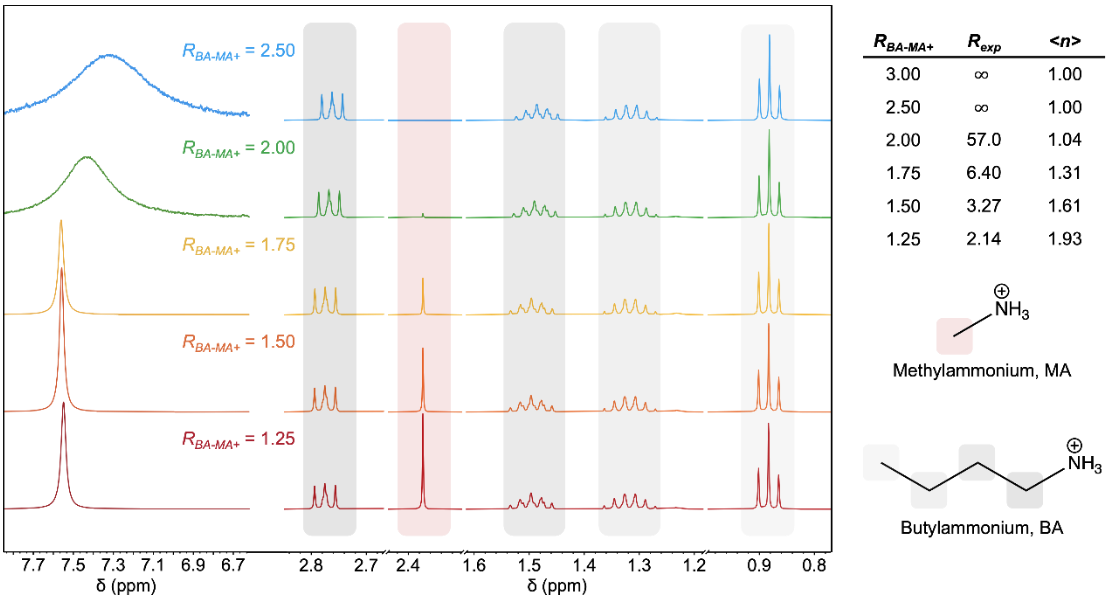

$R_{BA\text{-}MA+}$	R_{exp}	<n>
3.00	∞	1.00
2.50	∞	1.00
2.00	57.0	1.04
1.75	6.40	1.31
1.50	3.27	1.61
1.25	2.14	1.93

Supplementary Figure 5 | Determining 2D intermediate composition. ^{1}H solution Nuclear Magnetic Resonance (NMR) spectra of mixed-phase $BA_2MA_{n-1}Pb_nI_{3n+1}$ RPP layers dissolved in DMSO-d_6. Integration of the signals corresponding to MA's and BA's methyl groups (δ = 2.38 (s), 0.89 (t) ppm, respectively) confirm the reduction in BA:MA ratio in the deposited thin films as $R_{BA\text{-}MA+}$ is reduced, and are used to calculate the true molar ratio of BA^{+} to MA^{+} in the deposited RPP material (R_{exp}). From this analysis we calculate the true <n> value of the mixed-phase layers.

Supplementary Fig. 5 maps the true values for <n> calculated by applying Eq. 6 to the experimental data provided in Supplementary Fig. 4. In the region $R_{BA\text{-}MA+} \leq 2$ the incorporation of MA^+ – despite the thermodynamic preference for the n = 1 phase (containing no MA^+), and despite a film growth environment in which more than sufficient BA^+ is available to crystallise the thermodynamically favoured n = 1 phase (note: the *absolute* molar quantities of all precursors in the 50 uL of precursor solution used in spin-coating the 2D RPP layers is much greater than those found in the subsequently deposited layer, suggesting none is ever limiting during crystallisation other than in a kinetic regime) – suggests that crystallisation is kinetically controlled and thus that composition is a function of the relative likelihood of the immediate coordination environment of Pb^{2+} ions in the precursor solution containing BA^+ or MA^+ cations. As pK_{aH} (BA) < pK_{aH} (MA) we expect for $R_{BA\text{-}MA+} > 2$ the Pb^{2+} coordination environment to contain on average at least 2 BA^+ cations and so formation of the thermodynamically preferred n = 1 phase is not kinetically prohibited. Thus, the experimentally determined values for <n> in this region all reflect near phase-pure n = 1 RPPs.

For $R_{BA\text{-}MA+} \leq 2$, we expect the Pb^{2+} coordination environment to contain on average < 2 BA^+ cations and so crystallisation under kinetic control results in the incorporation of a proportion of MA^+, despite this being thermodynamically disfavoured. Fitting of the region where $R_{BA\text{-}MA+} \leq 2$ shows a strong linear relationship with slope = -1.19. A slope of -1 would imply equal preference for MA^+ and BA^+ in this BA^+-limited regime. Instead, the slope suggests that for Pb^{2+} centres at which crystallisation of the preferred n = 1 phase is not possible, MA^+ is preferentially incorporated into the n > 1 phases leading to their being over-represented at $R_{BA\text{-}MA+} \leq 2$. We speculate that this is likely a kinetic effect. The globularity of MA^+ renders its inclusion in the growing perovskite phase significantly more likely than BA^+, as the latter cation is far less likely to be appropriately oriented at the instant of crystallisation.

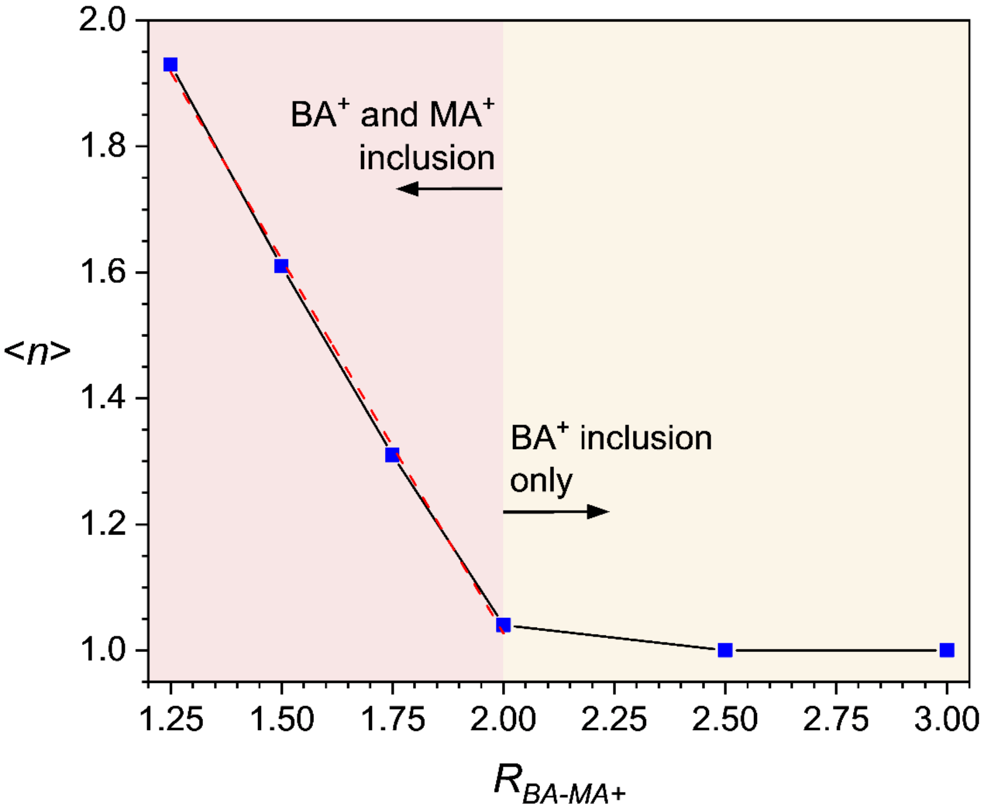

Supplementary Figure 6 | Controlling 2D intermediate composition. Relationship between BA:MA+ precursor stoichiometry, $R_{BA\text{-}MA+}$, and average *n* phase of 2D RPP materials, <*n*>.

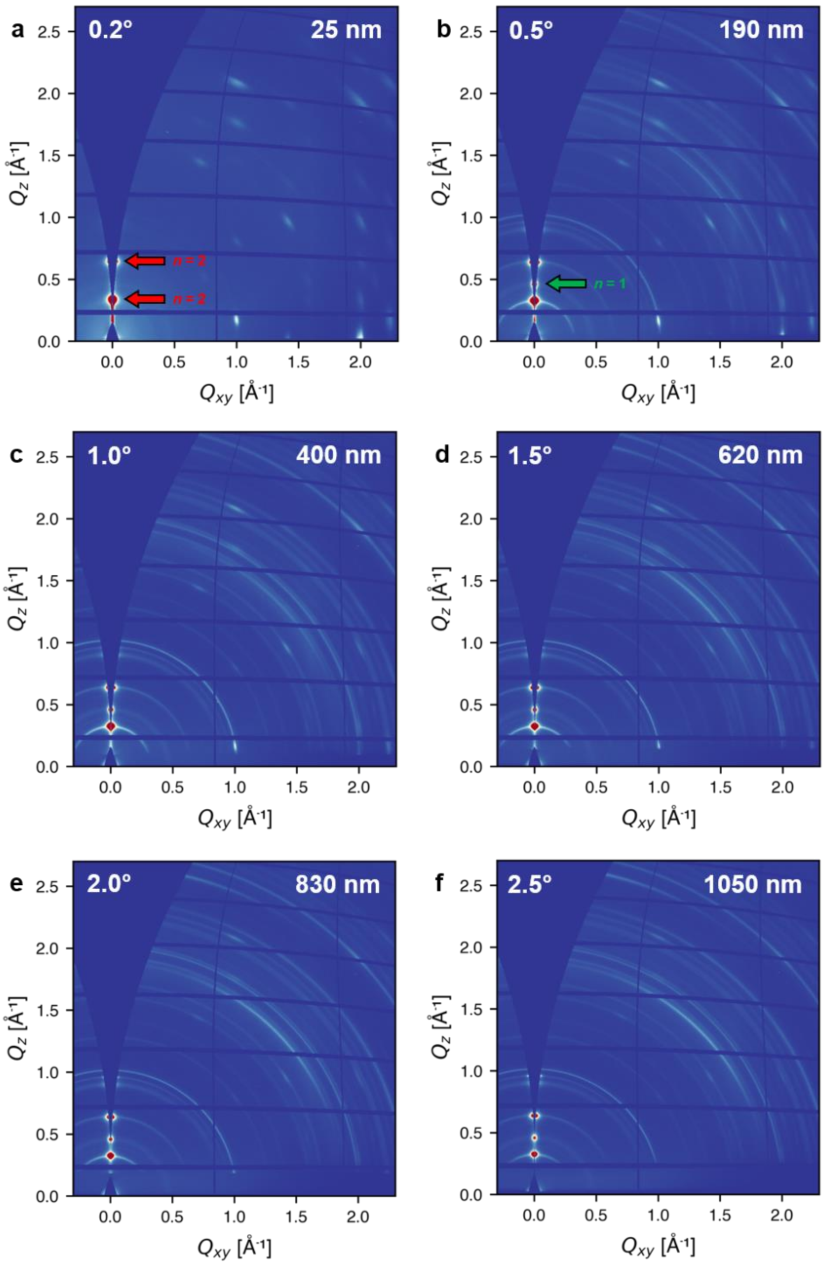

Supplementary Figure 7 | Vertical phase distribution of mixed-phase 2D intermediates. Grazing incidence wide-angle X-ray scattering (GIWAXS) diffraction patterns of the optimised 2D perovskite intermediate, collected at increasing angle of incidence (**a-f**; 0.2°, 0.5°, 1.0°, 1.5°, 2.0°, 2.5°, respectively), thus probing increasing depths into the perovskite film and giving an indication of any vertical phase distribution.

We observe that at the surface of the intermediate only diffraction spots corresponding to the n = 2 ($BA_2MAPb_2I_7$) phase are resolved. This may indicate that the surface of the 2D intermediate is n = 2 terminated, with the n = 1 (BA_2PbI_4) phase appearing only at greater depths into the thin film. However, it may also be that the out of plane intensity of the n = 1 phase is not observed as it falls entirely inside the missing wedge at such low incidence angles. It is certainly the case, however, that throughout the remainder of the film the 2D intermediate is highly uniform.

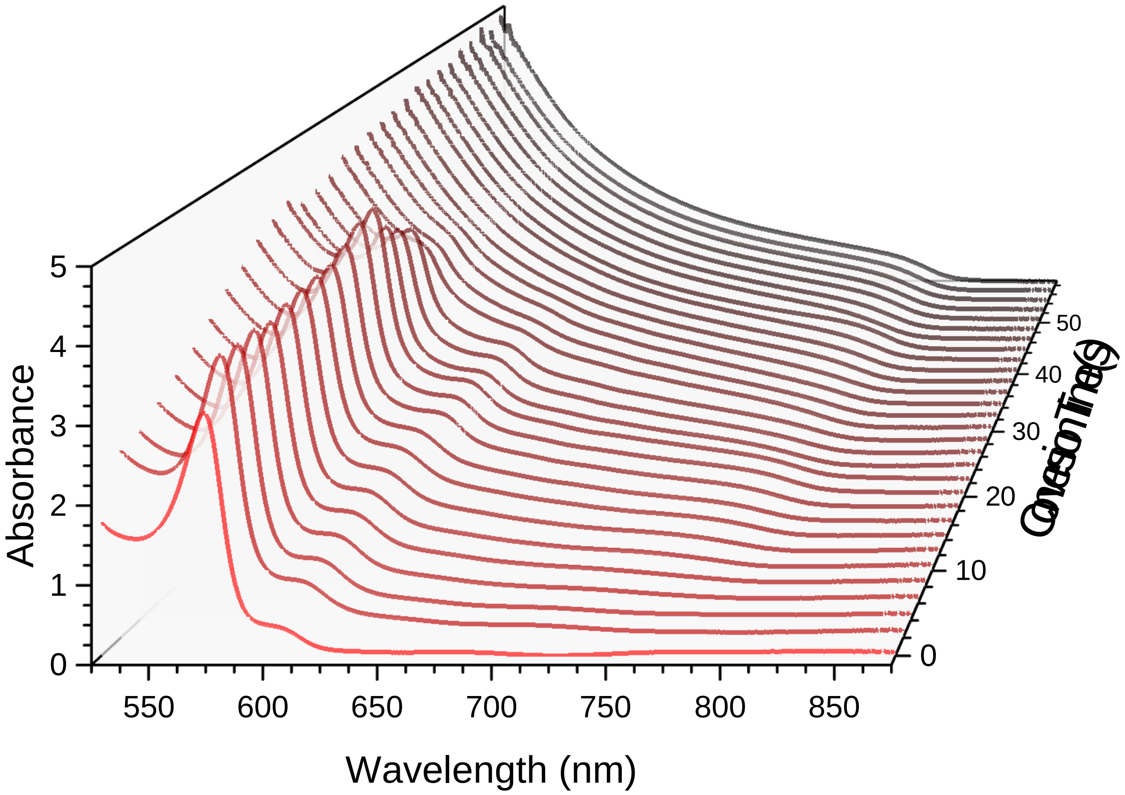

Supplementary Figure 8 | Evolution of 2D intermediate to 3D perovskite. Tracking absorbance of optimised 2D intermediate ($R_{BA\text{-}MA+}$ = 1.5) converting to 3D α-$FAPbI_3$ during soaking in conversion solution, prior to thermal curing. Unconverted 2D intermediate shown at t = 0 s.

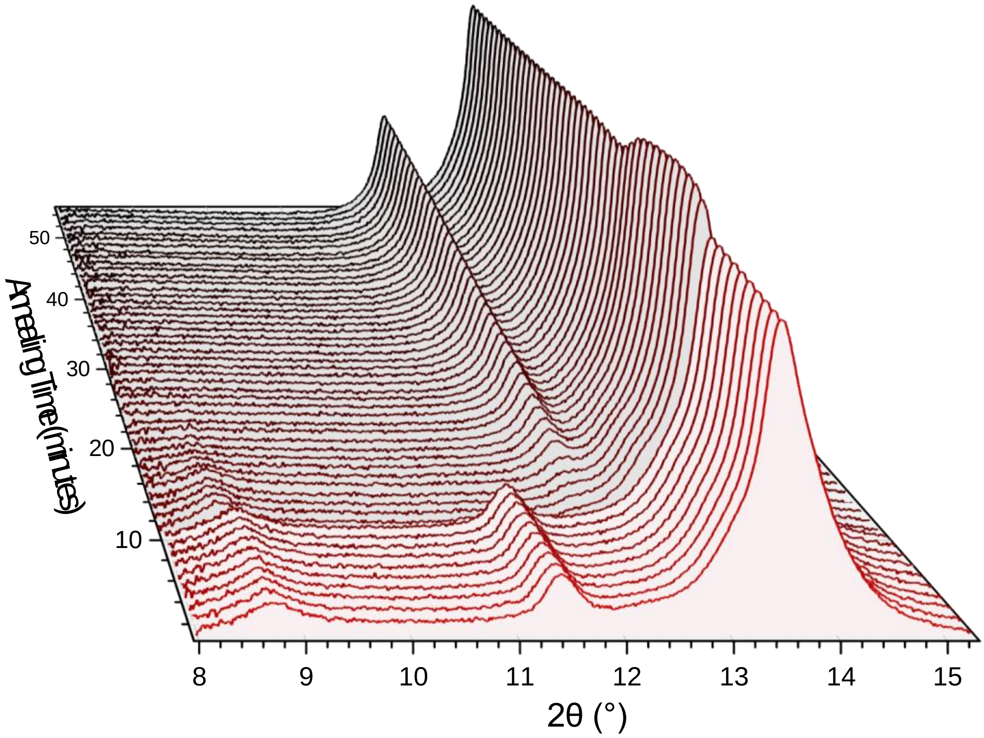

Supplementary Figure 9 | Thermally-driven 2D-to-3D conversion. In-situ X-Ray diffraction (XRD) patterns tracking removal of remnant 2D material and subsequent ripening of 2D-intermediate ($R_{BA\text{-}MA+}$ = 1.5) α-$FAPbI_3$ under thermal annealing at 70 °C for 10 minutes, followed by 180 °C for 55 minutes. Intensity is plotted vertically as a log scale.

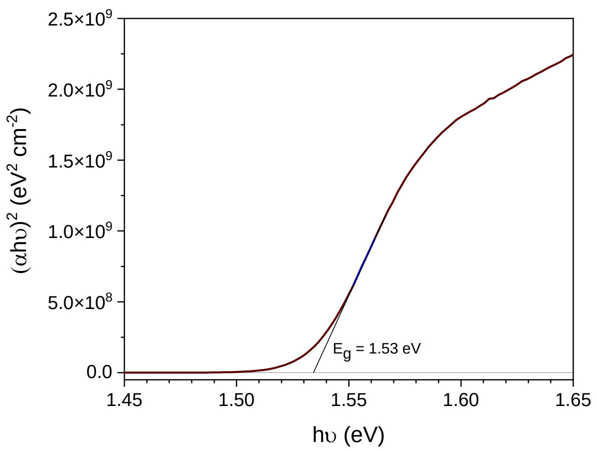

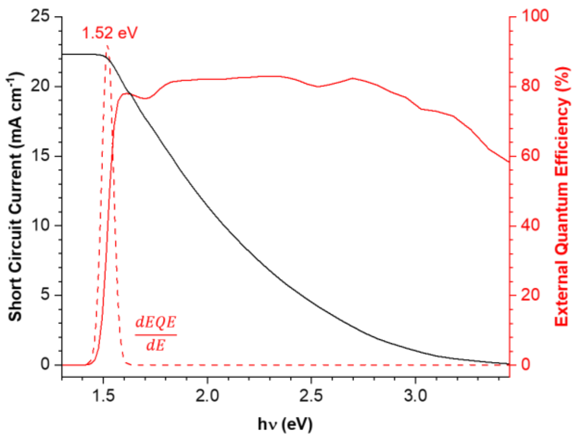

Supplementary Figure 10 | Single junction-compatible bandgap. Left, Extraction of the optical bandgap of 2D-intermediate α-$FAPbI_3$ *via* a Tauc analysis. **Right**, External quantum efficiency (EQE) and integrated short circuit current (J_{SC}) of high-performance PSC based on 2D-intermediate α-$FAPbI_3$. A photovoltaic bandgap ($^{PV}E_g$) of 1.52 eV is demonstrated for the perovskite material based on a fitting of the first derivative of the EQE. We note that the device from which this EQE spectrum was obtained was not our champion device, but instead displayed a stabilised power conversion efficiency of 18.3 %.

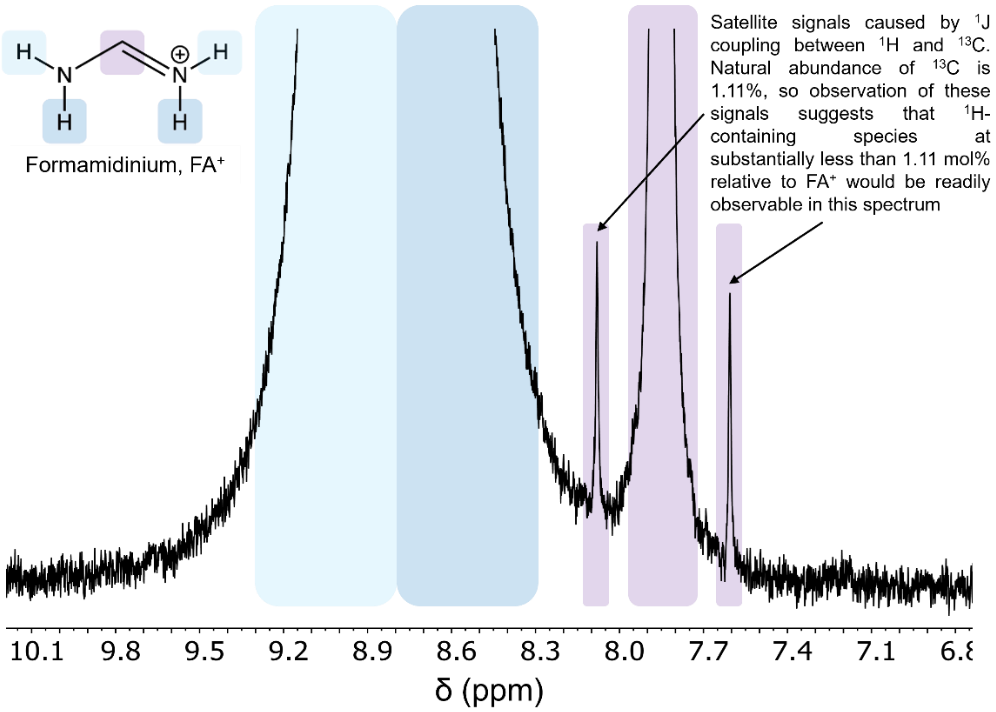

Supplementary Figure 11 | ^{1}H solution NMR sensitivity. Formamidinium region of ^{1}H solution nuclear magnetic resonance (NMR) spectra of α-$FAPbI_3$ thin films dissolved into DMSO-d_6 (also shown in **Fig. 2d**), showing that the intensity of ^{13}C satellite signals around the FA^+ methine signal gives an indication of sensitivity of this experiment to trace ^{1}H-containing species, such as MA^+ and BA^+.

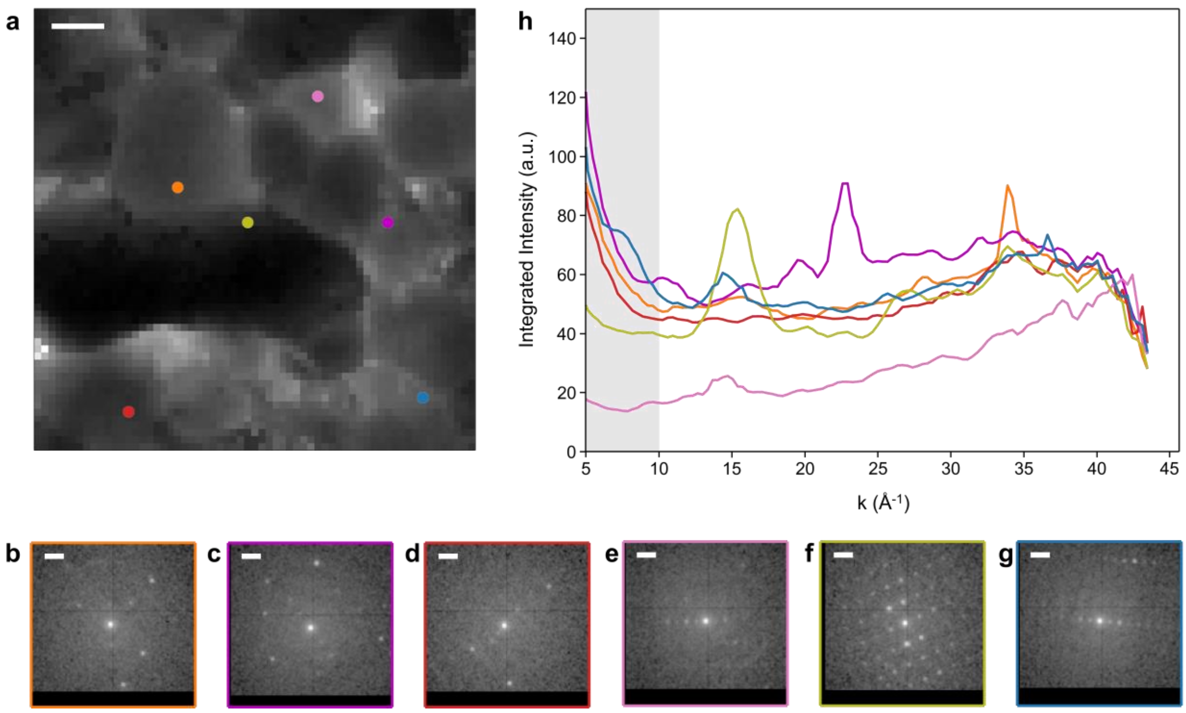

Supplementary Figure 12 | Scanning electron diffraction (SED) measurements of 2D-intermediate $FAPbI_3$. Sample is fabricated on an oxygen plasma-treated SiN substrate (with 10 nm thick SiN window in centre) using a spin program with faster maximum spin speed (7,500 rpm) than typical when depositing the 2D intermediate layer, to generate a thinner (~250 nm thick) 2D perovskite layer. After conversion, the sample is annealed for 10 minutes at 70 °C and then for 30 minutes at 180 °C to drive complete conversion to the 3D perovskite. (**a**) Virtual ADF (VADF) image for diffraction signal in 5-10 $Å^{-1}$ range (shaded region). Scale bar: approximately 200 nm. (**b-g**) Diffraction patterns corresponding to regions highlighted with coloured dots in **a**. These patterns have been aligned using the centre-of-mass method in Pyxem. Scale bar: 8.5 $Å^{-1}$. (**h**) Radial average of diffraction patterns (b-g) show no signal at low (<15 $Å^{-1}$) inverse distances, indicating the absence of 2D phases.

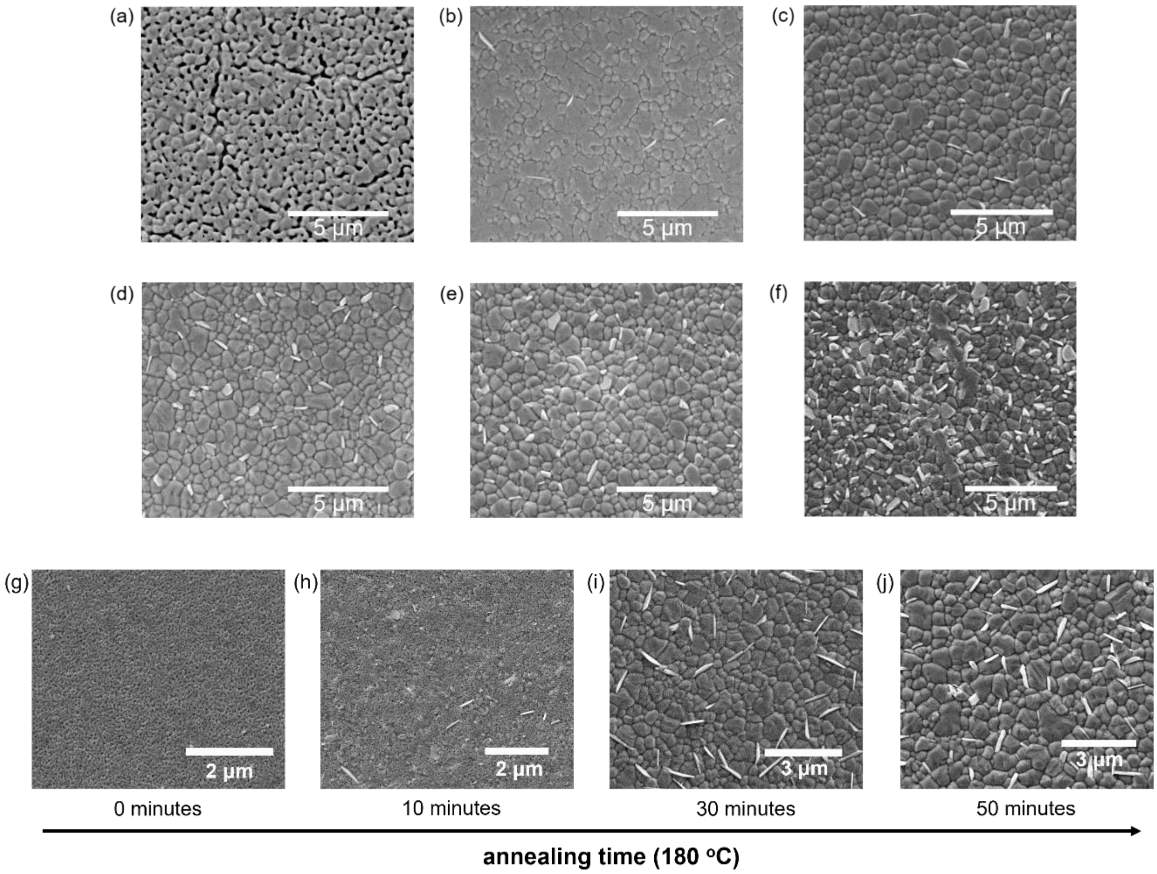

Supplementary Figure 13 | Intermediate- and annealing time-dependent morphology. Scanning Electron Microscopy (SEM) images showing the microstructure of 2D-intermediate α-$FAPbI_3$ layers when a series of 2D intermediates processed from MeTHF with varying $R_{BA\text{-}MA+}$ are converted sequentially. $R_{BA\text{-}MA+}$ = 2.50 (**a**), 2.00 (**b**), 1.75 (**c**), 1.66 (**d**), 1.50 (**e**), 1.25 (**f**). Images shown below (**g-j**) correspond to thin films ($R_{BA\text{-}MA+}$ = 1.66) annealed for different periods of time at 180 °C and indicate substantial changes in morphology associated with substantial grain ripening processes occurring at high temperature.

As discussed in Supplementary Note 1, the reduced coordinating ability of MeTHF (Gutmann Donor Number, D_N = 12.0) compared with THF (D_N = 20.0) results in a practical limit of precursor solubility. When MeTHF serves as the PPS in a 0.75 M precursor solution, [BA]/[PbI_2] ≥ 1.25 mol / mol is required for complete solvation, compared to a limiting [BA]/[PbI_2] ≥ 0.75 mol / mol with THF. Although we observe no characterizable difference in perovskite materials processed *via* MeTHF or THF at equivalent BA/PbI_2, the lower achievable [BA]/[PbI_2] stoichiometries of the THF system allow access to higher <*n*> 2D RPP phase mixtures, which are essential for fully elucidating the important role of <*n*> in effective 2D-intermediate conversion to 3D perovskites (Fig. 3).

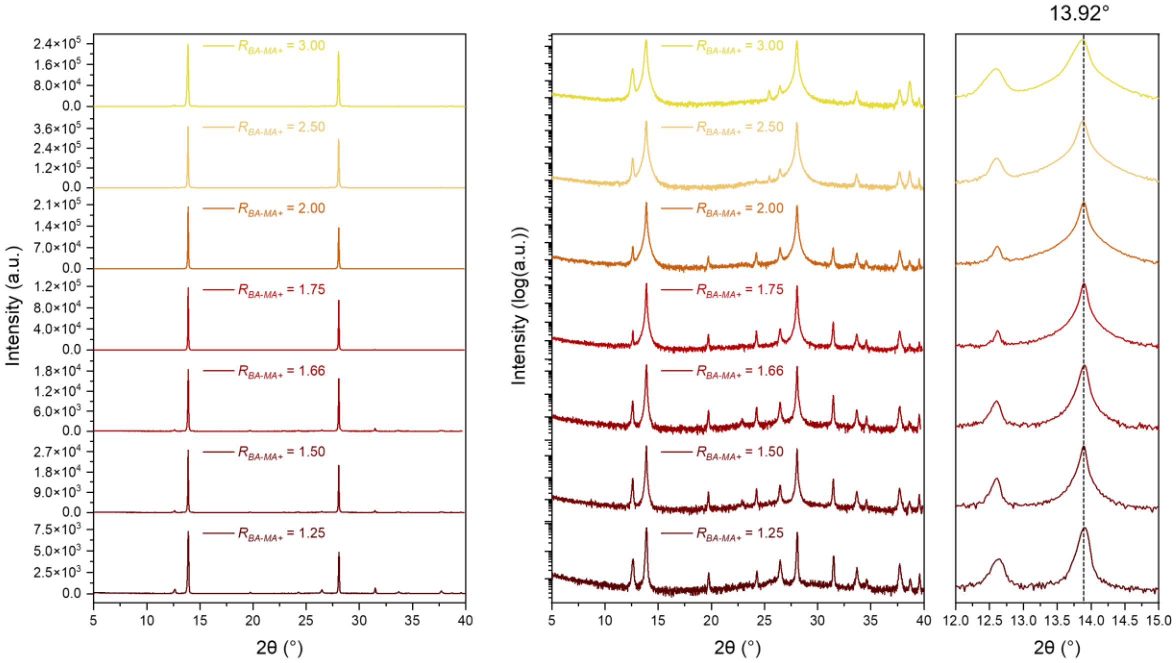

Supplementary Figure 14a | 2D-intermediate-dependent lead (II) iodide content. X-ray diffraction (XRD) patterns of 2D-intermediate α-$FAPbI_3$ layers produced when a series of 2D precursor phases processed from MeTHF with varying R_{BA-MA+} are converted sequentially. Left: Full XRD patterns. Middle: Semi-log *y* plot of the same data set, highlighting the absence of phases peaks corresponding to phases other than α-$FAPbI_3$ and *6R*-PbI_2. Right: Semi-log *y* plot highlighting the region of the patterns corresponding to the (100) and (001) sets of planes in α-$FAPbI_3$ and *6R*-PbI_2, respectively. The dashed line is positioned at 2θ = 13.92°, corresponding to the (100) of α-$FAPbI_3$.

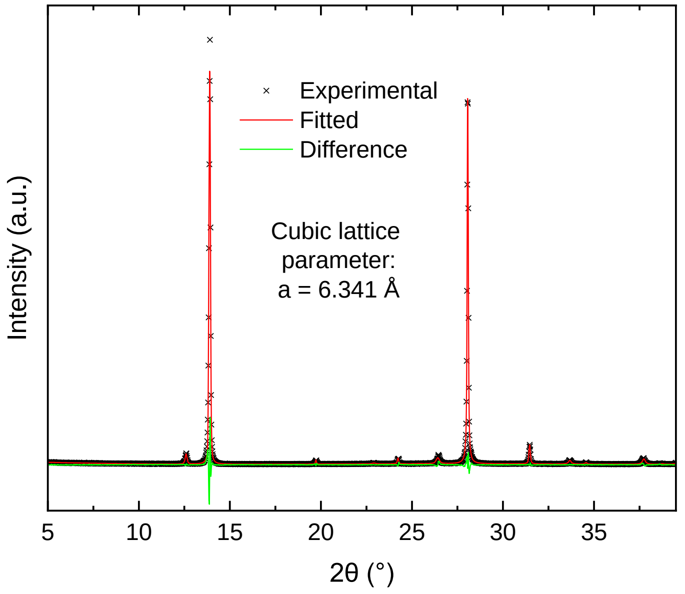

Supplementary Figure 14b | Pawley fit of optimised α-$FAPbI_3$ thin film XRD pattern. Fitting applied to R_{BA-MA+} = 1.66 thin film material processed from MeTHF (shown in **Supplementary Fig. S14a**). Fit confirms the presence of only α-$FAPbI_3$, *6R*-PbI_2 and fluorine-doped tin oxide (FTO; substrate) phases. Cubic lattice parameter for α-$FAPbI_3$ phase, a = 6.341 Å.

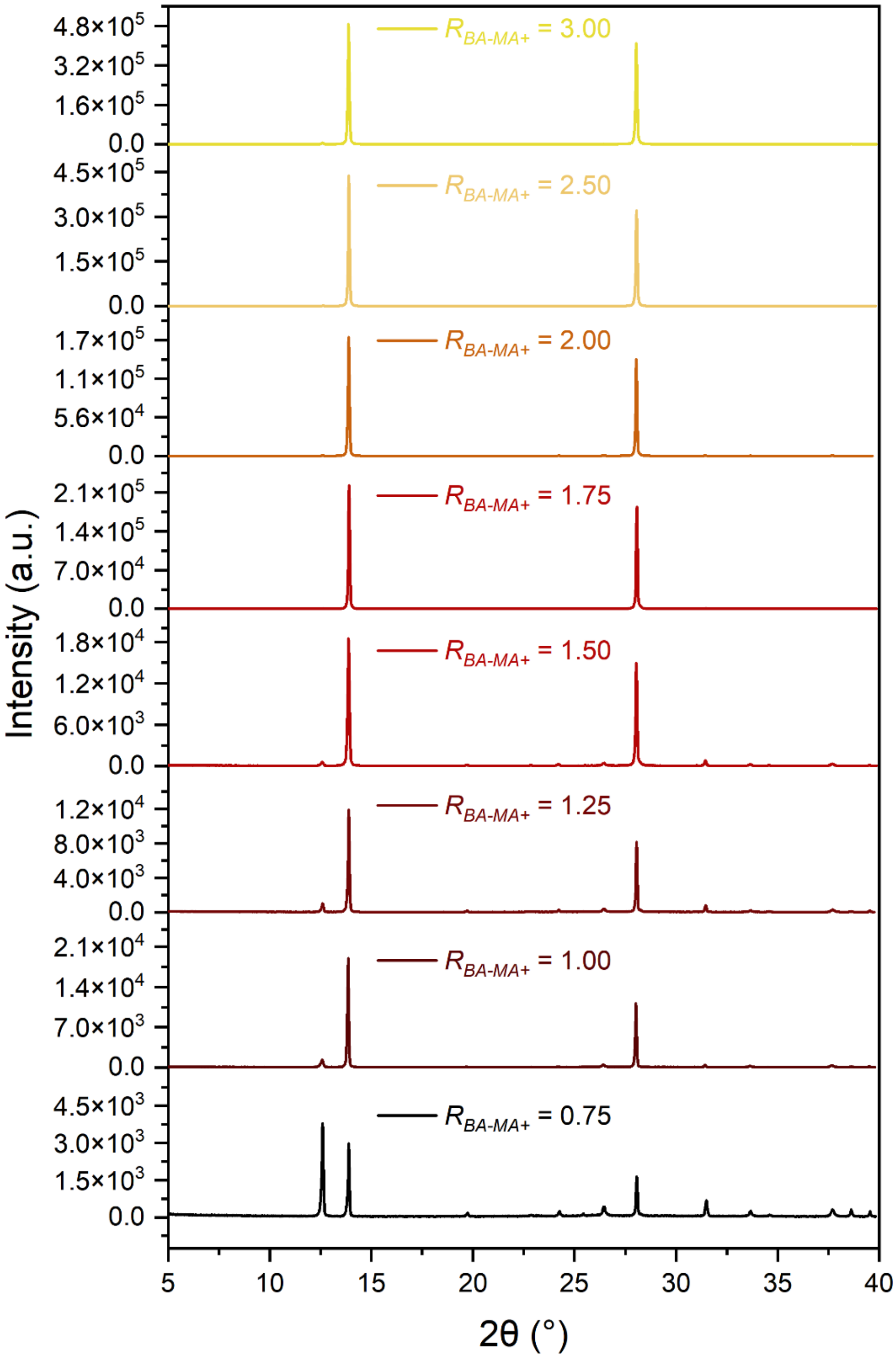

Supplementary Figure 15 | Intermediate-dependent lead (II) iodide content. X-ray diffraction (XRD) patterns of 2D-intermediate α-$FAPbI_3$ layers produced when a series of 2D intermediates processed from THF with varying $R_{BA\text{-}MA+}$ are converted sequentially.

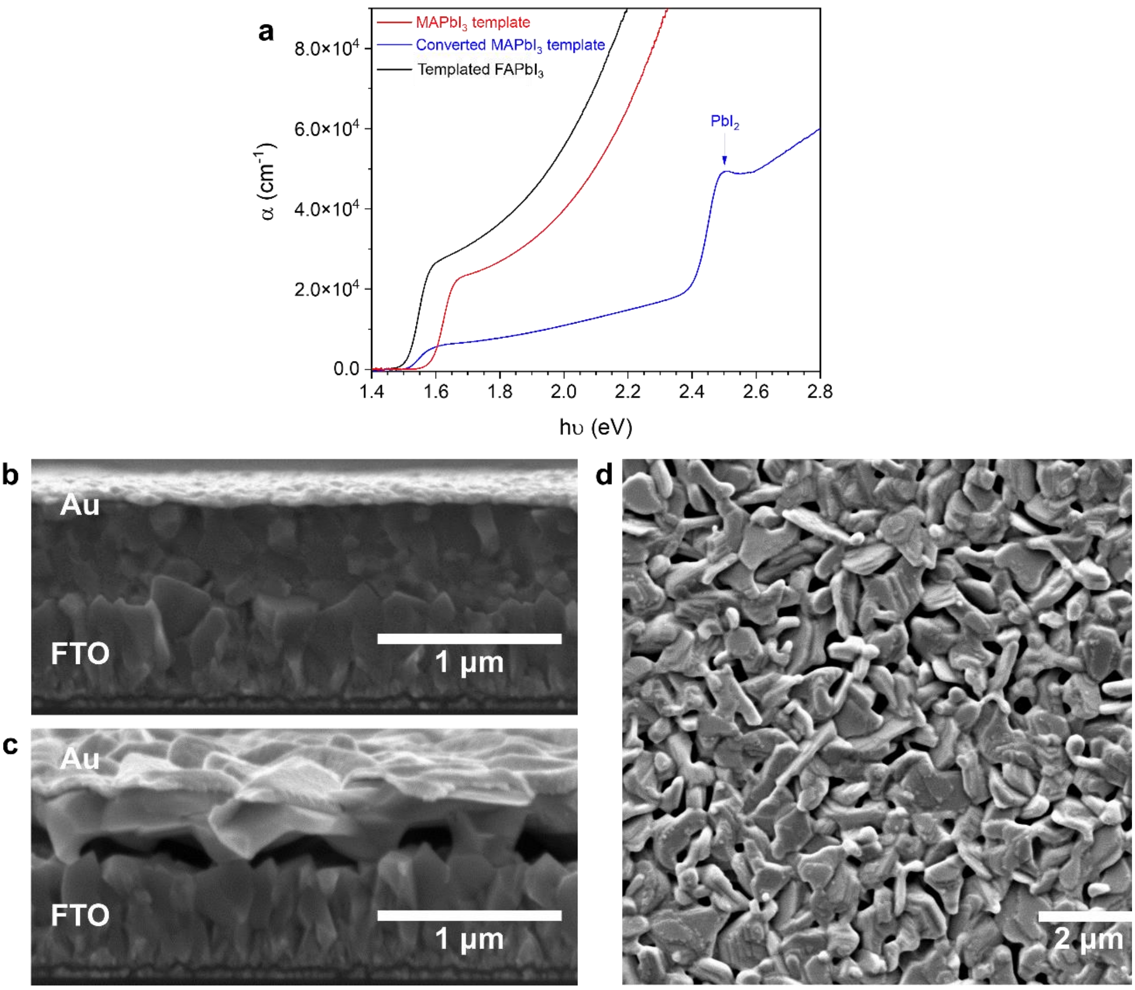

Supplementary Figure 16 | Unsuccessful *n* = ∞ ($MAPbI_3$) intermediate. a, Absorption coefficient spectra corresponding to a $MAPbI_3$ layer (500 nm, processed *via* acetonitrile/methylamine gas) (red) serving as the *n* = ∞ member of the $BA_2MA_{n-1}Pb_nI_{3n+1}$ family of intermediates and undergoing an attempted conversion to α-$FAPbI_3$ (blue) (cured at 180 °C for 30 minutes). 2D-intermediate α-$FAPbI_3$ is shown for comparison (black). The band-edge of converted $MAPbI_3$ intermediate indicates that complete conversion to lower-bandgap $FAPbI_3$ has not been achieved. Moreover, the appearance of an absorption feature corresponding to PbI_2 indicates incomplete conversion has been achieved. Cross-sectional scanning electron microscopy (SEM) images of $MAPbI_3$ intermediate (**b**) and converted $MAPbI_3$ intermediate (**c**). **d**, Top view SEM image of converted $MAPbI_3$ intermediate.

Supplementary Note 4

To further investigate the mechanism of 2D precursor phase conversion we next assess the impact of 2D precursor phase composition on the resulting 3D perovskite. Scanning electron microscopy (SEM) images of α-$FAPbI_3$ processed *via* 2D precursor films of varying <*n*> reveal a wide variety of microstructures. Use of a high $R_{BA\text{-}MA+}$ (BA:MA^+ precursor stoichiometry) 2D precursor phase (low <*n*>) results in a mesoporous structure in the 3D perovskite film (Fig. 3a-b), and even macroscopic cracking of the thin-films (Fig. 3a, inset). XRD analysis of α-$FAPbI_3$ processed from intermediate $R_{BA\text{-}MA+}$ ratios reveals how gradual reduction of $R_{BA\text{-}MA+}$ (toward higher <*n*>) leads to a pronounced increase in lead (II) iodide (*6R*-PbI_2) content in the as annealed films (Fig. 3l, Supplementary Fig. 15). We observe a consistent trend with the appearance of hexagonal *6R*-PbI_2 platelets, which appear brighter in the SEM images (Fig. 3c-g)[34]. Conversion from high-*n* 2D precursor phase films also leads to mesoscopic porosity visible *via* SEM (Fig. 3h). However, in contrast to low-*n* precursor phases, XRD analysis of high-*n*-converted 3D materials demonstrates significant *6R*-PbI_2 content (Fig. 3i). We rationalise these results by considering the careful balance of factors that control optimum <*n*> for 2D precursor phase conversion and identify a 'Goldilocks region' in which optimal composition and morphology are achieved.

Ideally BA^+ content in the RPP layer should be minimised to both diminish the need for subsequent BA^+ expulsion and minimize the volume change upon conversion to α-$FAPbI_3$. The low lead halide density of 2D precursor films rich in the n = 1 phase demands significant volume contraction to form a continuous network of 3D corner-sharing $[PbI_6]^{4-}$ octahedra. Where the strain required for uninterrupted fusion of adjacent 2D lead-halide layers into a 3D perovskite structure is large, this may be relieved by pores and cracks opening within the perovskite film, at the expense of increased surface area and hence surface free energy. Fig. 3i presents schematically how such features may occur. Observation of mesoscopic cracking in Fig. 3a-b thus underscores the importance of minimising volume contraction by employed 2D intermediates containing n > 1 phases.

Mechanistic rationalisation of the microstructures observed in α-$FAPbI_3$ processed from high n (low $R_{BA\text{-}MA+}$) intermediates is more involved. However, we propose that two controlling criteria are critical: organic channel density and the thickness of the corner-sharing haloplumbate layers in the 2D precursor phase (i.e. the $\langle n \rangle$ value). To demonstrate the integral role organic channels play in effecting conversion, we attempt the conversion of neat $MAPbI_3$ (representing $(BA^+)_2(MA^+)_{n-1}Pb_nI_{3n+1}$ with $n = \infty$) with the same 0.1 M FAI solution in *n*-butanol. Supplementary Fig. 16a shows the absorbance spectra of this '$MAPbI_3$ precursor phase' and material resulting from its attempted conversion. The presence of an absorption feature corresponding to PbI_2 in the converted layer, and the blue-shifted absorption onset compared to α-$FAPbI_3$, indicate that complete conversion of the $MAPbI_3$ intermediate has not been achieved. This is as expected; *n*-butanol does not significantly disrupt inorganic haloplumbate structures, thus cation intercalation and extraction are expected to be facilitated by – and occur most rapidly *via* – the organic channels found in our 2D perovskite precursor phase films. Thus, reduction of organic channel density by reducing BA^+ content in the precursor phase (towards low $R_{BA\text{-}MA+}$) limits cation exchange, ultimately leading to severely limited conversion from the $MAPbI_3$ intermediate. In Supplementary Fig. 16b-c we show cross-sectional SEM images of the $MAPbI_3$ precursor phase film, and the converted film, revealing the formation of significant voids at the bottom of the perovskite layer during conversion.

In addition to the effect of reduced spacer molecule channel density, the thicker corner sharing lead halide octahedra layers found in higher n RPPs render cation exchange more challenging. For $n > 2$ phases, complete extraction of MA^+ from and intercalation of FA^+ cations into the intact inorganic scaffold requires migration across multiple perovskite A-sites, penetrating through unbroken layers of $[PbI_6]$ octahedra that act as an energetic barrier to this ion intercalation. Any MA^+ not extracted from the intermediate during conversion might be expected to volatilise during subsequent thermal curing at 180 °C, *via* mechanisms discussed in Supplementary Note 8. As Fig. 3k describes schematically, such a process would yield Pb^{2+} and I^--rich regions in the forming 3D perovskite, and ultimately regions of crystalline PbI_2 upon

grain ripening during thermal curing. The occurrence of such ripening is evidenced in the substantial evolution in morphology observed during high temperature curing (Supplementary Fig. 14g-j). This proposed mechanism is not only consistent with the gradual increase in PbI_2 content observed in converted $1.00 \leq R_{BA\text{-}MA+} \leq 1.75$ precursor phase films, but also justifies the extreme increase in PbI_2 observed for $R_{BA\text{-}MA+} = 0.75$. XRD of the same series of 2D perovskites (Supplementary Fig. 2) revealed that the $R_{BA\text{-}MA+} = 0.75$ precursor phase is the only one to contain majority $n \geq 4$ RPP phases. The volatilisation of remnant MA^+ and subsequent PbI_2 formation is corroborated by our *in-situ* GIWAXS measurements, which show an increase in *6R*-PbI_2 formation during the first 5 minutes of high temperature annealing followed by minimal change in PbI_2 XRD peak intensity thereafter when all remnant MA^+ has been lost (Fig. 2b).

Furthermore, similarly to the effect observed for low-*n* precursor phase films, volume contraction caused by conversion from high-*n* 2D perovskites to PbI_2-rich material may also lead to mesoscopic cracking on account of the more densely packed Pb^{2+}/I^- in the lead (II) iodide unit cell compared with mixed-phase $(BA^+)_2(MA^+)_{n-1}Pb_nI_{3n+1}$ perovskites[35,36]. Thus, our proposed mechanism is also consistent with the microstructure observed for $R_{BA\text{-}MA+} = 0.75$ (Fig. 3h). PbI_2 formation and associated volume contraction is likely also responsible for the voids observed at the bottom of our converted '$MAPbI_3$ intermediate', and microstructure observed in top view SEM (Supplementary Fig. 16b-d).

We find by profilometer measurements that the thickness of our optimum 2D precursor phase film ($R_{BA\text{-}MA+} = 1.5$; predominantly $n = 2$) contracts from 1,050 nm to 725 nm upon conversion to α-$FAPbI_3$; a 32% volume contraction. Calculations using the reported unit cell volume of $BA_2MAPb_2I_7$ (3,118.7 Å^3)[15], which contains eight PbI_6^{4-} octahedra, and that of $FAPbI_3$ (256.4 Å^3)[37], indicate that a theoretical volume contraction of approximately 34% should be observed. These data suggest that the majority of volume contraction upon conversion occurs in the vertical plane rather than laterally, which might otherwise lead to substantial strain in the thin film if not released by voiding. Recently, Doherty, et al. reported a strategy to assess

desymmetrisation of the cubic haloplumbate network in α-$FAPbI_3$ by octahedral tilting using ^{127}I nuclear quadrupole resonance (NQR) spectroscopy[38]. ^{127}I NQR transition frequencies depend on the electric field gradient across ^{127}I nuclei in the material. Due to the very large electric quadrupole moment of ^{127}I, small structural changes result in considerable changes in electric field gradient, and thus NQR frequency. $FAPbI_3$ possessing a perfectly cubic structure is expected to exhibit a single, narrow NQR peak. Where even a small range of structurally inequivalent ^{127}I environments exist within $FAPbI_3$, superposition of slightly different transitions leads to substantial broadening of the NQR peak. Loss of local cuboctahedral symmetry in $FAPbI_3$ due to microstrain, or by trace X-site or A-site mixing[39] (e.g. incorporation of MA^+) causes broadening of the NQR peak, as both such modes lead to a range of inequivalent ^{127}I environments with differing degrees of local asymmetry. Similarly, non-uniform tilting throughout the material, such as that described by Doherty, et al. is expected to lead to broadening of the NQR peak by the same mechanism. On the other hand, uniform octahedral tilting of $[PbI_6]^{4-}$ octahedra throughout a $FAPbI_3$ sample leads to tetragonal symmetry and the formation of two inequivalent ^{127}I environments, and thus two resolvable NQR transitions[40]. A similar effect is expected in a material exhibiting macrostrain. Consistent distortion throughout the $FAPbI_3$ structure in one direction leads to two or more inequivalent ^{127}I environments, and thus multiple distinct NQR transitions. In Figure 3m, we compare ^{127}I NQR of $FAPbI_3$ thin films made by a conventional DMF:DMSO solution processing route (in the absence of any additives) with 2D precursor phase-engineered $FAPbI_3$ thin films. The single narrow peak exhibited by 2D precursor phase $FAPbI_3$ is compelling evidence that I^- in the structure is present in a regular cubic network, since deviation from this due to any of the phenomena discussed above is expected to lead to peak-splitting or broadening. Importantly, this result eliminates the possibility of any form of residual strain in the material. Interestingly, we do observe broadening and a slight shift in the NQR transition maximum (maximum corresponds to the average electric field gradient at the ^{127}I nuclei) of $FAPbI_3$ fabricated from DMF:DMSO, which we attribute to reduced crystallinity and increased disorder in the material, likely due in part to entrapment of low-volatility solvents inside the material, as discussed below.

The mechanics of 2D precursor phase conversion and growth require significant further investigation. However, overall it is clear that, unlike intermediates featuring coordinating solvents or other lower-dimensionality materials reported to-date[41–43], the <*n*> > 1 RPPs reported here hold two critical advantages as sequential deposition precursor phases for 3D perovskites: an extended corner-sharing haloplumbate structure already present in the precursor phase ready to serve as a scaffold for 3D perovskite growth, as evidenced by the direct conversion of 2D perovskite into α, not δ, phase $FAPbI_3$; and built-in organic channels within the lead-halide scaffold that can facilitate rapid intercalation of a conversion solution containing A-site cations. As we show, the presence of the pre-existing corner-sharing lead-halide scaffold is imperative for effective conversion, minimising structural rearrangement and volume contraction during formation of the 3D cubic perovskite.

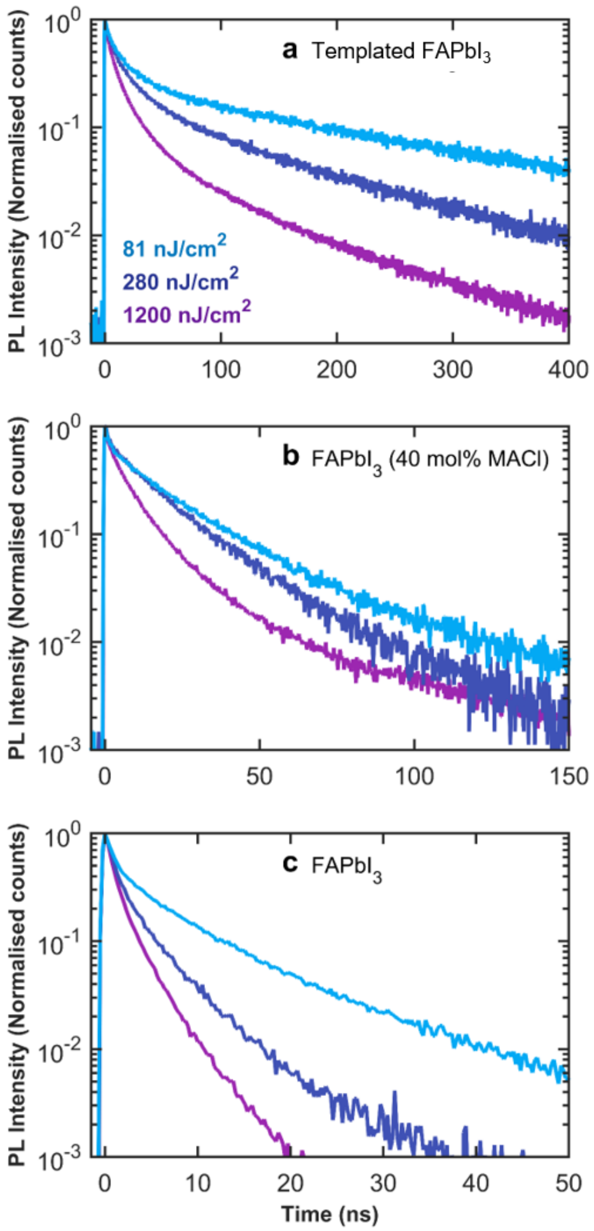

Supplementary Figure 17a-c | Time-resolved photoluminescence. Fluence dependence of the PL transients after 398-nm excitation for thin films of 2D-intermediate $FAPbI_3$ (**a**), MACl-additive $FAPbI_3$ (**b**), and additive-free $FAPbI_3$ (**c**). The time scale is changed for composition for best visual clarity of the fluence-dependence transients. The legend in **a** also applies to **b**, and **c**.

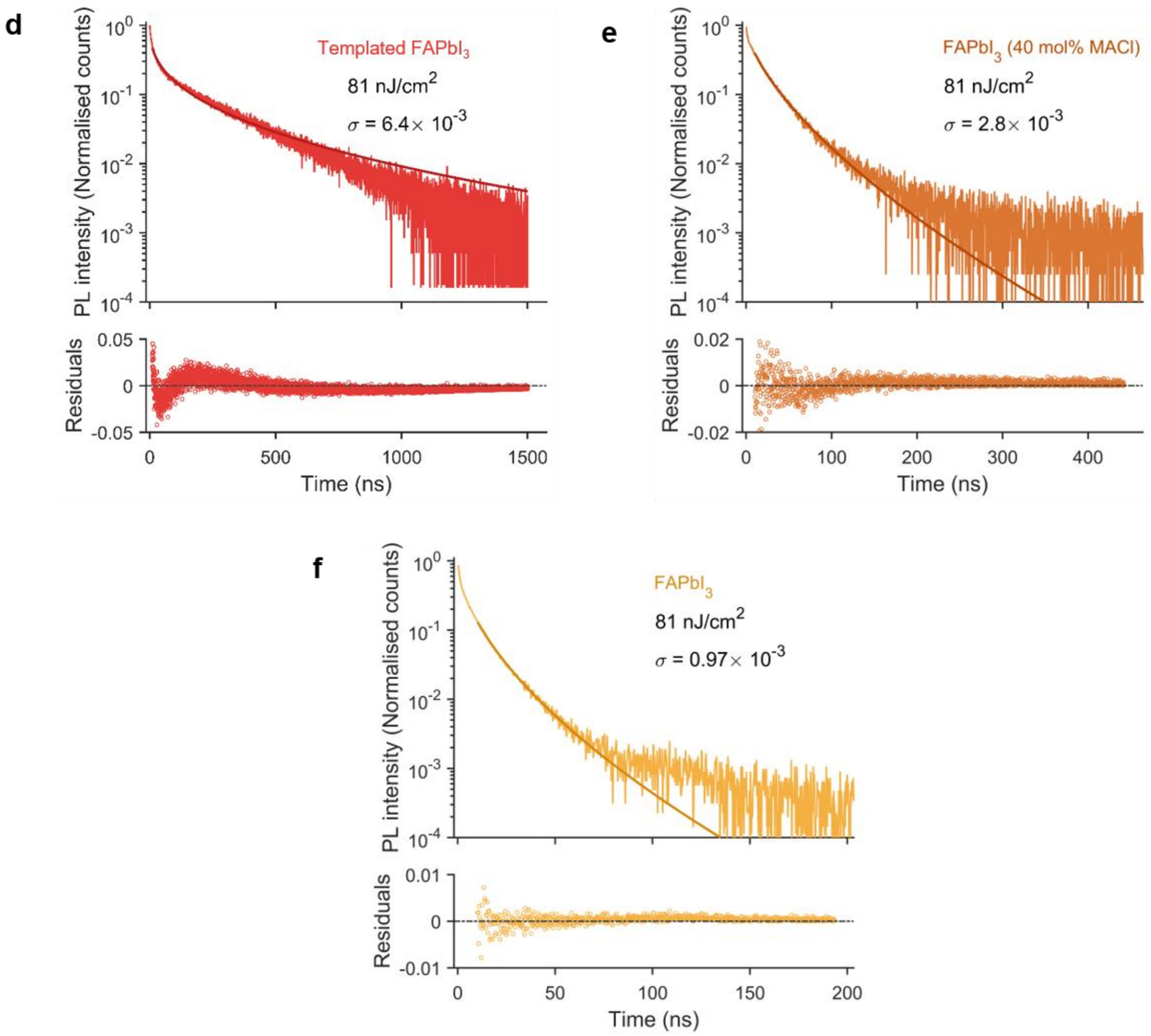

Supplementary Figure 17d-f | Stretch exponential fits of time-resolved photoluminescence of $FAPbI_3$. Stretch exponential fit to the time-resolved photoluminescence of 2D-intermediate $FAPbI_3$ (**d**), MACl-additive $FAPbI_3$ (**e**), and additive-free $FAPbI_3$ (**f**) after a 398-nm excitation.

Supplementary Note 5: Optical-Pump Terahertz-Probe (OPTP) Spectroscopy

OPTP spectroscopy allows us to non-invasively probe the dielectric response of a medium and examine the charge carriers that modify the material's conductivity at THz frequencies after a well-defined time delay after optical excitation. This approach allows us to investigate the local charge-carrier dynamics and quantify the effective mobilities and bimolecular recombination rates of the photogenerated charge carriers.

Charge-carrier sum mobility

Time-resolved variations in the THz transmission ($\Delta T/T$) through the sample can be measured by varying the time delay between the pump and probe beams, and this change in electric-field transmission at the peak of the THz pulse (t_{gate}) can be in turn related to the change in the photoconductivity (ΔS) of the thin film (films whose thickness *d* is much smaller than the THz wavelengths λ_{THz}), using:

$$\Delta S = -\epsilon_0 c(n_a + n_b)\frac{\Delta T(t_{\text{gate}} = 0)}{T} \quad (7)$$

where $\epsilon_0 c$ is the invert of the vacuum impedence, and n_a and n_b are the refractive indices of the mediums surrounding the sample (vacuum and the substrate).

The charge-carrier mobility (μ) and change in photoconductivity (ΔS) have a linear relationship that holds for low fluences (< 50 μJ cm^{-2}), described using:

$$\mu = \frac{\Delta S A_{\text{eff}}}{Ne} \quad (8)$$

where A_{eff} is the effective area of overlap between the optical pump and the THz probe, and *N* is the number of photoexcited charge carriers.

The number of photoexcited charge carriers can be calculated using:

$$N = \Phi\frac{E\lambda}{hc}(1 - R_{\text{pump}} - T_{\text{pump}}) \quad (9)$$

where Φ is the branching ratio of free charge-carriers to photons absorbed, $(1 - R_{\text{pump}} - T_{\text{pump}})$ is the fraction of the 400 nm pump beam absorbed by the sample, λ and *E* are the wavelength and pulse energy of the excitation pulse, and $\frac{E\lambda}{hc} = N_{\text{ph}}$ is the number of photons in a pump pulse.

We can then combine Eq. 7, Eq. 8, and Eq. 9 to calculate the effective charge-carrier mobility as:

$$\Phi\mu = -\epsilon_0 c(n_a + n_b)\frac{A_{\text{eff}}hc}{Ee\lambda(1 - R_{\text{pump}} - T_{\text{pump}})}\left(\frac{\Delta T\left(t_{\text{gate}}, t_{\text{pump-probe}} = 0\right)}{T}\right) \quad (10)$$

This effective mobility is the effective sum mobility of photogenerated electrons and holes, and it reflects the true sum mobility when the photons absorbed are fully converted to free charge carriers ($\Phi = 1$)

Transient decay fitting

The ΔS transients can be globally fitted to extract k_2, with k_1 *and* k_3 either ignored (set to 0) or set to a reasonable value, as the excitation fluences used for the experiments conducted here lead the fluence-dependent decays over the first nanosecond after excitation to be primarily affected by bimolecular electron-hole recombination captured by the rate constant k_2.

Charge-carrier dynamics in perovskite semiconductors can be generally described using:

$$\frac{dn}{dt} = -k_1 n - k_2 n^2 - k_3 n^3 \quad (11)$$

where *n* is the photogenerated charge-carrier density, k_1 represents the monomolecular recombination rate constant, associated with trap-mediated recombinations, k_2 represents the bimolecular electron-hole recombination, and k_3 represents Auger recombination. Here, trap-mediated recombination rates are too low to have any influence over the first nanosecond (hence k_1 may be ignored), while charge-carrier densities were kept below the limit for which Auger recombination begins to dominate (k_3 may be ignored).

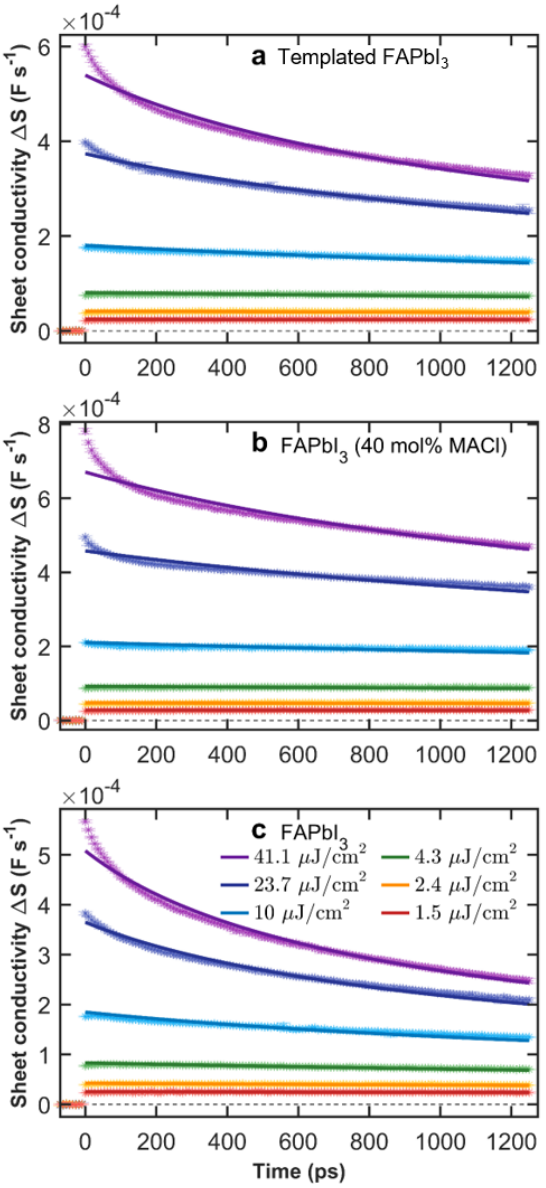

Supplementary Figure 18 | Photoconductivity dynamics. Optical-pump THz-probe photoconductivity transients for 2D-intermediate $FAPbI_3$ (**a**) MACl-additive $FAPbI_3$ (**b**), and additive-free $FAPbI_3$ (**c**). Solid lines are the global fits to solutions of the rate equation expressed in Eq. 11. The legend in **c** also applies to **a** and **b**.

This leads to the analytical expression for *n*:

$$n(t) = \frac{1}{(\frac{1}{n_0} + k_2 t)} \tag{12}$$

where n_0 is the initial number density of free charge carriers at $t = 0$.

Using Eq. 8 and Eq. 10, the photogenerated charge-carrier density is proportional to the change in the photoconductivity (ΔS):

$$n = \Phi \, \mathrm{C} \, \Delta S(t) \tag{13}$$

where C = $\tilde{n}/\Delta S_0$ is the proportionality constant between the initial change in photoconductivity (ΔS_0) and absorbed photon density ($\tilde{n}$), given by:

$$\tilde{n} = \frac{E\lambda}{hcdA_{\mathrm{eff}}}(1 - R_{\mathrm{pump}} - T_{\mathrm{pump}}) \tag{14}$$

Substituting Eq. 14 into Eq. 11 gives:

$$\frac{d(\Delta S)}{dt} = -\Phi k_2 C \, (\Delta S)^2 \tag{15}$$

The global fits across all fluences to this ODE are used to extract the effective bimolecular recombination rate constant Φk_2. Similar to the calculated mobilities, the values presented for k_2 are underestimates for the true intrinsic values.

To account for the spatially-varying charge-carrier density across the thickness of the film, the fitting algorithm considers an exponentially decaying charge-carrier density; done by dividing the film into 30 equally-thick slabs and individually computing the decay function for each slab.

Finally, the diffusion length L_{D} can then be calculated by using the diffusion coefficient *D* (calculated from the mobility) and recombination rate R_{n} (calculated from the recombination rates k_1, k_2, and k_3):

$$L_{\mathrm{D}} = \sqrt{\frac{D}{R_{\mathrm{n}}}} = \sqrt{\frac{k_{\mathrm{B}} T \mu}{e(k_1 + k_2 n + k_3 n^2)}} \tag{16}$$

where *T* is the temperature, and k_B is the Boltzmann constant, and *n* is the charge-carrier density photogenerated under normal operating conditions (1 sun) equalling roughly to $1x10^{15}$ cm^{-3}, for which k_2 and k_3 make only small contributions.

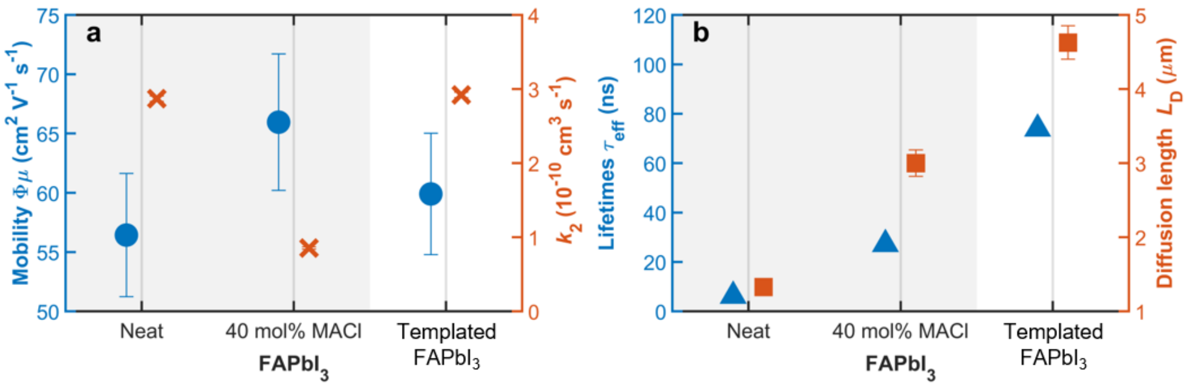

Supplementary Figure 19 | Extracted parameters of charge-carrier dynamics. Charge-carrier recombination and mobility parameters extracted from OPTP (**a**), and TRPL (**b**) spectroscopy. Effective mobilities $\Phi\mu$ and the bimolecular recombination rate k_2 are extracted from the measured onset value of the photoconductivity, and from global fits to the photoconductivity transients shown in Supplementary Fig. 18, respectively. PL lifetimes τ_{eff} are extracted from the TRPL fits in Supplementary Fig. 17 (at an excitation fluence of 81 nJ cm^{-2}) and diffusion lengths L_D are derived under normal solar operating conditions of charge-carrier densities of n ~ 10^{15} cm^{-3}.

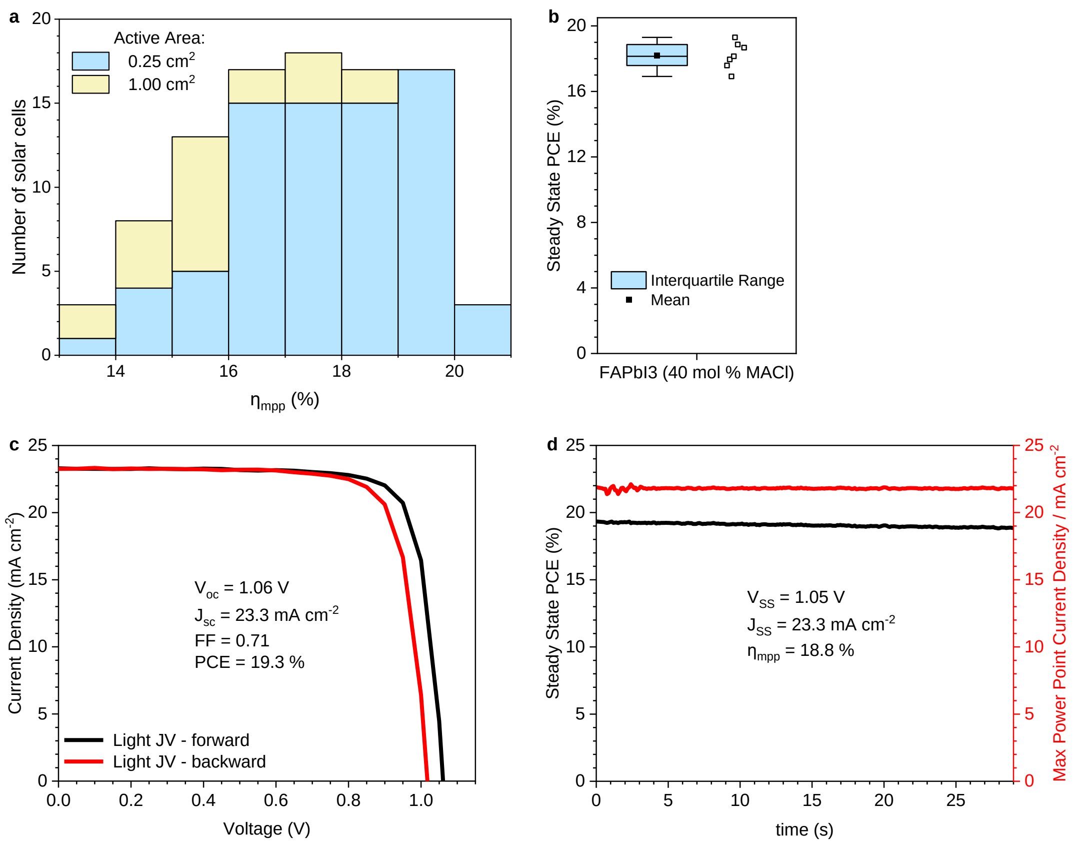

Supplementary Figure 20 | Reproducible PSC performance. a, Distribution of highest performing 2D-intermediate α-$FAPbI_3$ 0.25 cm^2 (75 cells) and 1.00 cm^2 (21 cells) solar devices fabricated *via* our optimised recipe across three representative batches during this work, containing a total of 87 small area (0.25 cm^2) and 29 large area (1.00 cm^2) cells. Devices with maximum power point tracked efficiency (η_{mpp}) below 13 % are considered not representative and thus are excluded. **b**, Distribution of a batch of control devices (silver counter electrodes employed) based on $FAPbI_3$ (40 mol % excess MACl), as described in Supplementary Note 6. PCE values in **a** and **b** are not corrected by the spectral mismatch factor (M = 1.022). J-V characteristics (**c**) and η_{mpp} and current density (J_{MPP}) measured at maximum power point (**d**) for champion devices fabricated from $FAPbI_3$ (40 mol % excess MACl) with illuminated area 0.25 cm^2.

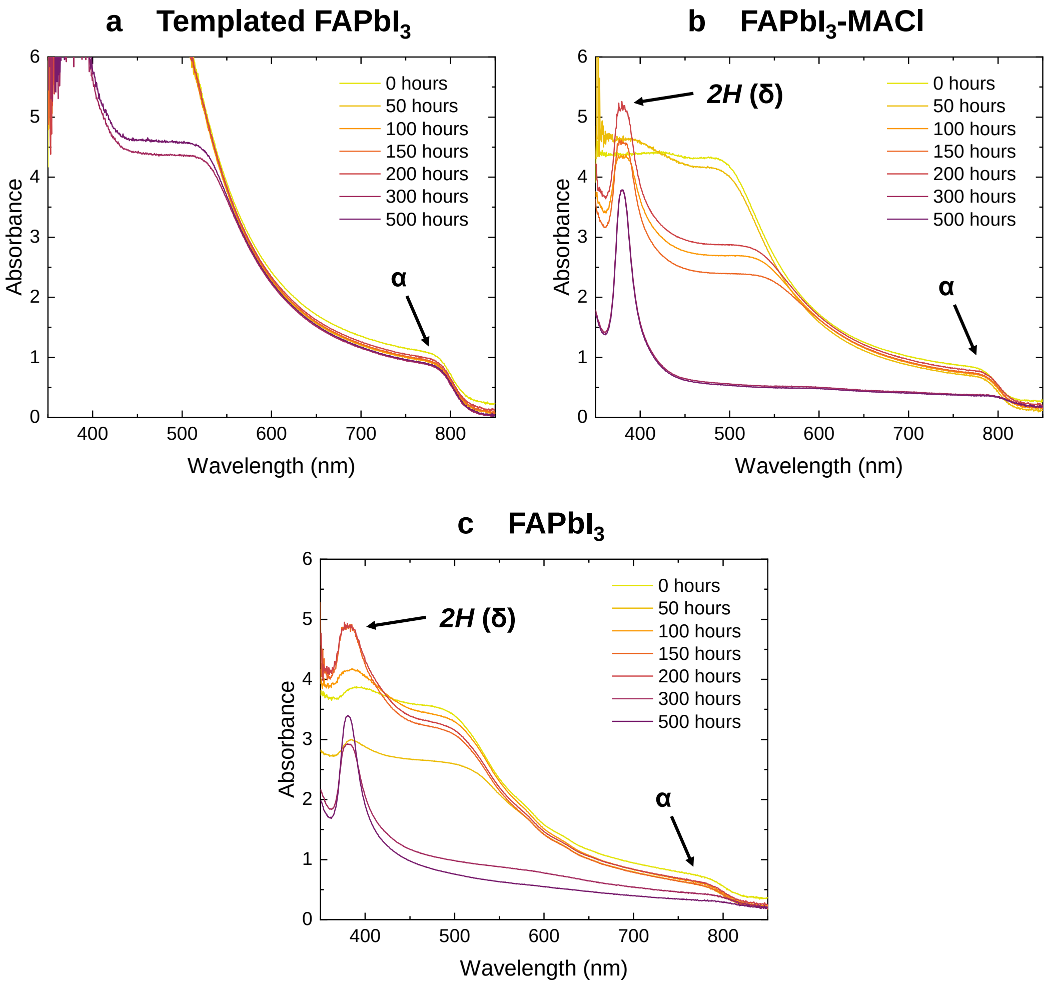

Supplementary Figure 21 | Enhanced ambient stability: Absorbance. Evolution of absorbance spectra of 2D-intermediate $FAPbI_3$ (**a**), $FAPbI_3$-MACl (**b**) and $FAPbI_3$ (**c**) over 500 hours under ambient conditions (18-22 °C, 20-55% relative humidity). The processing conditions for these three materials are described in the Methods above, and in Supplementary Note 6.

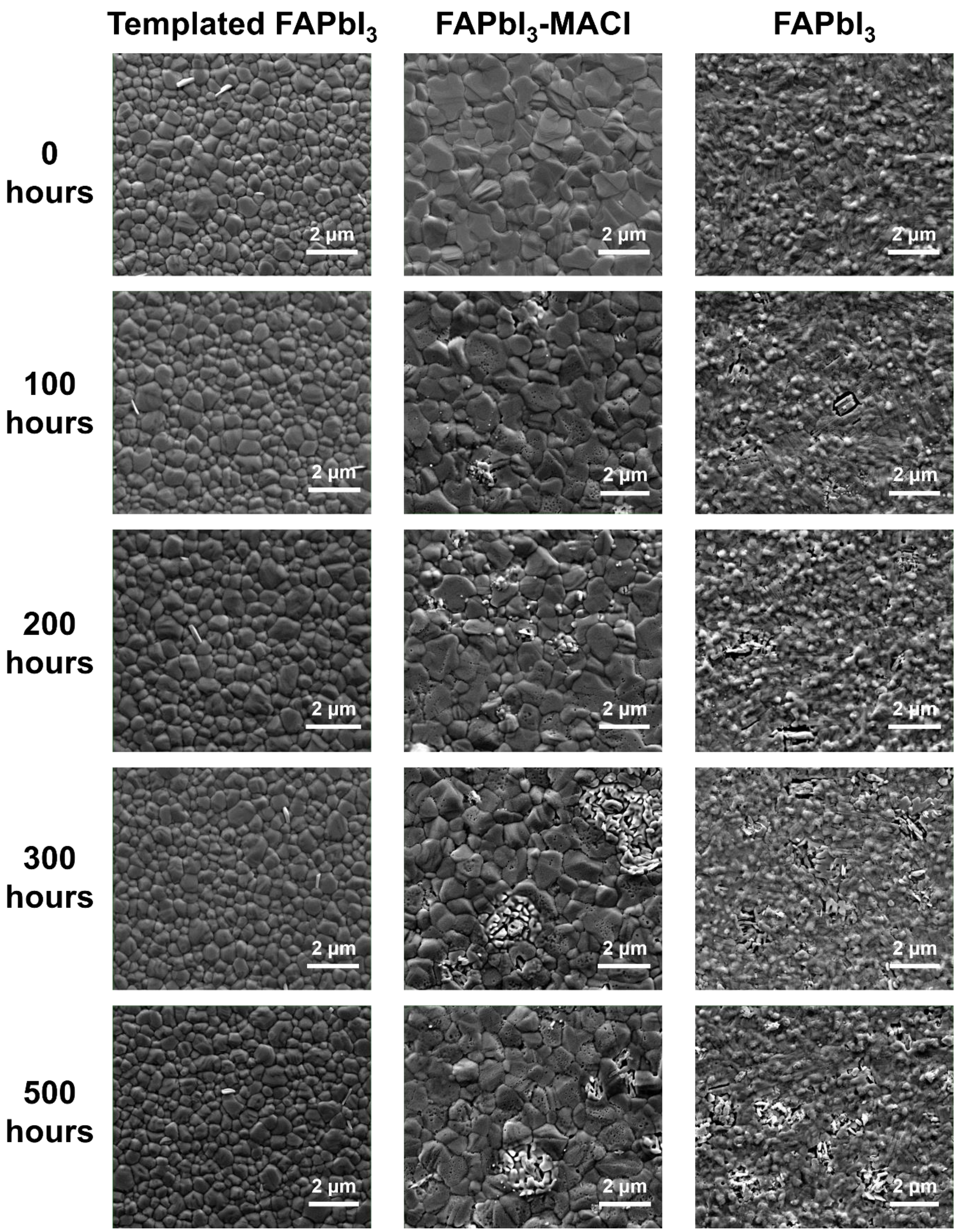

Supplementary Figure 22 | Enhanced ambient stability: Scanning electron microscopy. Evolution of microstructure of 2D-intermediate $FAPbI_3$ (**left**), $FAPbI_3$-MACl (**middle**) and $FAPbI_3$ (**right**) over 500 hours under ambient conditions (18-22 °C, 20-55% relative humidity).

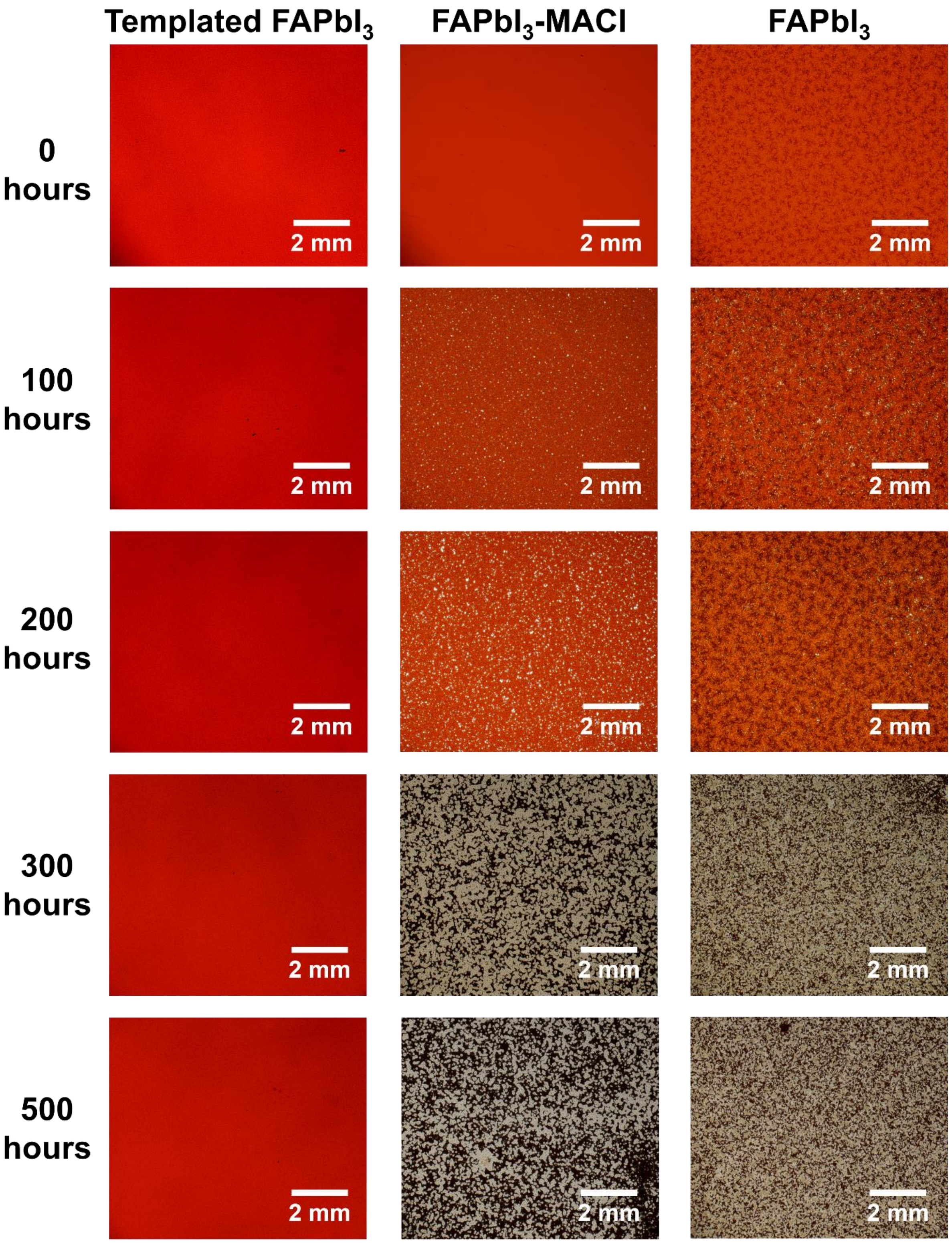

Supplementary Figure 23 | Enhanced ambient stability: Visible light microscopy. Evolution of macrostructure of 2D-intermediate $FAPbI_3$ (**left**), $FAPbI_3$-MACl (**middle**) and $FAPbI_3$ (**right**) over 500 hours under ambient conditions (18-22 °C, 20-55% relative humidity).

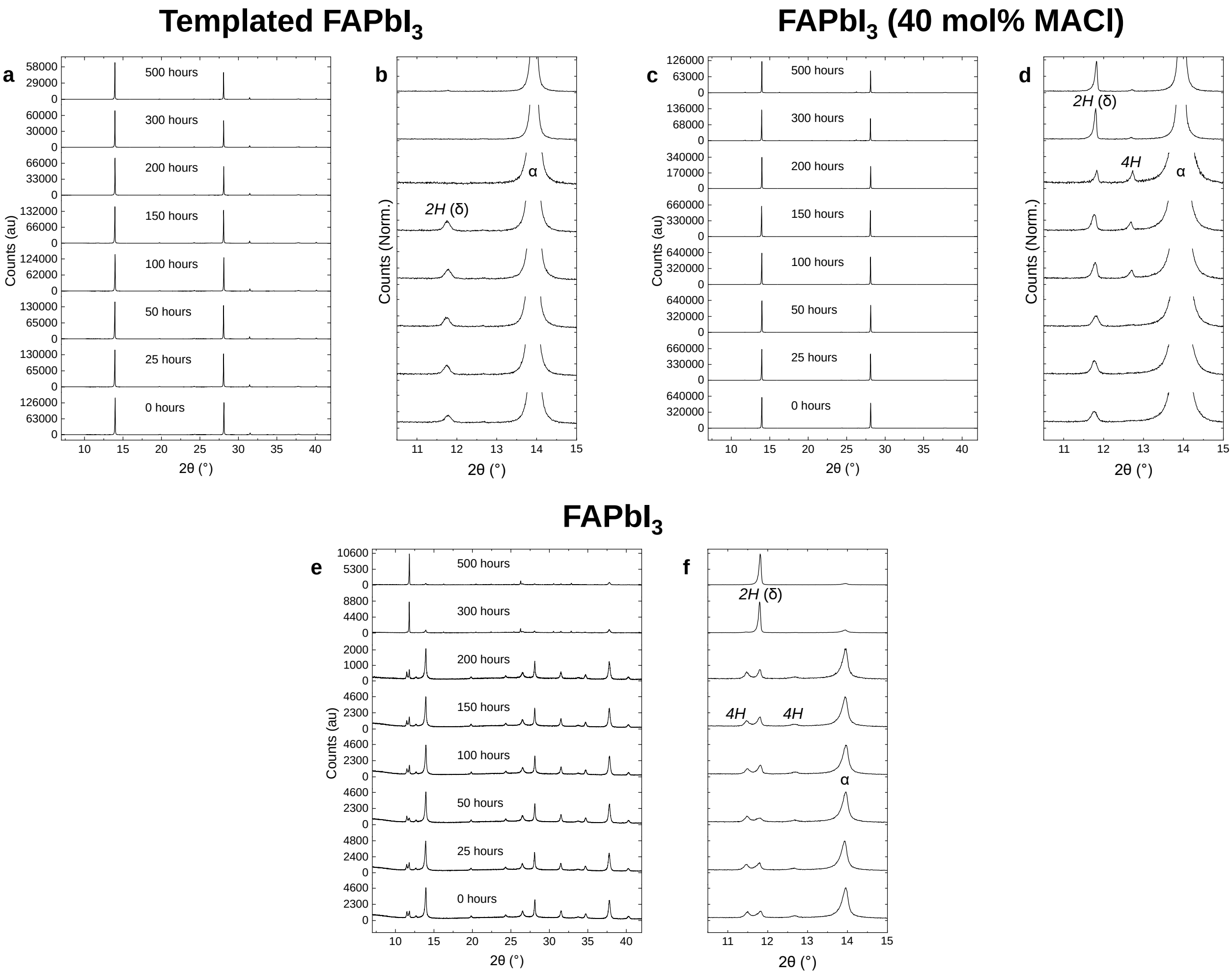

Supplementary Figure 24 | 2D-intermediate FAPbI3 ambient aging XRD. a, Evolution of 2D-intermediate $FAPbI_3$ under ambient conditions (18-22 °C, 20-55 % relative humidity). **b**, Expansion of **a** to highlight evolution of minority phases. **c**, Evolution of $FAPbI_3$-MACl under ambient conditions (18-22 °C, 20-55 % relative humidity). **d**, Expansion of **c** to highlight evolution of minority phases. **e**, Evolution of $FAPbI_3$ under ambient conditions (18-22 °C, 20-55 % relative humidity). **e**, Expansion of **f** to highlight evolution of minority phases

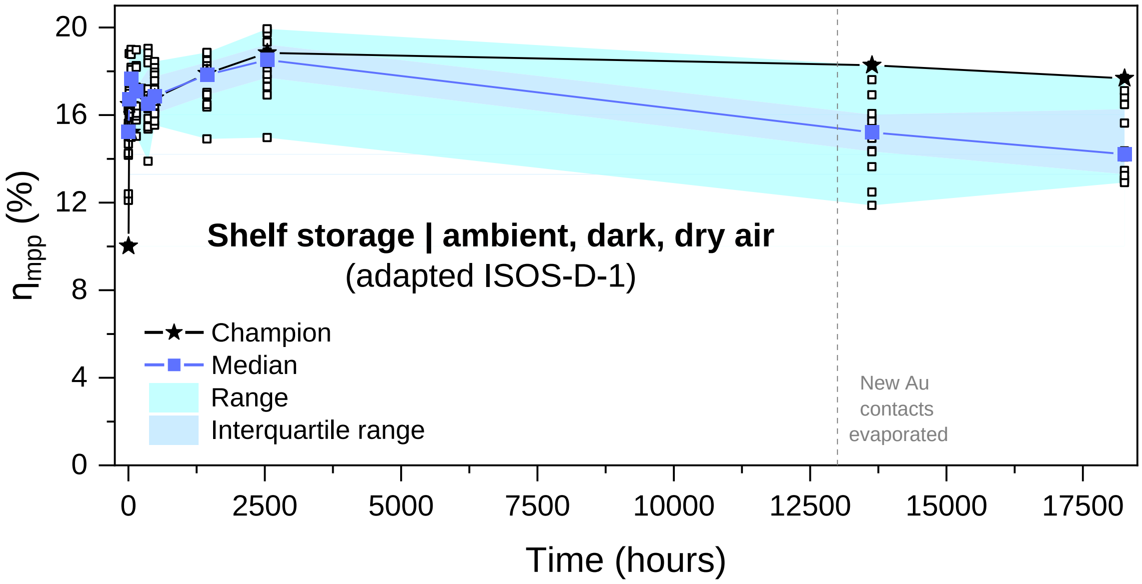

Supplementary Figure 25 | Long-term shelf stability. Unencapsulated 2D-intermediate $FAPbI_3$ n-i-p PSCs stored in dark in dry air, <10 % relative humidity, at room temperature.

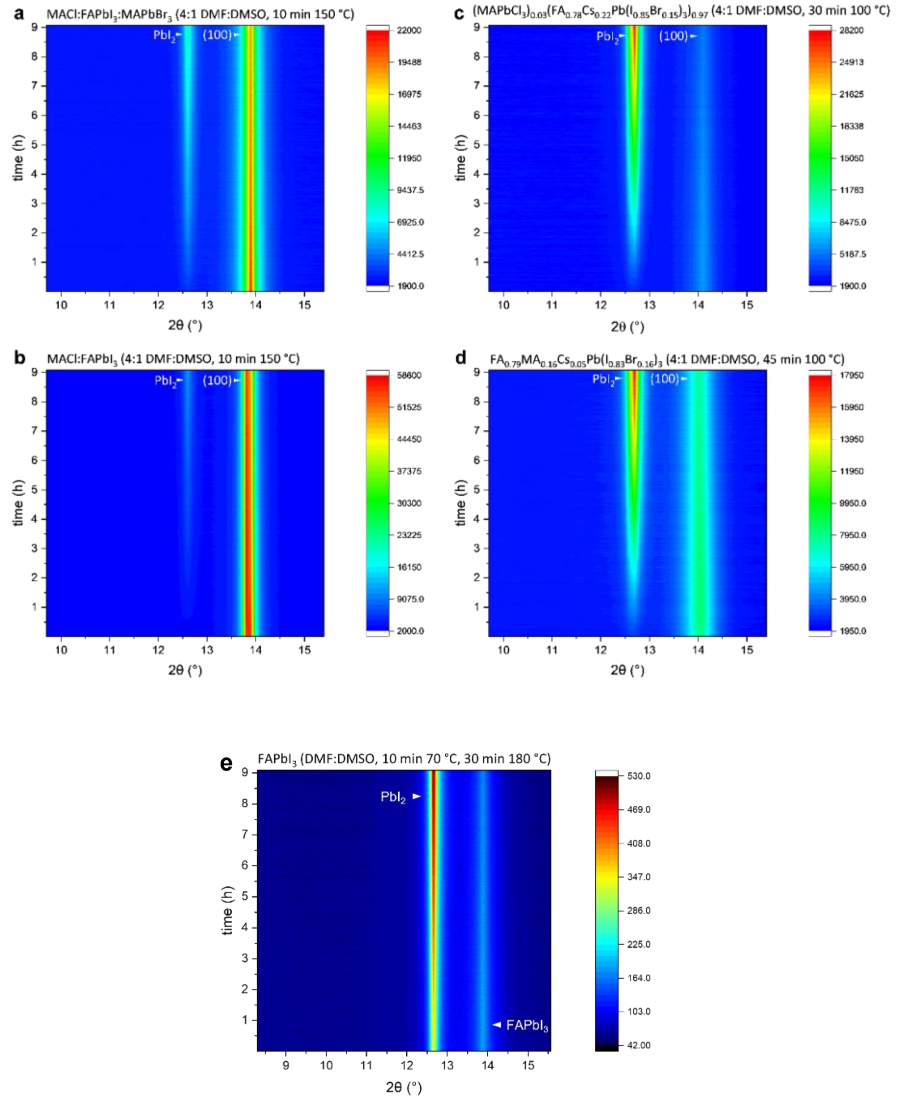

Supplementary Figure 26 | Thermal stability of state-of-the-art perovskites. Contour maps depicting in-situ x-ray diffraction (XRD) analysis of perovskite materials thermally degrading whilst held at 130 °C for 9 hours. Perovskite compositions investigated: $(FAPbI_3)_{0.95}(MAPbBr_3)_{0.95}$ with 35 mol % MACl additive, processed *via* DMF:DMSO (4:1) and quenched with diethyl ether antisolvent before thermal treatment at 150 °C for 10 minutes (**a**); $FAPbI_3$ with 35 mol % MACl additive, processed *via* DMF:DMSO (4:1) and quenched with diethyl ether antisolvent before thermal treatment at 150 °C for 10 minutes (**b**); $(MAPbCl_3)_{0.03}(FA_{0.78}Cs_{0.22}Pb(I_{0.85}Br_{0.15})_3)_{0.97}$ ("triple halide perovskite"), processed *via* DMF:DMSO (4:1) and quenched with methyl acetate antisolvent before thermal treatment at 100 °C for 30 minutes (**c**); $FA_{0.79}MA_{0.16}Cs_{0.05}Pb(I_{0.83}Br_{0.17})_3$ ("triple cation perovskite"), processed *via* DMF:DMSO (4:1) and quenched with chlorobenzene antisolvent before thermal treatment at 100 °C for 45 minutes (**d**). $FAPbI_3$ processed from DMF:DMSO (4:1), antisolvent quenched with diethyl ether and thermally treated with exactly the same procedure as for 2D-intermediate $FAPbI_3$; 70 °C for 10 minutes, followed by 180 °C for 30 minutes (**e**).

Unexpectedly, we find no clear correlation between the presence of MA^+ in a perovskite and the thermal stability of that composition at 130°C, as judged by XRD (Fig. 5a-g, Supplementary Fig. 26)[44–47]. We do not dismiss the role facile MA^+ degradation may play in perovskite

instability, but emphasise that our results indicate that the processing route appears to be the more critical factor[48].

In contrast to previous reports that have implicated two-step annealing as a source of improved thermal stability with respect to PbI_2 formation[49], we find that employing the same thermal annealing treatment on $FAPbI_3$ thin films processed conventionally from DMF:DMSO as for 2D-intermediate $FAPbI_3$ not only does not improved thermal stability, but instead results in an initial thin film material that is majority PbI_2, not $FAPbI_3$ (Supplementary Fig. 26e).

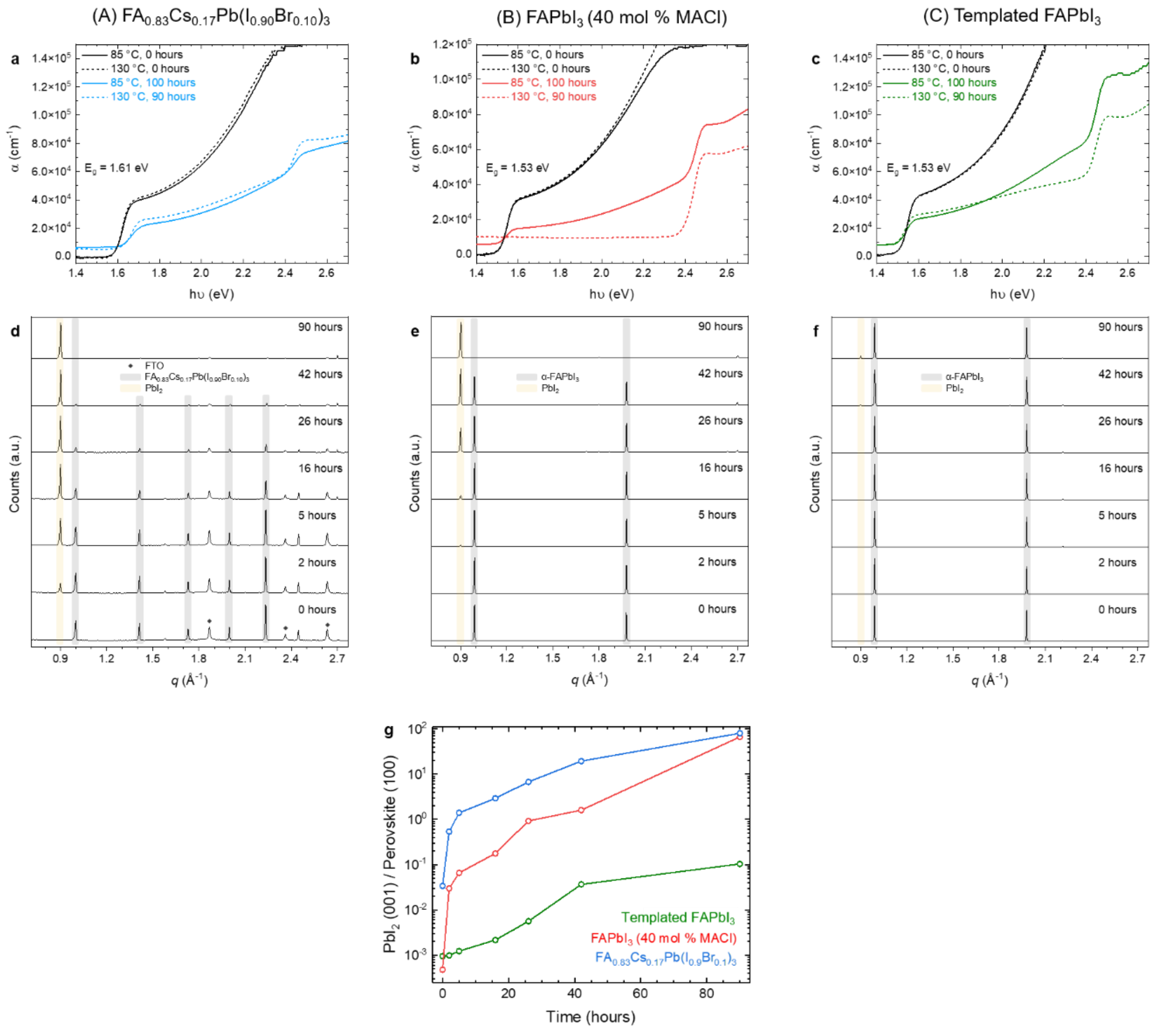

Supplementary Figure 27 | Perovskite long-term thermal stability. a-c, Absorption spectra of state-of-the-art $FA_{0.83}Cs_{0.17}Pb(I_{0.9}Br_{0.1})_3$ fabricated *via* conventional DMF:DMSO solvent mixtures (A), α-$FAPbI_3$ fabricated with excess MACl (40 mol %) *via* conventional DMF:DMSO solvent mixtures (B), and 2D-intermediate α-$FAPbI_3$ fabricated *via* our ether-amine system (C), respectively, before and after thermal treatments. **d-f**, 1D XRD patterns of A, B and C, respectively, during thermal treatment at 130 °C. All thermal treatments were carried out in air. **g,** Summary of lead (II) iodide evolution observed in **d-f**. Integrated intensity of lead (II) iodide (001) peak is tracked against time.

Supplementary Note 6

In this work a wide range of state-of-the-art perovskite materials are investigated and reported upon, notably during thermal stability testing *via* XRD and TG-GC-MS analysis. This is done to offer the reader the widest possible context in which to understand the advantages for material stability offered by the 2D-intermediate α-$FAPbI_3$ material, and the 2D-intermediate sequential approach more broadly. The overwhelming majority of materials employed, other than that novel to this work, have been developed in our laboratory on the basis of prior publications; both from within our group, and from other leading investigators in our field around the world. Here we present a comprehensive list of all materials utilised herein, an overview of our processing of them, and an exhaustive list of prior work from which we have developed these.

1. **α-$FAPbI_3$ without MACl additive (DMF:DMSO)**

   Stoichiometric FAI and PbI_2 were dissolved in a 4:1 mixture of DMF:DMSO at 1.2 M concentration. 150 μL of this solution was dispensed statically on a FTO substrate under an atmosphere of air controlled to 18-24 % relative humidity. The substrate was spun at 6,000 rpm (12,000 rpm s^{-1}) for 35 seconds. At 10 seconds into this procedure, 800 μL of diethyl ether antisolvent was dispensed slowly onto the spinning substrate. The substrate was then heated in ambient air at either 150 °C for 10 minutes (Fig. 4c, Supplementary Fig. 28b) or 180 °C for 50 minutes (Fig. 4d, Supplementary Fig. 28c). This protocol is adapted from that of Kim, et al.[50]

2. **α-$FAPbI_3$ with excess (40 mol %) MACl additive (DMF:DMSO)**

   Stoichiometric FAI and PbI_2 were dissolved in a 4:1 mixture of DMF:DMSO at 1.8 M concentration, along with a 40 mol % excess of MACl. 150 μL of this solution was dispensed statically on a FTO substrate under an atmosphere of air controlled to 18-24 % relative humidity. The substrate was spun at 6,000 rpm (12,000 rpm s^{-1}) for 35 seconds. At 10 seconds into this procedure, 800 μL of diethyl ether (or anisole,

Supplementary Fig. 28e) antisolvent was dispensed slowly onto the spinning substrate. The substrate was then heated in ambient air at 150 °C for 10 minutes (Supplementary Fig. 26b, Supplementary Fig. 28d). This protocol is adapted from that of Kim, et al.[50]

3. **α-$FAPbI_3$ *via* sequential deposition (DMF:DMSO)**

A 1.5 M solution of PbI_2 in DMF:DMSO (9:1) was prepared. 150 µL of this solution was dispensed statically on a FTO substrate under in an inert atmosphere (N_2). The substrate was spun at 1,500 rpm (300 rpm s^{-1}) for 30 seconds. The substrate was then annealed for 60 seconds at 70 °C. A further 150 uL of a solution containing FAI, MAI and MACl in a 1:0.075:0.25 stoichiometry dissolved in IPA was dispensed on the substrate before immediately spinning the substrate at 2,500 rpm (1,00 rpm s^{-1}) for 30 seconds. The substrate was then immediately removed to an atmosphere of air with relative humidity controlled to between 25-30 % and thermally cured at 150 °C for 15 minutes (Supplementary Fig. 28f)[51].

4. **$FA_{0.83}Cs_{0.17}Pb(I_{0.9}Br_{0.1})_3$ (DMF:DMSO)**

FAI (207.0 mg), PbI_2 (568.0 mg), $PbBr_2$ (80.0 mg) and CsI (64.0 mg) were dissolved in 1 mL of a 4:1 mixture of DMF:DMSO (1.23 M). 100 µL of this solution was dispensed statically on a FTO substrate under in an inert atmosphere (N_2). The substrate was spun at 2,000 rpm (1,000 rpm s^{-1}) for 10 seconds, followed by an increase to 6,000 rpm (2,000 rpm s^{-1}) for a further 35 seconds. 10 seconds before the end of this procedure, 170 µL of anisole (or diethyl ether, Supplementary Fig. 28l) antisolvent was dispensed onto the spinning substrate. The substrate was then heated in the same environment at either 100 °C for 15 minutes (Fig. 4e, Supplementary Fig. 28i), 150 °C for 10 minutes (Supplementary Fig. 28i, j) or 180 °C for 50 minutes (Fig. 4f,

Supplementary Fig. 28k). This protocol is adapted from one in common use within our group, for example as described by McMeekin, et al[52].

5. $(FAPbI_3)_{0.95}(MAPbBr_3)_{0.05}$ with excess (35 mol %) MACl additive (DMF:DMSO)

Stoichiometric FAI and PbI_2 were dissolved in a 19:1 ratio with stoichiometric MABr and $PbBr_2$, in a 8:1 mixture of DMF:DMSO at 1.8 M concentration, along with a 35 mol % excess of MACl. 150 µL of this solution was dispensed statically on a FTO substrate under an atmosphere of air controlled to 18-24 % relative humidity. The substrate was spun at 6,000 rpm (12,000 rpm s^{-1}) for 35 seconds. At 10 seconds into this procedure, 800 µL of diethyl ether antisolvent was dispensed slowly onto the spinning substrate. The substrate was then heated in ambient air at 150 °C for 10 minutes (Supplementary Fig. 26a). This protocol is adapted from that of Min, et al.[53]

6. $(MAPbCl_3)_{0.03}(FA_{0.78}Cs_{0.22}Pb(I_{0.85}Br_{0.15})_3)_{0.97}$ ("triple halide perovskite") (DMF:DMSO)

FAI, CsI, PbI_2 and $PbBr_2$ were mixed in stoichiometric ratio 0.78 : 0.22 : 0.85 : 0.15. To these, $PbCl_2$ and MACl (1:1 by mol) powders were added such that they comprise 3 mol % of the total precursor solids. These precursors were dissolved in a 3:1 mixture of DMF:DMSO. 100 µL of this solution was spread statically on a FTO substrate under in an inert atmosphere (N_2). The substrate was spun at 5,000 rpm (2,500 rpm s^{-1}) for 50 seconds. Between 25-30 seconds from the beginning of this procedure, 200 µL of methyl acetate antisolvent was dispensed onto the spinning substrate. The substrate was then heated in the same environment at 100 °C for 30 minutes (Supplementary Fig. 26c). This protocol is adapted from that of Xu, et al.[54]

7. $FA_{0.79}MA_{0.16}Cs_{0.05}Pb(I_{0.83}Br_{0.17})_3$ ("triple cation perovskite") (DMF:DMSO)

A precursor solution containing FAI, PbI_2, MABr and $PbBr_2$ in a 1:1.1:0.2:0.2 stoichiometry at 1.1 M (with respect to Pb^{2+}) concentration was prepared using a DMF:DMSO (4:1) solvent mixture. A 1.5 M solution of CsI in DMSO was prepared separately and added to the precursor solution such that the desired composition was achieved. 100 µL of the precursor solution was then dispensed statically on a FTO substrate under in an inert atmosphere (N_2). The substrate was spun at 1,000 rpm (1,000 rpm s^{-1}) for 10 seconds, followed by an increase to 6,000 rpm (2,000 rpm s^{-1}) for a further 20 seconds. 5 seconds before the end of this procedure, 100 µL of chlorobenzene antisolvent was dispensed onto the spinning substrate. The substrate was then heated in the same environment at 100 °C for 60 minutes.[55]

8. $MAPbI_3$ (DMF:DMSO)

A 1.3 M solution of PbI_2 and MAI in 1:1 stoichiometry was prepared using a 4:1 v/v DMF:DMSO solvent mixture. 100 µL of the precursor solution was then dispensed statically on a FTO substrate under in an inert atmosphere (N_2). The substrate was spun at 1,000 rpm (1,000 rpm s^{-1}) for 10 seconds, followed by an increase to 6,000 rpm (2,000 rpm s^{-1}) for a further 30 seconds. 20 seconds before the end of this procedure, 150 µL of chlorobenzene antisolvent was dispensed onto the spinning substrate. The substrate was then heated in the same environment at 100 °C for 45 minutes.[56]

9. $MAPbI_3$ (ACN)

MAI and PbI_2 were mixed in a 1:1.06 stoichiometry and ACN added such that the concentration was 0.5 M with respect to Pb (II). A black suspension is formed within a few seconds of mixing. Methylamine gas (40 wt. % in ethanol) is bubbled through this solution by means of a carrier gas (N_2) until the solution was fully dissolved. 100 µL of this solution was dispensed dynamically on a FTO substrate (2,000 rpm) under an

atmosphere of dry air (<5 % relative humidity). The substrate was then heated in the same environment at 100 °C for 45 minutes (Fig. 4h, Supplementary Fig. 25o). This protocol is adapted from that of Noel, et al.[57]

Supplementary Note 7

To investigate the stability of our α-$FAPbI_3$ material under operational conditions, and further explore the relationship between perovskite processing route and resultant stability, we subject a range of perovskite thin film materials to a variety of environmental stressing conditions. We first perform in-situ XRD measurements at 130 °C in ambient air (~30% RH) to track the thermal degradation of a selection of lead halide perovskite compositions, processed *via* a range of different methods (Fig. 5a-g and Supplementary Fig. 26). Full processing methodology of all perovskite materials investigated is given in Supplementary Note 6. For most of the perovskite films, the main features identified during degradation are a reduction in the scattering intensity of the perovskite phase over time, and a corresponding increase in the PbI_2 scattering intensity, consistent with thermal decomposition of FA^+-rich perovskites into PbI_2.

The most striking result of these measurements is the greatly reduced rate of crystalline PbI_2 formation under thermal aging of both 2D precursor phase-engineered α-$FAPbI_3$ processed *via* MeTHF (Fig. 5g) and surprisingly $MAPbI_3$ processed *via* ACN (Fig. 5f), in comparison with the same compositions fabricated *via* DMF:DMSO solvent systems. Moreover, as the scattering intensity of the 2D precursor phase-engineered α-$FAPbI_3$ peaks reduce only minimally during aging we infer that other thermal degradation routes of the perovskite phase (besides decomposition to PbI_2) are also not active. Remarkably, even after thermal aging at 130 °C for 90 hours in air, we find that our α-$FAPbI_3$ degrades only minimally to PbI_2 while α-$FAPbI_3$ from DMF:DMSO is completely degraded (Supplementary Fig. 27). Notably, the long-term thermal stability of perovskites processed from DMF:DMSO is improved when higher curing temperatures are employed during initial perovskite processing (Fig. 5a-d). However, it is also evident that more PbI_2 is already present in the as-processed materials cured at a higher temperature. Increased full-width half-maxima of initial perovskite XRD reflections are also consistent with reduced crystallinity in the materials, which may correspond to a greater defect density present already in the as-processed material. We find that this is also true of

$FAPbI_3$ processed from DMF:DMSO and treated with the same two-step annealing strategy as 2D precursor phase-engineered $FAPbI_3$ (Supplementary Fig. 26). Notably, despite the same high temperature processing, our α-$FAPbI_3$ is not compromised in either of these ways. Unexpectedly, we find no clear correlation between the presence of MA^+ in a perovskite and the thermal stability of that composition at 130°C, as judged by XRD (Fig. 5a-g, Supplementary Fig. 26)[62–65]. We do not dismiss the role facile MA^+ degradation may play in perovskite instability, but emphasise that our results indicate the processing route appears to be a more critical distinguishing factor[66]. A further key result is that 2D precursor phase-engineered α-$FAPbI_3$ films have a much higher thermal stability under these conditions than $FA_{0.83}Cs_{0.17}Pb(I_{0.9}Br_{0.1})_3$ films, which are processed from DMF:DMSO and have been established as a highly stable perovskite composition[23,24].

To further probe the degradation pathways, we employ thermal desorption-gas chromatography-mass spectrometry (TD-GCMS). This technique desorbs volatile organic components (VOCs) from the perovskite material (400-900 nm thick layers) before separating and characterising each species individually. These may be volatile components retained during processing, or degradation products of the perovskite material. TD-GCMS is extremely sensitive. Based on previous reports on the retention of DMSO in gelatine media[67], we calculate that the detection limit of this chemical within a 500 nm thick layer of perovskite material coated on a 25 cm^2 substrate is 0.002 wt.%, assuming 50% desorption. The molecular structures shown in Fig. 5a-g indicate the VOCs detected *via* TD-GCMS for each perovskite composition investigated. By combining our *in-situ* XRD and TD-GCMS measurements we can observe not only the products of degradation remaining in the solid-state, but many of those that are volatilised concurrently. As a result, we determine two key factors that cumulatively explain the enhanced ambient and thermal stability of our 2D precursor phase-engineered α-$FAPbI_3$ (further discussion of our TD-GCMS results is given in Supplementary Note 8).

First, our $FAPbI_3$ is unique among all FA^+-containing perovskites investigated (from 15 different perovskites, Supplementary Fig. 28) in not evolving *sym*-triazine during TD-GCMS analysis (a total of seven individual 2D precursor phase-engineered $FAPbI_3$ samples were analysed, with no *sym*-triazine detected). *Sym*-triazine has been reported previously as a thermal degradation product of FA^+ [68–70], and thus we interpret evolution of this molecule as corresponding with FA^+ volatilisation from the perovskite thin film as FA gas and hydrogen iodide (discussed in Supplementary Note 8). α-$FAPbI_3$ perovskites fabricated *via* DMF and DMSO and cured under increasing temperatures (up to 50 minutes at 180 °C) (Supplementary Fig. 28a-e) all show the production of *sym*-triazine, ruling out perovskite composition or enhanced thermal curing as the cause of enhanced FA^+ volatilisation. Similarly, fabrication *via* a conventional sequential deposition approach, and even deposition *via* thermal evaporation (solvent free), fails to obviate *sym*-triazine evolution in the resultant α-$FAPbI_3$ material (Supplementary Fig. 28f-g). This implies that the 2D precursor phase-engineered crystallisation process itself is responsible for the suppression of FA^+ volatilisation. These results are summarised schematically in Fig. 5h and correlate with the enhanced thermal stability of our 2D precursor phase-engineered $FAPbI_3$ observed in the XRD measurements (Fig. 5g). To further determine if the suppression effect is either kinetic (the rate of FA^+ volatilisation is reduced) or energetic (the energy required to affect FA^+ volatilisation is increased), we carry out thermal gravimetric analysis-mass spectrometry (TGA-MS) measurements on a selection of FA^+-rich perovskite thin films (Supplementary Fig. 29). In contrast to our XRD or TD-GCMS experiments where samples are thermally degraded at a specified temperature for a period of hours, the TGA-MS measurement rapidly increases the temperature that the material is exposed to (5 °C min^{-1}) leading to detection of a sharp decrease in mass when volatile species are lost. We find that for all three iodide-rich perovskites the temperature of *sym*-triazine formation is consistent at around 310 °C. This suggests that the mechanism by which FA^+ volatilisation is suppressed in 2D precursor phase $FAPbI_3$ is kinetic, and that the rate of formation is so low at the temperature of our thermal

aging (130 °C) that it is undetectable even by TD-GCMS, consistent with the degradation rate observed in Fig. 5g.

Secondly, during TD-GCMS we also detect solvents used during processing in many of the perovskite materials investigated (Supplementary Fig. 28). Where solvent evolution is observed, this correlates closely with increased rate of thermal degradation (Fig. 5a-h and Supplementary Fig. 26). These results implicate the gradual release of low volatility residual processing solvents (DMSO, DMF) and antisolvents (anisole) from perovskite materials as an underlying source of thermal instability in many solution-processed perovskite materials. Harsher thermal curing conditions during fabrication might be expected to remove such residual solvents more thoroughly. Therefore, we varied the curing temperature employed during fabrication of “highly stable”, state-of-the-art $FA_{0.83}Cs_{0.17}Pb(I_{0.9}Br_{0.1})_3$ from 100 to 180 °C and found by TD-GCMS analysis that only 180 °C curing for 50 minutes was sufficient to fully remove all residual solvents (Supplementary Fig. 28h-n). As expected, higher temperature curing also led to improved thermal stability (Fig. 5b,d). Notably, no such VOCs were evolved from perovskites fabricated *via* high volatility solvents (MeTHF, ACN) (Supplementary Fig. 28a, o), directly correlating to the improved thermal stability of these materials (Fig. 5f-g). To our knowledge, a report in 2021 from Chen, et al., is the first to link residual solvents with material instability[14]. The authors observed void-formation at the underlying perovskite interface over time and inferred from this the gradual loss of DMSO entrapped during processing. Such kinetic entrapment of low-volatility solvents at the point of crystallisation has been shown to lead to solvent inclusions in the perovskite material[71]. We speculate that strongly Pb^{2+}-coordinating solvents in such inclusions may serve as pseudo-halides substituting Pb^{2+}-coordinated halide ions in the 3D haloplumbate structure, requiring corresponding A-site vacancies to ensure local charge neutrality. All such defects are undetectable *via* XRD due to their irregular nature, but could be revealed as PbI_2 upon subsequent solvent volatilisation followed by Ostwald ripening to form larger regions of crystalline PbI_2, as observed here.

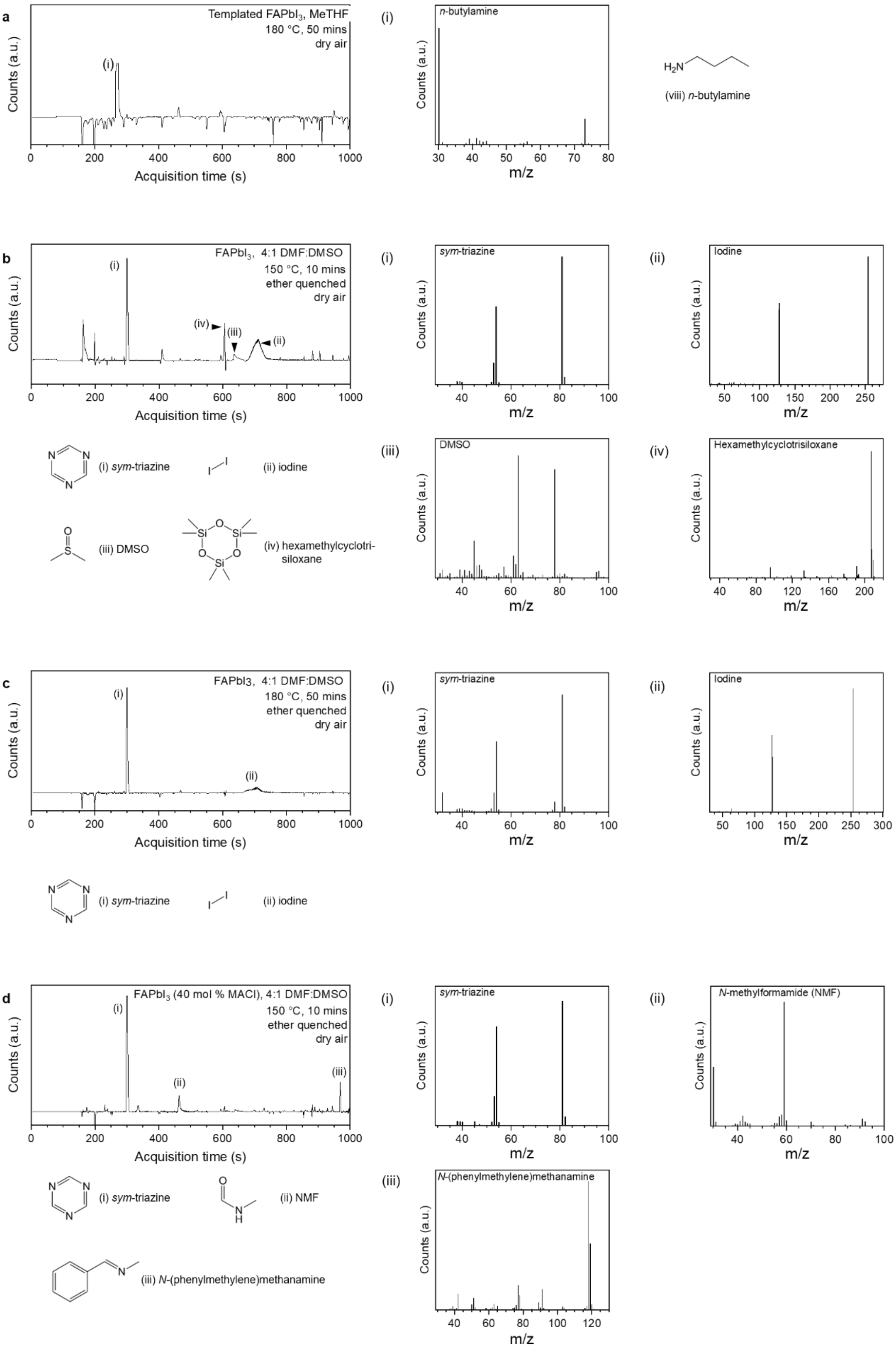
a
Templated FAPbI3, MeTHF
180 °C, 50 mins
dry air
(i)
Counts (a.u.)
Acquisition time (s)
(i)
n-butylamine
m/z
(viii) n-butylamine
b
FAPbI3, 4:1 DMF:DMSO
150 °C, 10 mins
ether quenched
dry air
(i)
(ii)
(iii)
(iv)
sym-triazine
Iodine
DMSO
Hexamethylcyclotrisiloxane
(i) sym-triazine
(ii) iodine
(iii) DMSO
(iv) hexamethylcyclotri-siloxane
c
FAPbI3, 4:1 DMF:DMSO
180 °C, 50 mins
ether quenched
dry air
d
FAPbI3 (40 mol % MACl), 4:1 DMF:DMSO
150 °C, 10 mins
ether quenched
dry air
N-methylformamide (NMF)
N-(phenylmethylene)methanamine
(ii) NMF
(iii) N-(phenylmethylene)methanamine

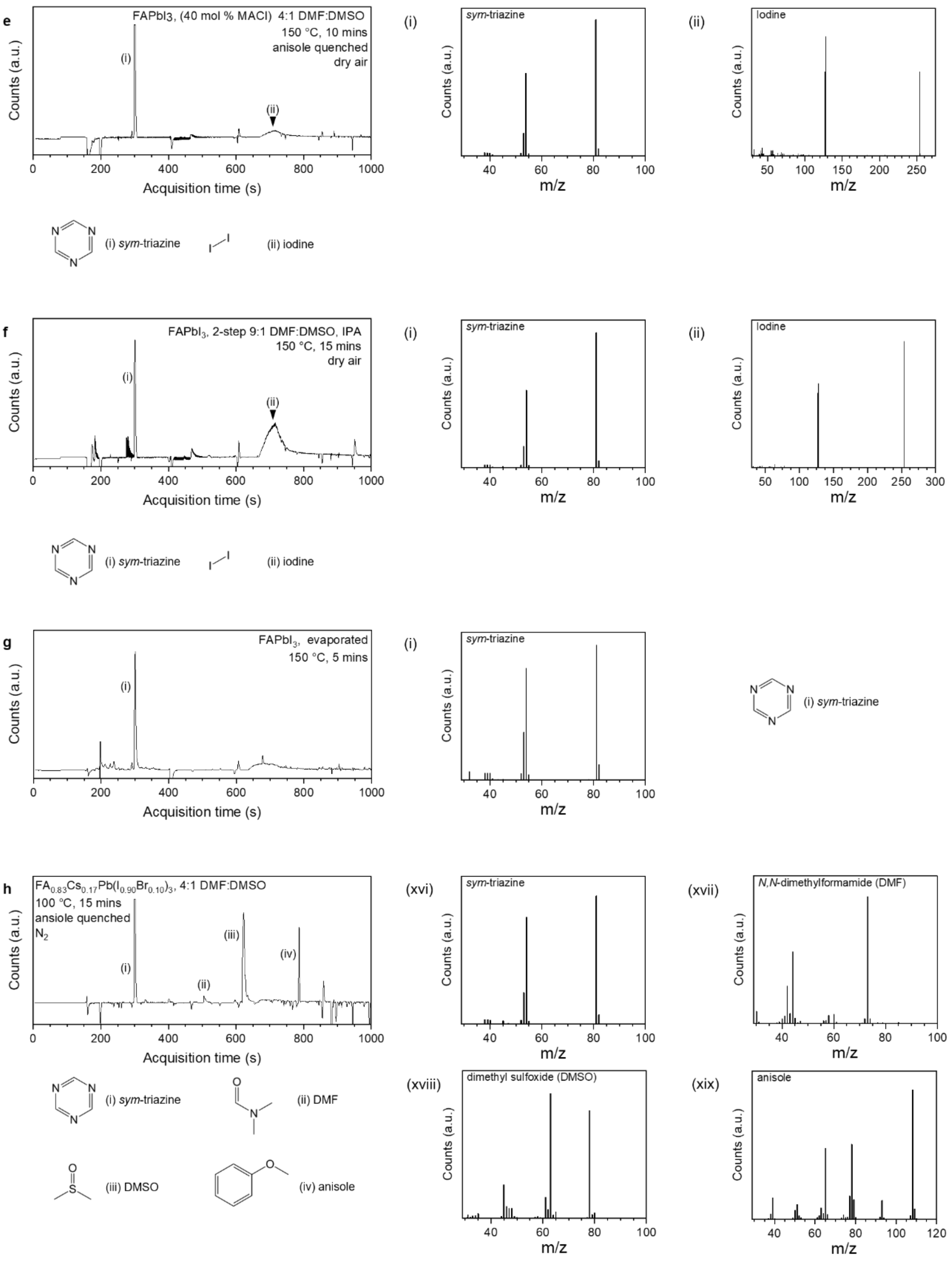

e
FAPbI3, (40 mol % MACl) 4:1 DMF:DMSO
150 °C, 10 mins
anisole quenched
dry air
(i)
(ii)
Counts (a.u.)
Acquisition time (s)
sym-triazine
Iodine
m/z
(i) sym-triazine
(ii) iodine
f
FAPbI3, 2-step 9:1 DMF:DMSO, IPA
150 °C, 15 mins
dry air
(i)
(ii)
Counts (a.u.)
Acquisition time (s)
sym-triazine
Iodine
m/z
(i) sym-triazine
(ii) iodine
g
FAPbI3, evaporated
150 °C, 5 mins
(i)
Counts (a.u.)
Acquisition time (s)
sym-triazine
m/z
(i) sym-triazine
h
FA0.83Cs0.17Pb(I0.90Br0.10)3, 4:1 DMF:DMSO
100 °C, 15 mins
ansiole quenched
N2
(i)
(ii)
(iii)
(iv)
Counts (a.u.)
Acquisition time (s)
(xvi)
sym-triazine
(xvii)
N,N-dimethylformamide (DMF)
(xviii)
dimethyl sulfoxide (DMSO)
(xix)
anisole
m/z
(i) sym-triazine
(ii) DMF
(iii) DMSO
(iv) anisole

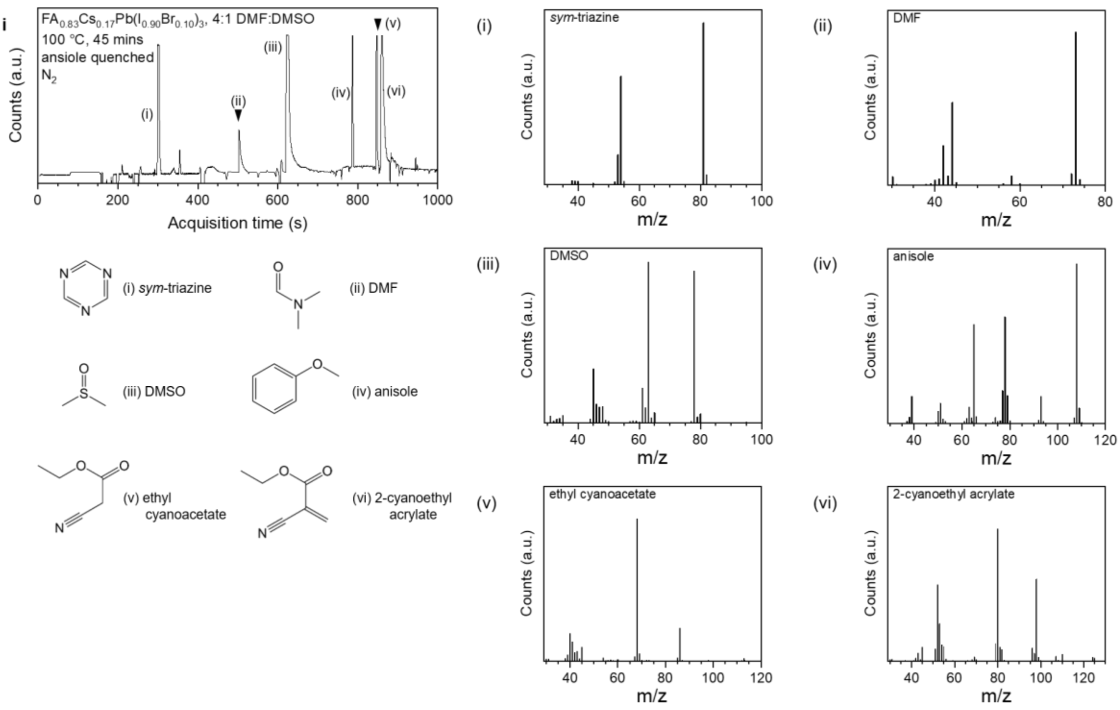
i
FA0.83Cs0.17Pb(I0.90Br0.10)3, 4:1 DMF:DMSO
100 °C, 45 mins
ansiole quenched
N2
Counts (a.u.)
Acquisition time (s)
(i) sym-triazine
(ii) DMF
(iii) DMSO
(iv) anisole
(v) ethyl cyanoacetate
(vi) 2-cyanoethyl acrylate
sym-triazine
DMF
DMSO
anisole
ethyl cyanoacetate
2-cyanoethyl acrylate
m/z

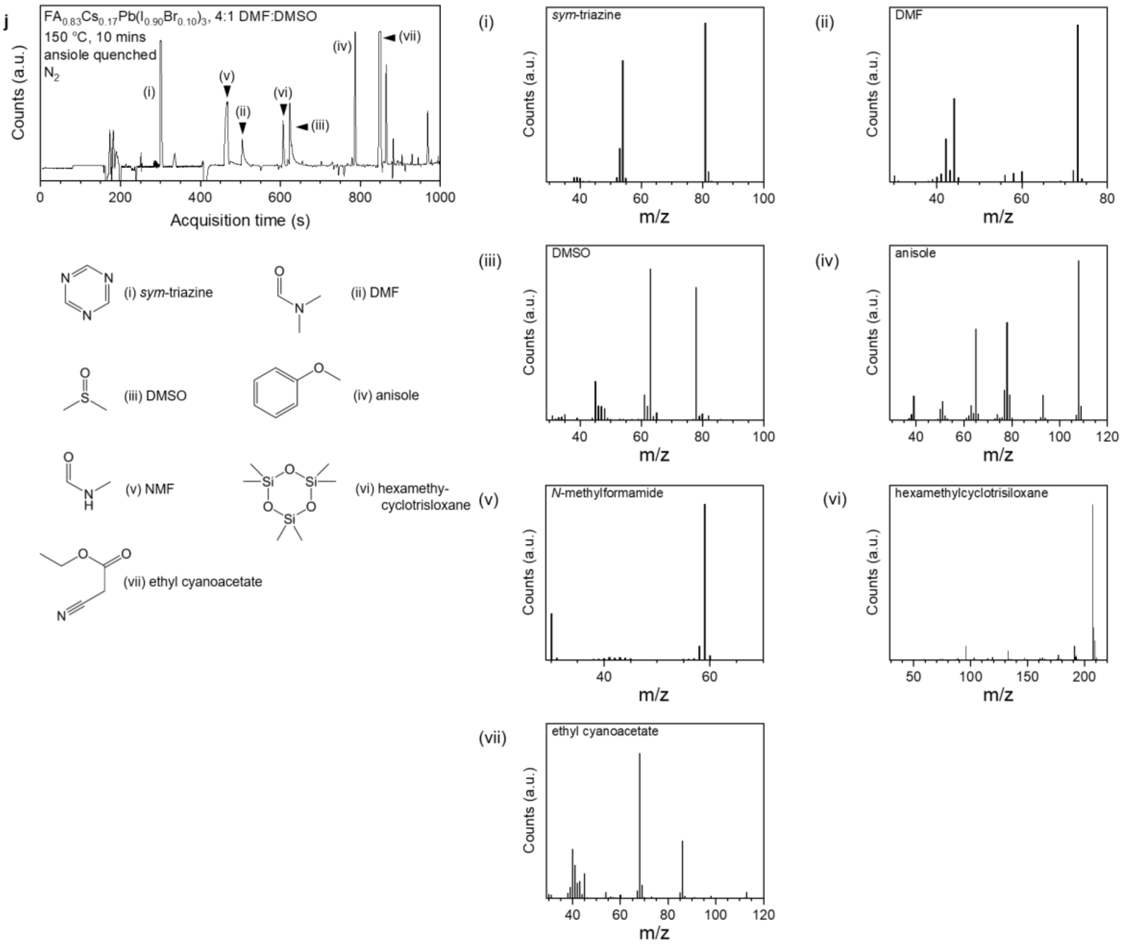
j
FA0.83Cs0.17Pb(I0.90Br0.10)3, 4:1 DMF:DMSO
150 °C, 10 mins
ansiole quenched
N2
Counts (a.u.)
Acquisition time (s)
(i) sym-triazine
(ii) DMF
(iii) DMSO
(iv) anisole
(v) NMF
(vi) hexamethy-cyclotrisiloxane
(vii) ethyl cyanoacetate
sym-triazine
DMF
DMSO
anisole
N-methylformamide
hexamethylcyclotrisiloxane
ethyl cyanoacetate
m/z

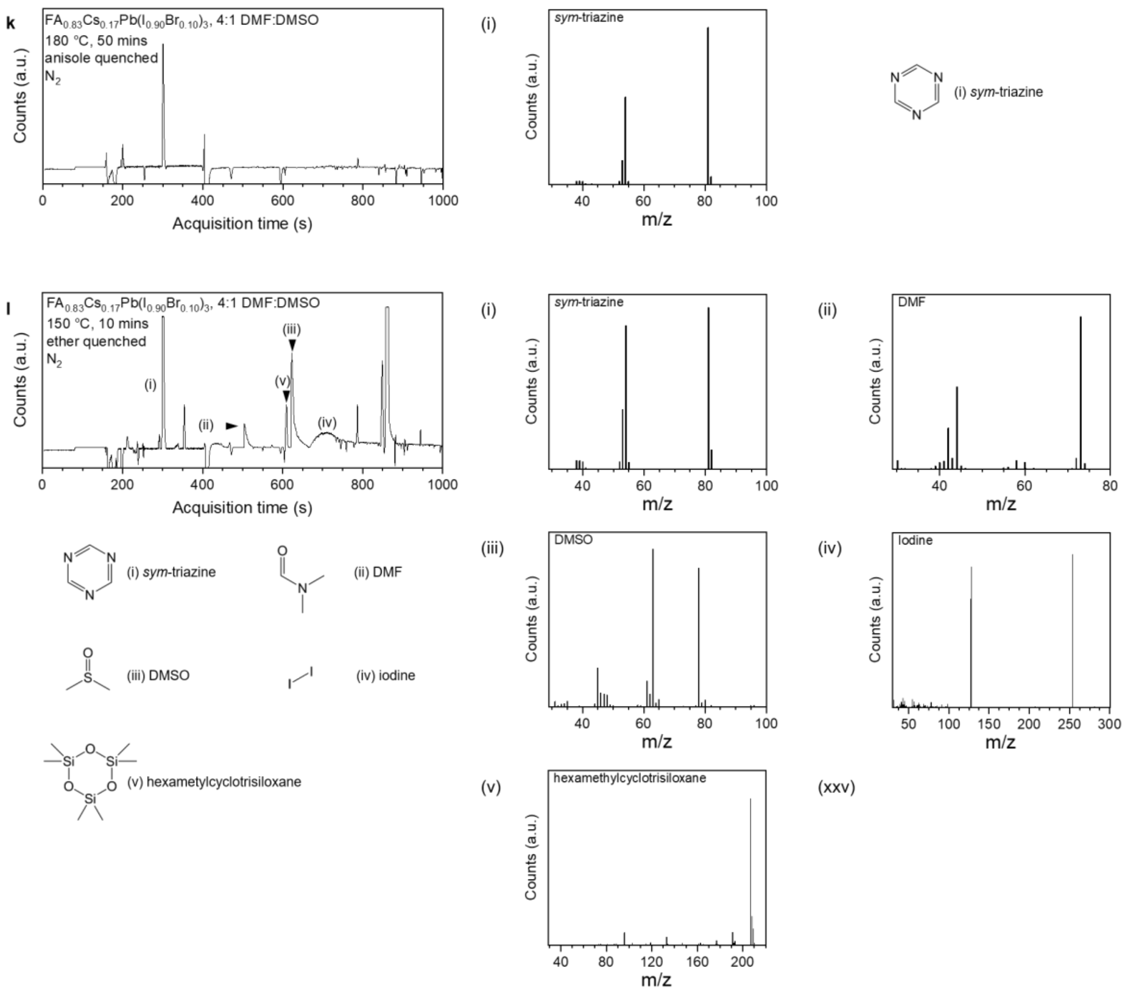
k
FA0.83Cs0.17Pb(I0.90Br0.10)3, 4:1 DMF:DMSO
180 °C, 50 mins
anisole quenched
N2
Counts (a.u.)
Acquisition time (s)
(i)
sym-triazine
m/z
(i) sym-triazine
l
FA0.83Cs0.17Pb(I0.90Br0.10)3, 4:1 DMF:DMSO
150 °C, 10 mins
ether quenched
N2
(i)
(ii)
(iii)
(iv)
(v)
DMF
DMSO
Iodine
hexamethylcyclotrisiloxane
(ii) DMF
(iii) DMSO
(iv) iodine
(v) hexametylcyclotrisiloxane
(xxv)

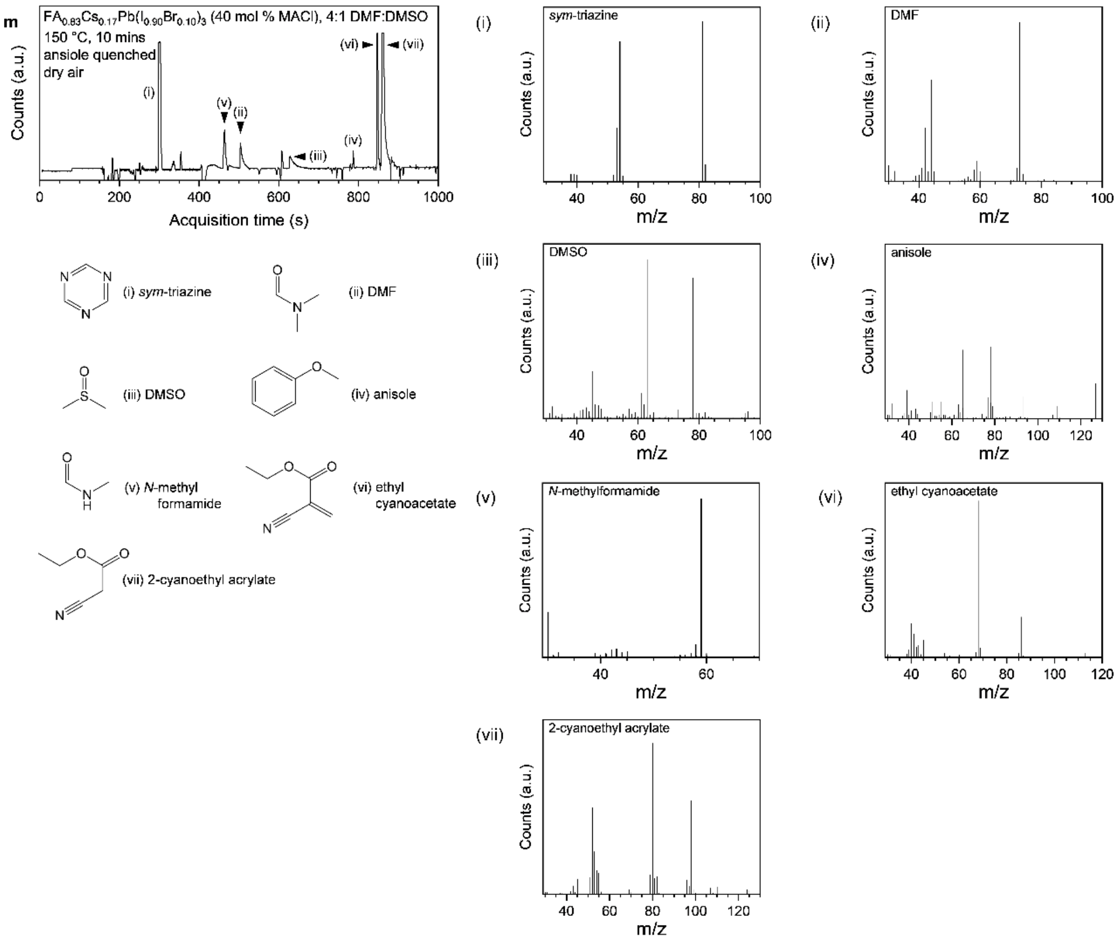
m
FA0.83Cs0.17Pb(I0.90Br0.10)3 (40 mol % MACl), 4:1 DMF:DMSO
150 °C, 10 mins
ansiole quenched
dry air
Counts (a.u.)
Acquisition time (s)
(i) sym-triazine
(ii) DMF
(iii) DMSO
(iv) anisole
(v) N-methyl formamide
(vi) ethyl cyanoacetate
(vii) 2-cyanoethyl acrylate
sym-triazine
DMF
DMSO
anisole
N-methylformamide
ethyl cyanoacetate
2-cyanoethyl acrylate
m/z

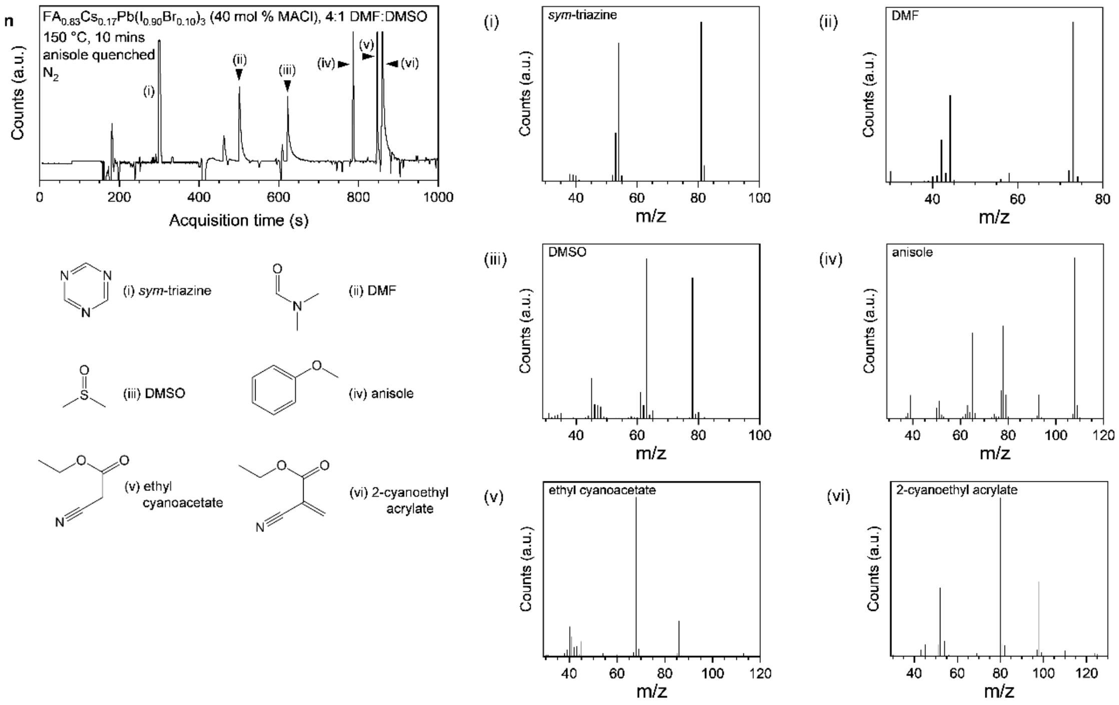
n
FA0.83Cs0.17Pb(I0.90Br0.10)3 (40 mol % MACl), 4:1 DMF:DMSO
150 °C, 10 mins
anisole quenched
N2
Counts (a.u.)
Acquisition time (s)
(i) sym-triazine
(ii) DMF
(iii) DMSO
(iv) anisole
(v) ethyl cyanoacetate
(vi) 2-cyanoethyl acrylate
sym-triazine
DMF
DMSO
anisole
ethyl cyanoacetate
2-cyanoethyl acrylate
m/z

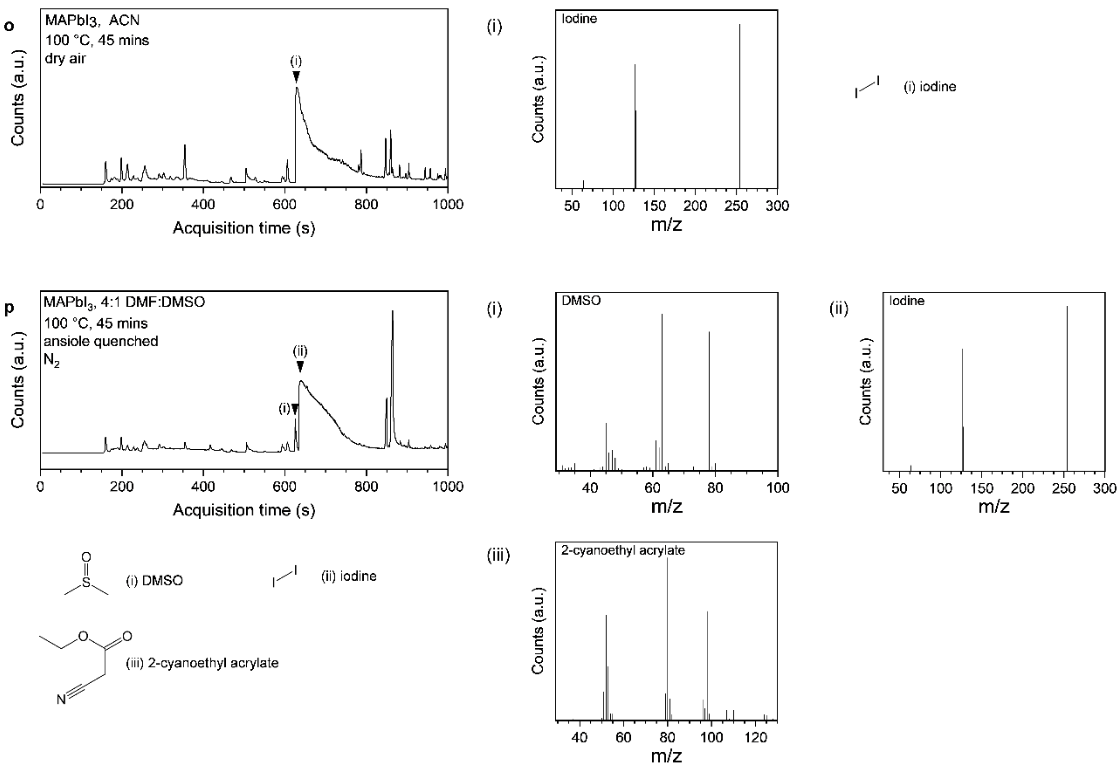

Supplementary Figure 28 | Volatile products of perovskite thermal degradation. Thermal desorption-gas chromatography-mass spectrometry (TD-GCMS) measurements. Chromatograms corresponding to thermal desorption from a wide range of state-of-the-art perovskite materials. Mass spectra of identifiable desorption products. All species are identified using the national institute of standards and technology (NIST) 17 database and show an identification score of at least ≥85 %. Unlabelled signals are those for which no satisfactory match was found in the NIST 17 database. In each case a thin layer of material (400-1000 nm thick) is thermally treated (see methods sections for details) and the volatile organic compounds (VOCs) released are collected onto a porous adsorbent. For each experiment, tubes - containing the adsorbent charged with the VOCs released from a particular perovskite - are heated rapidly to desorb the VOCs, which are then directly loaded into a gas chromatography-mass spectrometry separation-detection unit. For each perovskite composition, the experiment was carried out at least in duplicate. Supplementary Note 6 summarises the fabrication methods for the perovskite materials employed. A number of common contaminants were detected, notably hexamethylcyclotrisiloxane, *N*-(phenylmethylene)methanamine, ethyl cyanoacetate and 2-cyanoethyl acrylate. These are all associated with plastics and plastic fabrications, and as such we suspect they are introduced in trace quantities by the contact between the sharp-edged glass substrates and the plastic holding vessels in which samples were transported prior to measurements. The perovskite composition, thermal annealing conditions (temperature and duration), antisolvent quenching solvent (where relevant) and the environment in which the thin films were processed are all shown on the panels **a-p**. Roman numerals (i-vii) are used to identify the VOCs detected and positively identified during TD-GCMS analysis.

Notably, in TD-GCMS measurements of 2D precursor phase-engineered α-$FAPbI_3$ we also detect trace quantities of BA^+ (Supplementary Fig. 28a). Given recent reports demonstrating that 2D-3D perovskite heterostructures can be employed to improve the stability of 3D perovskites[58,59] including α-$FAPbI_3$ it is important that we distinguish the mechanism of stability enhancements established here from previous works. First, we re-emphasise the exceptional

sensitivity of the TD-GCMS measurement for organic species. Assuming the same detection limit for BA^+ as for DMSO (0.002 wt%, as noted above) we calculate that in a 500 nm thick 25 cm^2 thin film just 100 ng of BA^+ would be sufficient for detection by TD-GCMS, corresponding to a FA^+:BA^+ stoichiometry on the order of 10^7:1. Thus, while TD-GCMS does confirm the presence of BA^+ in the material, it is likely the cation is present in only extremely small quantities. Given that BA^+ is not detected in our quantitative ^{1}H NMR measurements (Fig. 2c), the quantity of BA^+ must be below the detection limit of ^{1}H NMR (estimated at >10^3:1 FA^+:BA^+, considering clear resolution of ^{13}C satellite signals; Supplementary Fig. 11). We also note that in our TGA-MS measurements we observe no mass loss event corresponding to the removal of BA^+ (Supplementary Fig. 29), again suggesting the presence of close to zero BA^+ in the final material. Typically, previous works have shown that compositional contents of BA^+ ranging from 1 to 10% are required to increase the stability of 3D perovskite films[60]. Hence, at such dilute upper-limits of concentrations here, it is unlikely that trace BA^+ is playing a role in stabilising our 2D precursor phase-engineered α-$FAPbI_3$ material. However, to fully characterise the presence of BA^+ in our material, we first carry out GIWAXS measurements on 2D precursor phase-engineered α-$FAPbI_3$, which confirm the absence of any bulk 2D perovskite secondary phases (Supplementary Fig. 30). However, as such X-ray scattering techniques only allow for detection of bulk regions of crystalline phases, further verification is required. As noted above, in Supplementary Fig. 12-13 we present SED measurements suggesting that 2D perovskite phases are absent in our fully converted α-$FAPbI_3$ material. However, such electron microscopy-derived methods portray only a very small snapshot of the overall material, and thus may not offer a complete representation of the material. To avoid this limitation, techniques probing the effect of additive inclusion on the optoelectronic – or other – properties of the resulting material, which may be heavily affected by the presence or absence of trace compositional additives, can be utilised. Taking this approach, we conduct highly sensitive modulated surface photovoltage (SPV) spectroscopy measurements on our perovskite material at a series of times during conversion from 2D precursor phase to 3D $FAPbI_3$ (Supplementary Fig. 31). SPV signals are observed where photogenerated e^--h^+ pairs

are separated in space, and hence yield valuable information on the presence of any optoelectronically-active phases in the studied film. We observe a pronounced effect on the optoelectronic response of partially converted samples, corresponding to the presence of trace remaining 2D phases or 2D-3D perovskite heterostructures. All such features are entirely suppressed by completing the optimised conversion process, with the only above-bandgap optoelectronic signature corresponding to the presence of PbI_2 domains, consistent with our findings above (Fig. 3). Based on these findings, we determine that the enhanced thermal stability of 2D precursor phase-engineered α-$FAPbI_3$ is primarily a result of avoiding low volatility solvent retention and reducing the rate of FA^+ volatilisation (observed as *sym*-triazine evolution) by 2D-templating, rather than retention of BA^+ cations.

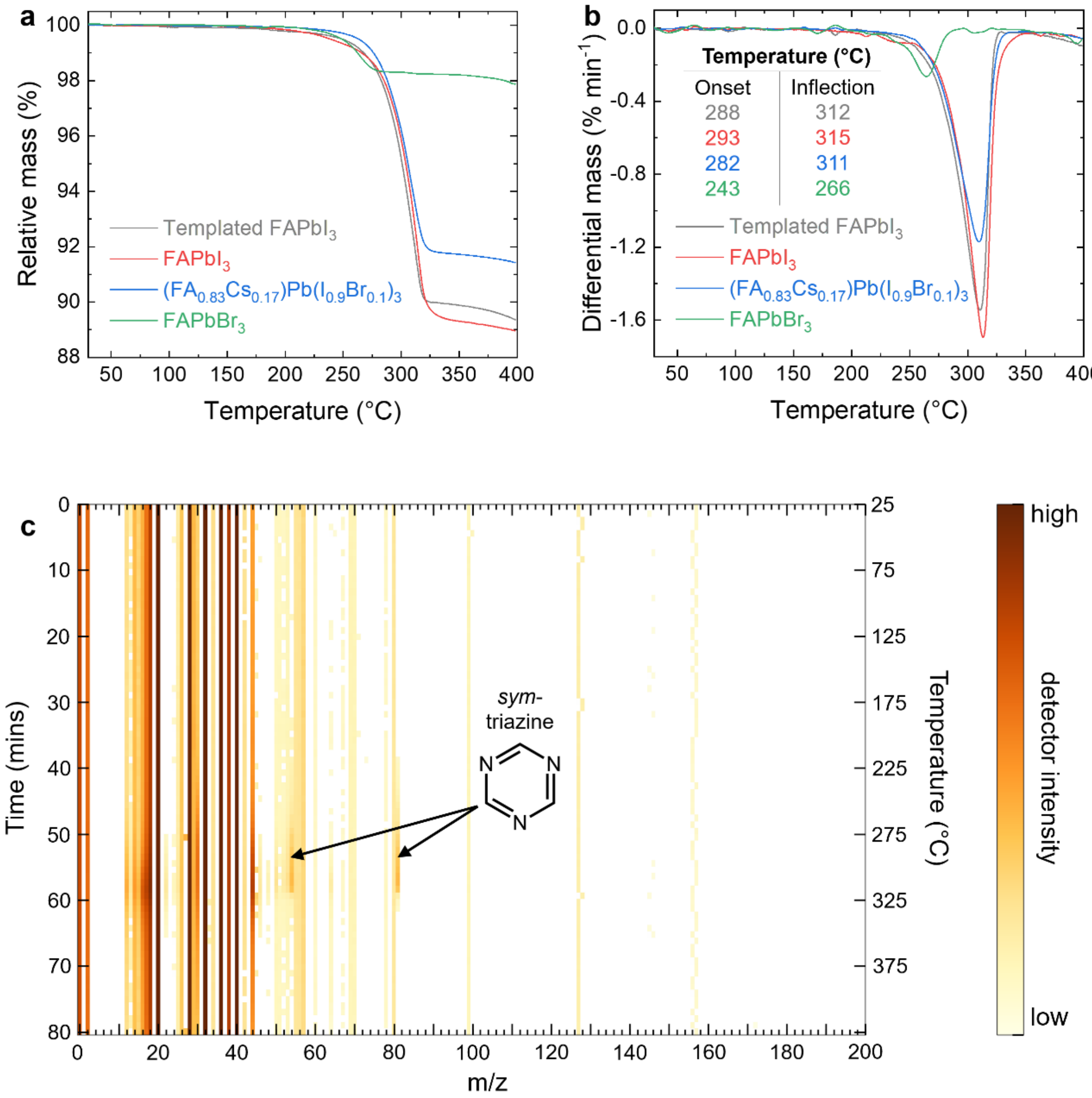

Supplementary Figure 29 | Investigating *sym*-triazine evolution. Thermal profiles (5 °C min^{-1}) of four perovskite materials during thermal gravimetric analysis-mass spectrometry (TGA-MS), with both relative mass (**a**) and differential mass (**b**) shown. Inset in **b** shows extracted onset and inflection temperatures of mass loss event corresponding to *sym*-triazine loss. (**c**) Corresponding time- (left) and temperature-resolved (right) mass spectrum for 2D-intermediate $FAPbI_3$ highlight onset of *sym*-triazine. Intensity detected at consistency throughout the measurement is attributed to species introduced by the N_2 carrier gas.

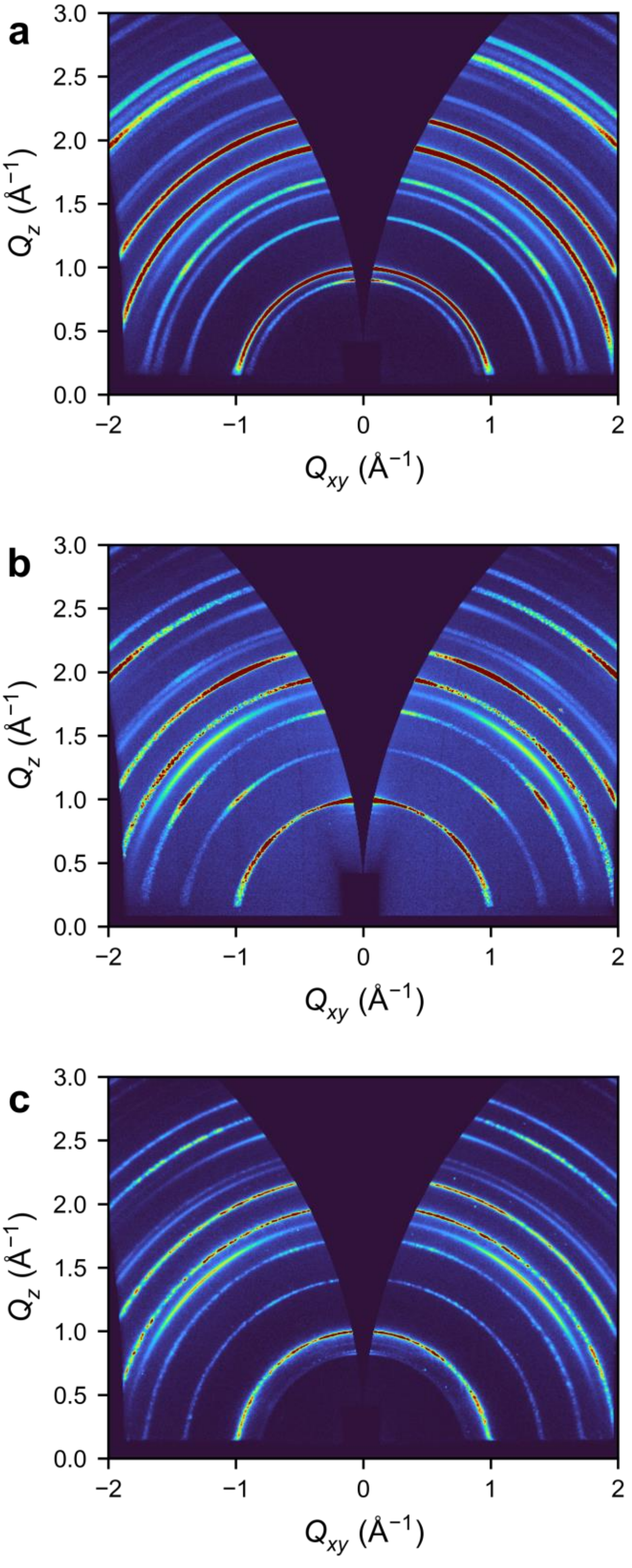

Supplementary Figure 30 | Grazing incidence wide-angle X-ray scattering (GIWAXS) of $FAPbI_3$ fabricated *via* different processes. GIWAXS patterns of 2D-intermediate $FAPbI_3$ (**a**), $FAPbI_3$-MACl (**b**) and $FAPbI_3$ (**c**).

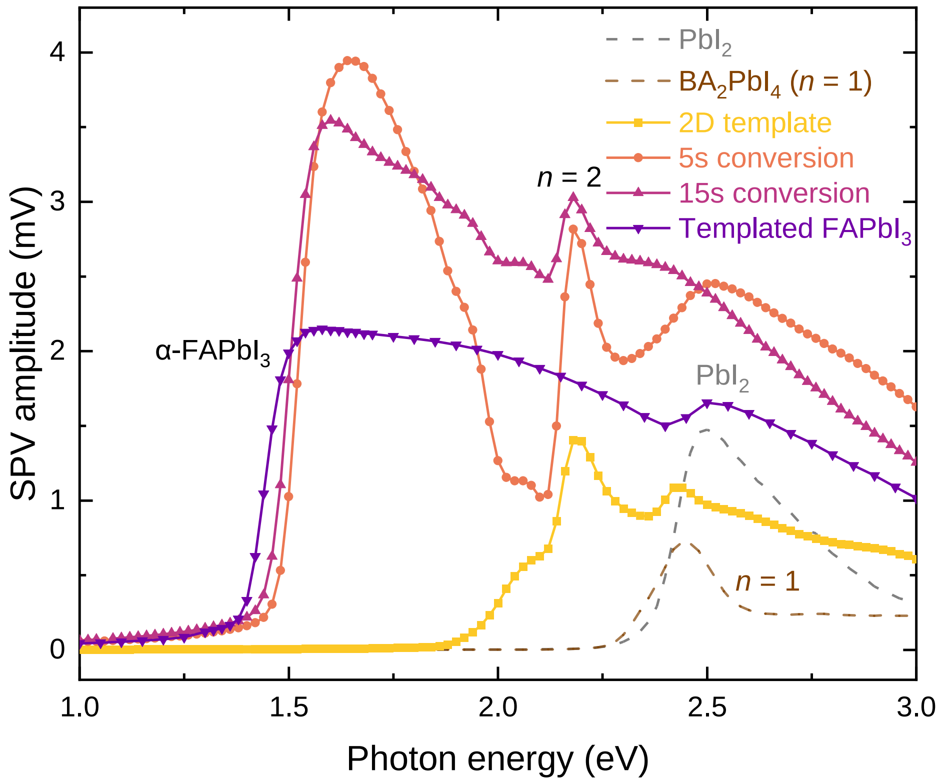

Supplementary Figure 31 | Optically-Modulated Surface Photovoltage (SPV) Measurements. SPV response of 2D intermediate, 2D-intermediate $FAPbI_3$, and partial conversion of the 2D intermediate by exposing intermediate to 5 or 15 seconds of soaking in the conversion solution before drying at 70 °C for 10 minutes. For reference, spectra corresponding to thin films of PbI_2 and BA_2PbI_4 (*n* = 1) are shown. No evidence of optoelectronic response corresponding to phases besides α-$FAPbI_3$ and PbI_2 observed.

Supplementary Note 8

Here we analyse the detail, and wider implications, of our TD-GCMS measurements.

1. *Sym*-triazine formation

As described by several authors *sym*-triazine[61,62], $C_3H_3N_3$ is expected to form in the perovskite materials *via*

$$FA^+ + X^- \rightarrow FA\ (g) + HX(g) \tag{17}$$

$$FA + 2FA^+ + 2X^- \rightarrow C_3H_3N_3(g) + NH_3(g) + 2NH_4X \tag{18}$$

where X^- is a halide ion.

2. Iodine observation and origin – A-site cation alloying

We note the presence of iodine (I_2) in many of the chromatograms presented. This has not been highlighted in the main text for two reasons. Firstly, there is no clear trend in iodine production with perovskite composition, processing or any other parameter. Secondly, the use of iodine vapour treatment within our laboratory is common and as such we highlight concerns that the iodine observed may be the result of lab-based trace contamination. For completeness, however, we discuss below the possible non-contaminant origins of I_2 evolving from thermally treated perovskite materials.

Iodine is a possible, although unusual, thermal degradation co-product of many halide perovskites. For example, A-site cation degradation in MA^+-containing perovskites. Under thermal conditions, this process is thought to typically proceed *via*

$$MA^+ + I^- \rightarrow MA\ (g) + HI(g) \tag{19}$$

yielding hydrogen iodide rather than I_2. However, hydrogen iodide is well known to decompose to iodine:

$$2HI \ \rightleftharpoons I_2 + H_2 \tag{20}$$

It is therefore possible that I_2 may be a direct thermal degradation product. This likelihood is increased in instances where rapid HI production is observed, leading to a significant concentration of HI and forcing the equilibrium described by Eq. 20 to rebalance, producing I_2.

Separately, I_2 may be introduced into the perovskite structure during processing either by radical ion processes – likely photocatalysed – or even as a common contaminant in ammonium halide precursors. Ammonium halides are typically synthesised using excess hydrohalic acids, which generate halogens (X_2) in-situ during the synthesis according to Eq. 20. Rigorous iterative recrystallization procedures should be employed to remove all X_2 present during purification, however this procedure is not always done to satisfaction, even by many commercial chemical producers.

I_2 present, and retained, in the as-formed perovskite may be released and observed during TD-GCMS analysis. However, its presence within the perovskite material is particularly troublesome as I_2 has been shown to catalyse the A-site cation degradation[63]:

$$I_2 + I^- \rightleftharpoons I_3^- \quad (21)$$

$$MA^+ + I_3^- \rightarrow MA + I_2 + HI \quad (22)$$

On the basis of this discussion, we highlight the critical need to minimise I_2 contamination to enable the fabrication of stable perovskite materials.

Returning to our TD-GCMS results, we tentatively highlight the correlation between A-site cation alloying and the absence of I_2 production. Significantly more study should be invested into confirming these results, however there is a strong correlation between $FAPbI_3$ and particularly $MAPbI_3$ perovskites fabricated *via* a range of techniques and the observation of I_2 evolution during thermal aging. Indeed, the gas chromatograms corresponding to $MAPbI_3$ fabricated *via* both ACN and DMF:DMSO are dominated by I_2 (Supplementary Fig. 28o-p). This may be indicative of the relatively facile deprotonation of MA^+ and rapid loss of methylamine (whose m/z is too small for convincing detection *via* TD-GCMS) and HI, as in Eq. 19, leading to a higher concentration of gaseous HI and production of significant I_2 *via* Eq. 20.

By contrast, FA_xCs_{1-x} A-site alloyed perovskites investigated almost universally show minimal or no I_2 evolution. There is some precedent for such a finding; using a hybrid experimental and calculations-based approach, Garcia-Fernandez, et al., demonstrated that mixed A-site perovskites – and in particular Cs-containing perovskites – are compositionally more stable than their non-alloyed counterparts[64]. We emphasise, however, that once again 2D-intermediate $FAPbI_3$ is a notable exception this rule, displaying no evidence of I_2 production as might be expected considering the processes described in Eq. 17-20 and that *sym*-triazine is also not observed (Supplementary Fig. 28a).

3. Wider implications of TD-GCMS results

Beyond the three key findings described in the main text, and that tentatively introduced above, we note a number of other significant consequences of our TD-GCMS measurements.

(i) In general, the presence of Cs^+ and Br^- tends to increase the likelihood of solvent retention (Supplementary Fig. 28b-n).

(ii) Processing in dry air conditions assists with solvent removal, although we speculate that this may be due to more to the significantly higher rate of gas flow employed during our dry air processing as compared to N_2 processing (Supplementary Fig. 28m-n).

(iii) MACl additive has limited effect on solvent retention (Supplementary Fig. 28j, n).

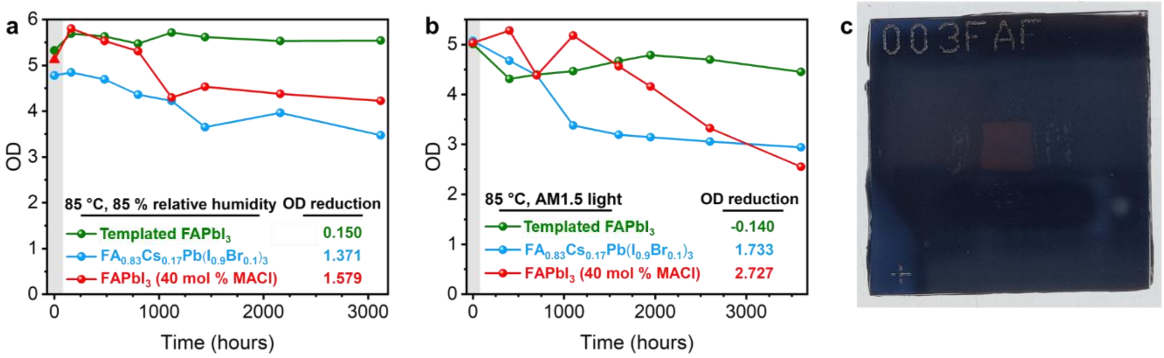

Supplementary Figure 32 | Operational material stability: Absorbance. Plots tracking change in optical density (OD) of encapsulated state-of-the-art perovskite thin films in region from 500-510 nm under 85 °C, 85% relative humidity (**a**) and 85°C, 1-sun equivalent illumination (**b**) conditions. Data points plotted within the shaded regions are based on smoothed absorbance spectra due to detector saturation. For this reason, OD reductions reported are for values outside the shaded region. **c**, Photograph of encapsulation of thin film materials using polyisobutene (PIB) edge sealant.

In Supplementary Fig. 32a-b, we show the optical density (OD) of the films, at wavelengths between 500 to 510 nm, as a function of stressing time. This gives a relatively sensitive indication of the evolution of "pin-holes" in the perovskite films, which have previously been observed to form during degradation[23,74]. A drop in OD from 5, to 4, to 3 corresponds to a pin-hole density increase from 0.001, to 0.01 to 0.1%, respectively. We therefore interpret the maintenance of high absorbance in aged 2D-intermediate α-$FAPbI_3$ – in contrast to two perovskite compositions that have previously been reported as amongst the most stable[23,75] – under both test scenarios to correspond to a low rate of pin-hole formation and hence degradation. We confirm this with visible light microscopy, where in Fig. 5i-l we show images of the different films after >3,000 hours stressing.

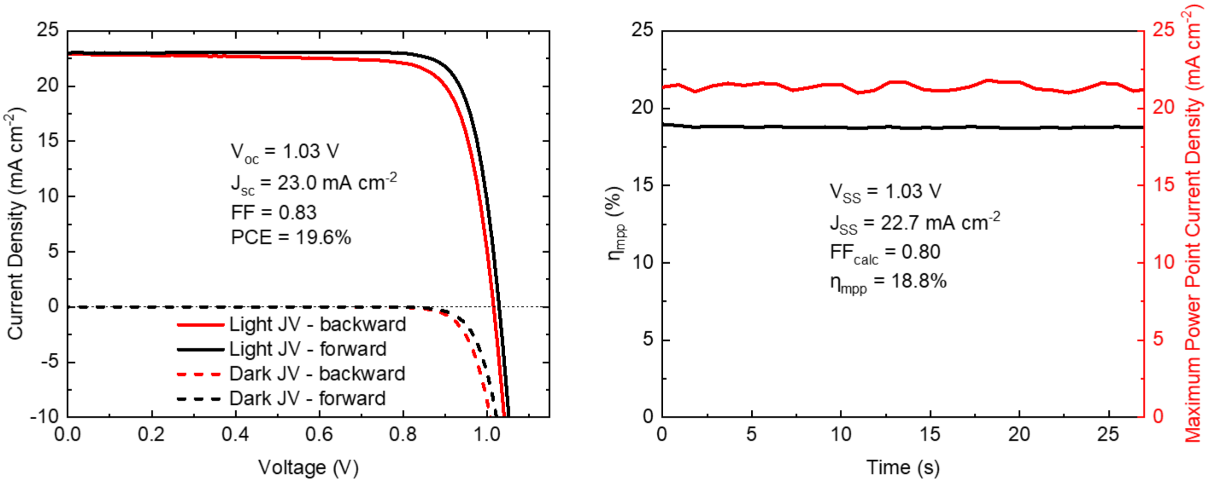

Supplementary Figure 33 | p-i-n PSC champion performance. J-V characteristics (**left**) and corresponding maximum power point tracked efficiency (η_{mpp}) and current density (J_{mpp}) measured at maximum power point (**right**) for champion devices fabricated from 2D-intermediate α-$FAPbI_3$ with illuminated area 1.00 cm^2. Light J-V and η_{mpp} measurements were performed under simulated AM1.5 100 $mWcm^{-2}$ irradiance. J_{sc} = short-circuit current density, V_{oc} = open-circuit voltage, FF = fill factor, V_{mpp} = maximum power point voltage. V_{SS} = stabilised V_{oc}, J_{SS} = stabilised J_{sc}, FF_{calc} = pseudo-FF calculated from independent stabilised voltage, current density and efficiency measurements.

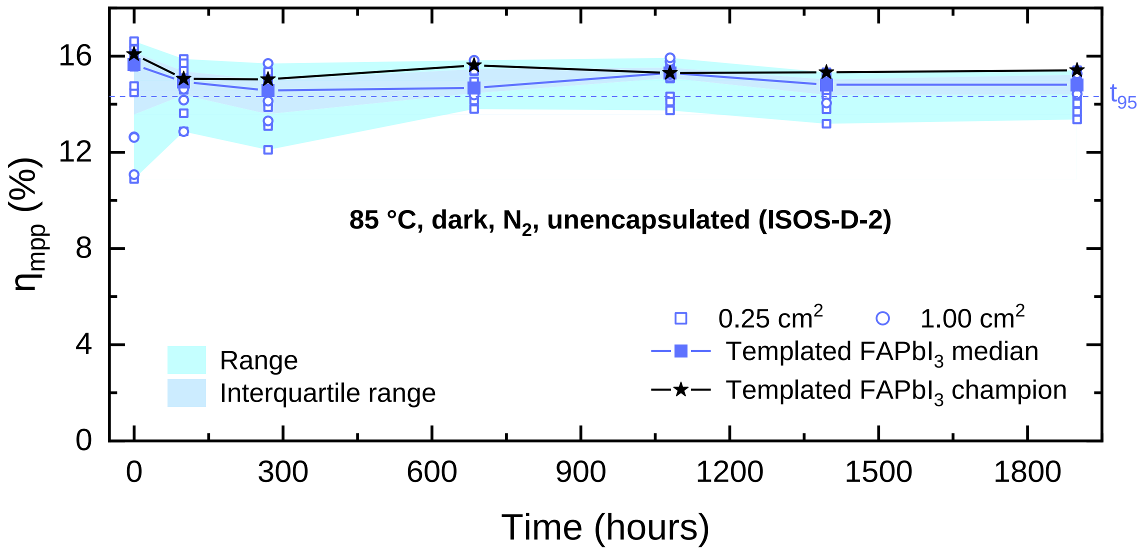

Supplementary Figure 34 | p-i-n PSC dark heat stability. Evolution of maximum power tracked efficiency (η_{mpp}) measured periodically on unencapsulated p-i-n PSCs (15 cells) employing 2D-intermediate $FAPbI_3$ as the photoactive layer, and thermally aged at 85 °C in the dark in an N_2 atmosphere (ISOS-D-2). Each measurement is taken after removing the device from the aging chamber and allowing it to cool to room temperature. The median, range and interquartile range of the dataset is shown, as well as each data point within it.

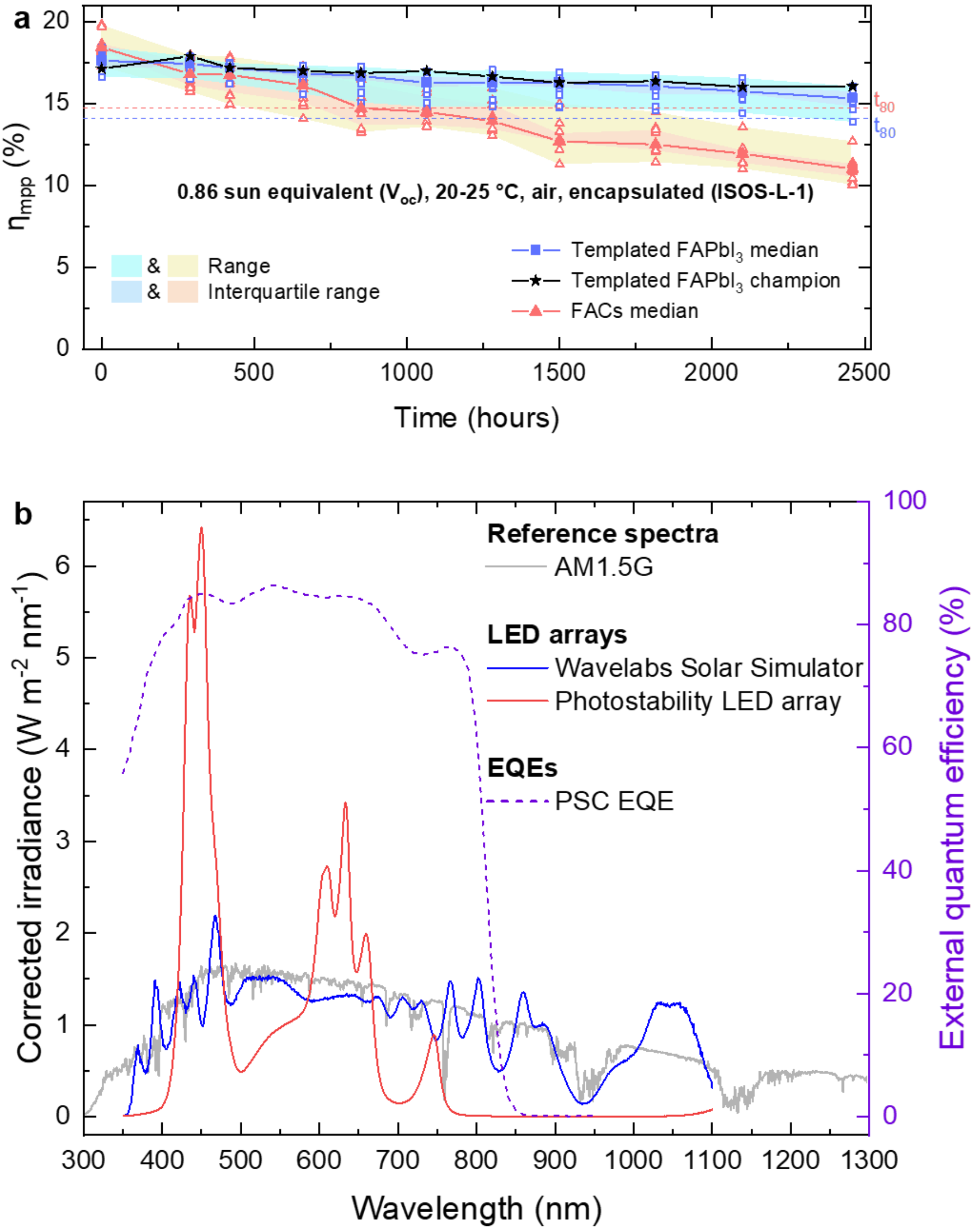

Supplementary Figure 35 | p-i-n PSC photostability. **a**, Evolution of maximum power tracked efficiency (η_{mpp}) measured periodically on p-i-n PSCs employing 2D-intermediate $FAPbI_3$ (21 cells) or $FA_{0.83}Cs_{0.17}Pb(I_{0.9}Br_{0.1})_3$ (10 cells, "FACs") as the photoactive layer, and encapsulated with an on-cell epoxy resin and cover slip. Cells actively cooled to between 20-25 °C and exposed to 0.86 sun equivalent light (LED array) (adapted ISOS-L-1). The range and interquartile range of each dataset is shown, as well as each data point within it. **b**, Relevant spectral irradiance for adapted ISOS-L-1 operational aging and measurement. AM1.5 spectrum is shown for reference alongside the Wavelabs solar simulator spectrum (see above for full description), used for measurement of all PSCs in this study. The LED array employed to age PSCs in our photostability (adapted ISOS-L-1) measurements (at V_{oc}) is also shown. Using the EQE shown (for a 2D-intermediate $FAPbI_3$ PSC) we calculate the sun equivalents of this setup to be 0.86 suns.

Supplementary Note 9

Our initial ISOS-L-2 (85 °C, 1 sun illumination) study showed rapid drop off in 2D-intermediate $FAPbI_3$ PSC η_{mpp}. However, this reduction in performance manifested as a reduction in the V_{oc} and fill factor of the aged PSCs, but not in the J_{sc}. As shown in Supplementary Fig. 36a, stabilised J_{sc} remains only falls from 22.8 to 20.7 mA cm^{-2} after 1,185 hours of ISOS-L-2 aging (median values, 10 cells). The retention of >90% of initial current density after over 1,000 hours of aging suggests that the perovskite photoabsorber has remained predominantly intact, and that the drop in performance observed is due to degradation elsewhere in the PSC (in other device layers, or at the interfaces between these).

Several reports have previously demonstrated that direct contact between epoxy resins used in on-cell encapsulation methods and the PSC metal electrodes leads to rapid degradation under operational aging[13,65]. As the stability of 2D-intermediate $FAPbI_3$ p-i-n PSCs under both adapted ISOS-L-1 conditions (ambient temperature, encapsulated with epoxy resin in direct contact with the metal electrode) and ISOS-D-2 (85 °C, dark, unencapsulated) is high, we speculated that this might be the leading cause of degradation in our PSCs. Previous reports have proposed evaporated metal oxide barrier layers (200-1,000 nm thick) on top of the metal electrode as a means to eliminate or reduce this degradation. Thus, we evaporated a 250 nm molybdenum oxide barrier layer on top of all PSCs used in further ISOS-L-2 aging experiments[13]. The only difference between 2D-intermediate $FAPbI_3$ p-i-n PSCs aged under ISOS-L-2 in Supplementary Fig. 36a and Fig. 5m is the presence of this barrier layer, reiterating that often it is not the bulk perovskite – nor even the interface between the perovskite photoabsorber layer and adjacent materials – that is the limiting cause in PSC degradation.

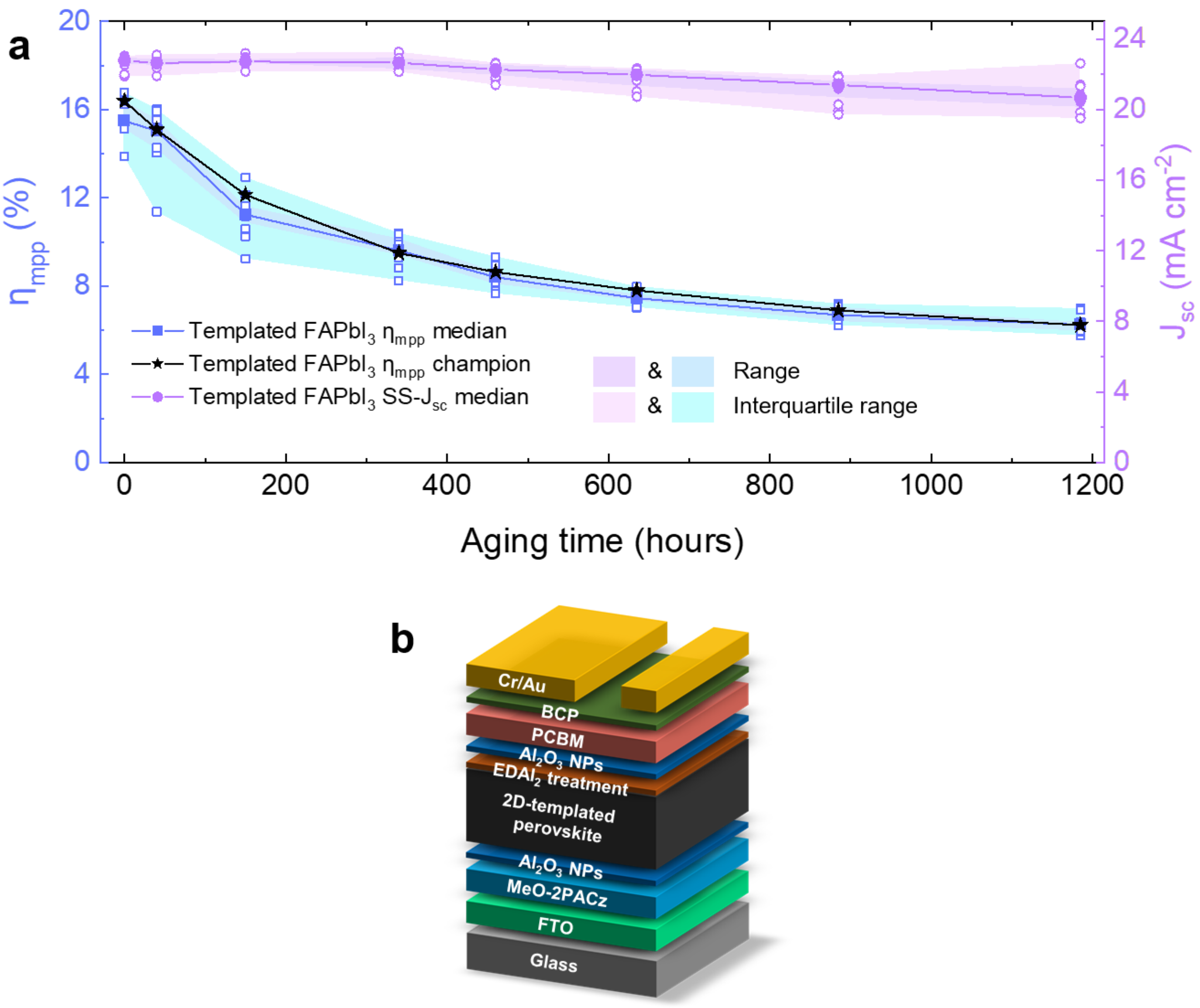

Supplementary Figure 36 | p-i-n PSC ISOS-L-2 aging without barrier layer. a, Evolution of maximum power tracked efficiency (η_{mpp}) measured periodically on p-i-n PSCs employing 2D-intermediate $FAPbI_3$, encapsulated with an on-cell epoxy resin and cover slip (without a MoO_3 barrier layer) and aged at 85 °C under 1 sun illumination (ISOS-L-2). Evolution of stabilised J_{sc} (SS-J_{sc}) also shown, highlighting that the photoabsorber remains intact and capable of charge separation and extraction, despite overall degradation in performance. The range and interquartile range of each dataset is shown, as well as each data point within it. **b**, Schematic representation of PSCs employed in this study.

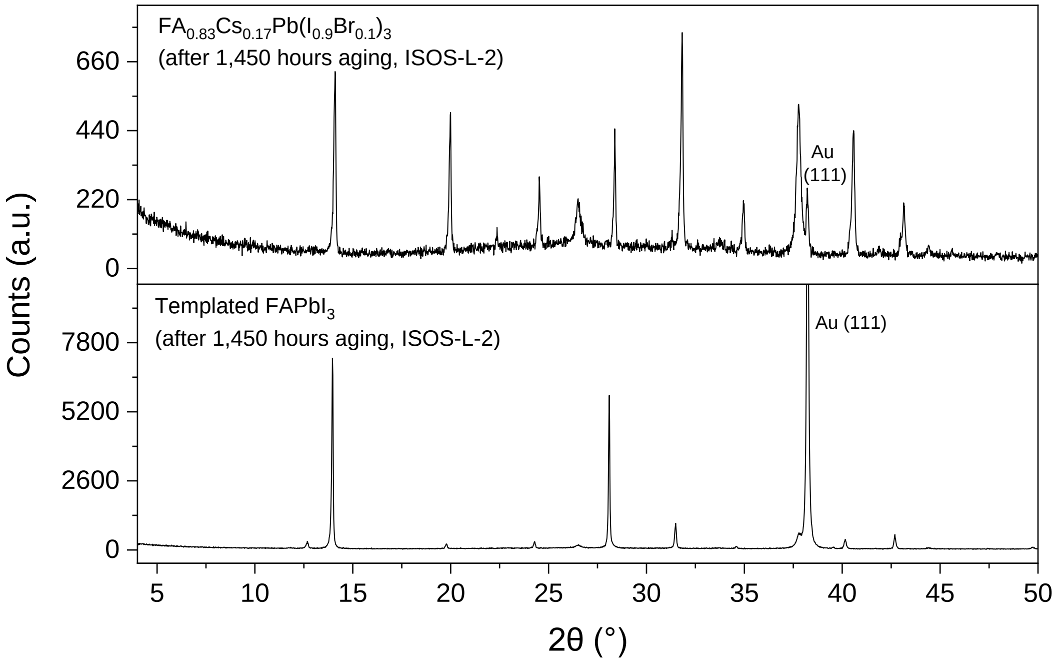

Supplementary Figure 37 | X-ray diffraction patterns of PSCs after 1,450 hours of ISOS-L-2 aging. Au is used as the metal electrode on these devices, and is incompletely removed during delamination and removal of the on-cell encapsulation during aging. Besides PbI_2, which is present in pristine 2D-intermediate $FAPbI_3$ no secondary phases are detected in either perovskite, attesting to the remarkably high compositional stability of both of these perovskites under ISOS-L-2 conditions.

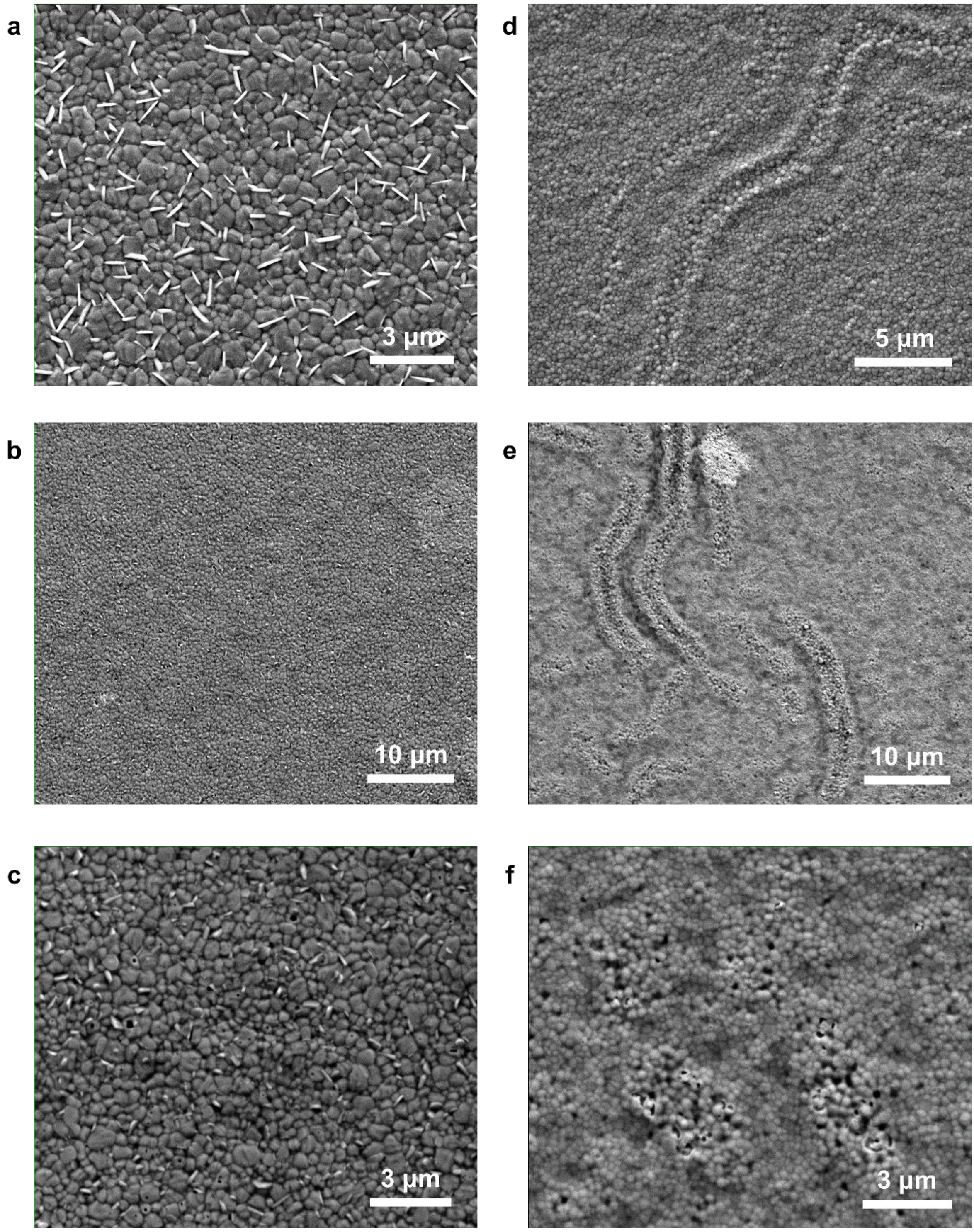

Supplementary Figure 38 | Scanning electron microscopy images of PSCs after 1,450 hours of ISOS-L-2 aging. 2D-intermediate $FAPbI_3$ without (**a**) and after 1,450 hours of ISOS-L-2 aging (**b-c**). $FA_{0.83}Cs_{0.17}Pb(I_{0.9}Br_{0.1})_3$ without (**d**) and after 1,450 hours of ISOS-L-2 aging (**e-f**). Images taken after aging are of the perovskite surface with remnant organic transport layers intact on top, which reduces the resolution of the images. All images of aged devices are taken outside of the region where Au metal electrode was deposited (i.e. outside of the active area), but where overlying MoO_3 and epoxy encapsulant have been mechanically delaminated. During aging the full device (not only the active area) is illuminated. "Wrinkles" observed in as-deposited $FA_{0.83}Cs_{0.17}Pb(I_{0.9}Br_{0.1})_3$ appear to act as regions in which pinhole formation is most severe.

In the $FA_{0.83}Cs_{0.17}Pb(I_{0.9}Br_{0.1})_3$ devices, void formation seems to occur preferentially in regions where “wrinkles” in the perovskite layer have formed during processing (Supplementary Figure S38d-f). Such wrinkles are often observed in mixed-halide, MA^+-free perovskites deposited via antisolvent-quenched DMF:DMSO methods and lead to local regions that are substantially thicker than their surroundings, and often of differing compositions[21,81]. We speculate that the increased thickness of the perovskite layer may lead to increased retention of low volatility solvents, consistent with the findings of Chen, et al.[14], with subsequent slow release during operation leading to void formation or accelerating degradation of the perovskite and causing material loss. Further investigation is required to confirm the link between wrinkle formation and pinhole formation; however, this observation reiterates the crucial relationship between processing route, solvent selection, and operational stability.

Supplementary Table 3 | PSC lifetimes under accelerated aging. Table summarising lifetimes reported in this work of PSCs undergoing aging by exposure to a variety of conditions. t_{95} and t_{80} values represent the number of hours taken for a η_{mpp} value to fall to 95% and 80% of its initial value, respectively. Values marked with * are projected t_{80} or t_{95} values extracted by linear fitting of all data points in the relevant dataset, and extrapolation. ">100% retained" is recorded where the final η_{mpp} recorded remains higher than the initial value (t_{100} > experiment duration). "η_{mpp} champion" refers the cell in each dataset with the highest absolute value of η_{mpp} at the end of experiment duration. These values typically, but do not necessarily, correspond to the longest t_{80} lifetime for any cell in that dataset. "% η_{mpp} retained" refers to the % of the initial η_{mpp} retained at the end of the duration of that test.

**Aging condition**	**Duration of test / hours**	**Lifetime metric**	**2D-intermediate α-$FAPbI_3$**		**$FA_{0.83}Cs_{0.17}Pb(I_{0.9}Br_{0.1})_3$**	
			**Median**	**η_{mpp} champion**	**Median**	**η_{mpp} champion**
**ISOS-D-2<br>85 °C, dark, N_2**	1,900	% η_{mpp} retained	94.6	95.8	-	-
**ISOS-L-1[#]<br>0.86 suns, LED, 20-25 °C, air**	2,460	% η_{mpp} retained	87.0	93.7	59.8	64.0
		t_{95} / hours	920	1,800	160	150
		t_{80} / hours	3,940*	5,440*	850	1,650
**ISOS-D-3<br>85 °C, 85% RH, air**	1,930	% η_{mpp} retained	109.9	116.6	80.9	96.8
		t_{95} / hours	>100% retained	>100% retained	315	2,590*
**ISOS-L-2<br>85 °C, AM1.5 equivalent, air**	965	% η_{mpp} retained	65.7	75.9	19.2	26.9
		t_{95} / hours	220	290	<10	20
		t_{80} / hours	570	800	40	100

ISOS-D-2: 85 °C oven, dark, unencapsulated, N_2 glovebox

ISOS-L-1: Cells actively cooled to room temperature and exposed to 0.86 suns equivalent of light from an LED array (spectrum shown in Supplementary Fig. 35b) and held at open circuit in air. Cells encapsulated with on-cell epoxy encapsulant (no MoO_3 barrier layer).

[#]Note: As this test is conducted at <1 sun equivalent illumination, it is best considered as under "adapted ISOS-L-1 conditions", as described throughout this work.

ISOS-D-3: Humidity chamber set to 85 °C and 85% relative humidity. Encapsulated with industry standard poly(isobutyl) edge sealant (see Supplementary Note 10 for full details).

ISOS-L-2: 85 °C, full spectrum (no UV filter) illumination and held at open circuit in air. Relative humidity inside the aging chamber not controlled. Cells encapsulated with on-cell epoxy encapsulant on top of a 250 nm MoO_3 barrier layer.

Supplementary Note 10

Encapsulation process (Supplementary Figure 39a):

1. Prepare p-i-n configuration PSCs as described above.
2. Attach solar ribbon wires 93M™ Charge-Collection Solar Tape 3007) to appropriate electrodes (see Supplementary Figure 39a).
3. Deposit transparent, UV-curable epoxy resin in centre of glass substrate (70 mm x 70 mm).
4. Place device with ribbons on top of epoxy resin, with ribbons overlaid on edge sealant tape, cleaning any glue from ribbon in regions where crossing edge sealant tape. UV-cure for 10 seconds.
5. Place interlocking strips of butyl rubber (50 mm x 10 mm) around each edge of bottom glass plate (underneath silver ribbons), ensuring < 1 mm gaps left between strips.
6. Add second layer of edge sealant strips on top of ribbon.
7. Add third layer of edge sealant strips, alternating the interlocking pattern.
8. Place top glass plate on top. Ensure that the top glass plate will not touch the device surface, even after vacuum sealing. Note: Glass spacers with thickness greater than the substrate can be epoxy-glued to the bottom glass plate to ensure this does not occur.
9. Vacuum seal and heat at 125 C under static vacuum for 15 minutes.

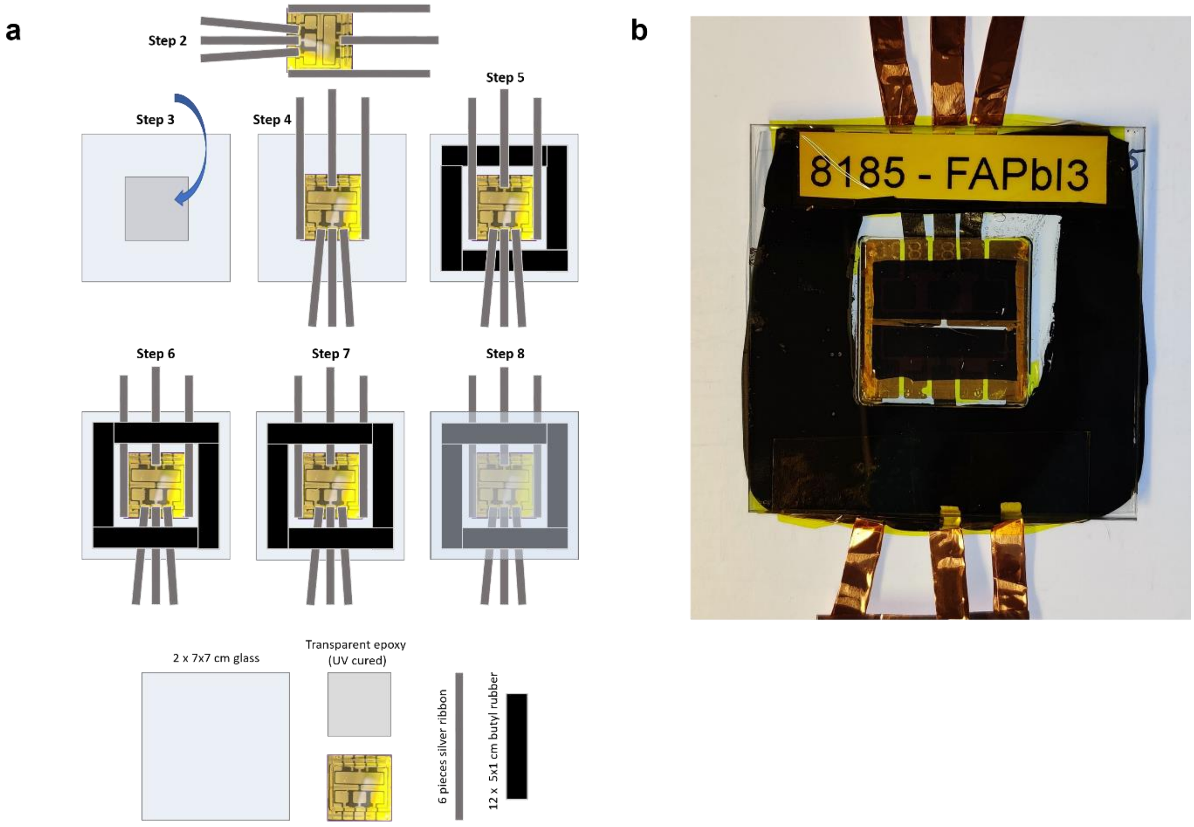

Supplementary Figure 39 | PSC encapsulation for damp heat (ISOS-D-3) testing. a, Schematic representation of encapsulation process for PSC encapsulation for damp heat stability testing. Steps correspond to those set out in Supplementary Note 10. b, Photograph of fully encapsulated p-i-n PSC ready for damp heat stability testing.

Supplementary References

1. Al-Ashouri, A. *et al.* Wettability Improvement of a Carbazole-Based Hole-Selective Monolayer for Reproducible Perovskite Solar Cells. *ACS Energy Lett* **8**, 898–900 (2023).

2. Jin, H. *et al.* Alumina Nanoparticle Interfacial Buffer Layer for Low-Bandgap Lead-Tin Perovskite Solar Cells. *Adv Funct Mater* **33**, 1–10 (2023).

3. Hu, S. *et al.* A Universal Surface Treatment for p-i-n Perovskite Solar Cells. *ACS Appl Mater Interfaces* **14**, 56290–56297 (2022).

4. Hu, S. *et al.* Optimized carrier extraction at interfaces for 23.6% efficient tin-lead perovskite solar cells. *Energy Environ Sci* 2096–2107 (2022) doi:10.1039/d2ee00288d.

5. Longo, G. *et al.* Understanding the Performance-Limiting Factors of Cs2AgBiBr6Double-Perovskite Solar Cells. *ACS Energy Lett* **5**, 2200–2207 (2020).

6. Savill, K. J. *et al.* Impact of Tin Fluoride Additive on the Properties of Mixed Tin-Lead Iodide Perovskite Semiconductors. *Adv Funct Mater* **30**, 1–13 (2020).

7. Yamada, Y., Nakamura, T., Endo, M., Wakamiya, A. & Kanemitsu, Y. Photocarrier recombination dynamics in perovskite CH3NH 3PbI3 for solar cell applications. *J Am Chem Soc* **136**, 11610–11613 (2014).

8. Yamada, Y., Endo, M., Wakamiya, A. & Kanemitsu, Y. Spontaneous defect annihilation in CH3NH3PbI3 thin films at room temperature revealed by time-resolved photoluminescence spectroscopy. *Journal of Physical Chemistry Letters* **6**, 482–486 (2015).

9. Joyce, H. J., Boland, J. L., Davies, C. L., Baig, S. A. & Johnston, M. B. A review of the electrical properties of semiconductor nanowires: Insights gained from terahertz conductivity spectroscopy. *Semiconductor Science and Technology* vol. 31 Preprint at https://doi.org/10.1088/0268-1242/31/10/103003 (2016).

10. Ashiotis, G. *et al.* The fast azimuthal integration Python library: PyFAI. *J Appl Crystallogr* **48**, 510–519 (2015).

11. Dane, T. G. The pygix library. https://github.com/tgdane/pygix.

12. Snaith, H. J. How should you measure your excitonic solar cells? *Energy Environ Sci* **5**, 6513–6520 (2012).

13. Belich, N. A. *et al.* How to stabilize standard perovskite solar cells to withstand operating conditions under an ambient environment for more than 1000 hours using simple and universal encapsulation. *Journal of Energy Chemistry* **78**, 246–252 (2023).

14. Stoumpos, C. C. *et al.* Ruddlesden-Popper Hybrid Lead Iodide Perovskite 2D Homologous Semiconductors. *Chemistry of Materials* (2016) doi:10.1021/acs.chemmater.6b00847.

15. Stoumpos, C. C. *et al.* Ruddlesden-Popper Hybrid Lead Iodide Perovskite 2D Homologous Semiconductors. *Chemistry of Materials* **28**, 2852–2867 (2016).

16. Guo, Y. *et al.* Chemical Pathways Connecting Lead(II) Iodide and Perovskite via Polymeric Plumbate(II) Fiber. *J Am Chem Soc* **137**, 15907–15914 (2015).

17. Yan, K. *et al.* Hybrid Halide Perovskite Solar Cell Precursors: Colloidal Chemistry and Coordination Engineering behind Device Processing for High Efficiency. *J Am Chem Soc* **137**, 4460–4468 (2015).

18. Hu, Q. *et al.* In situ dynamic observations of perovskite crystallisation and microstructure evolution intermediated from [PbI 6]4- cage nanoparticles. *Nat Commun* **8**, 1–9 (2017).

19. Cao, J. *et al.* Identifying the Molecular Structures of Intermediates for Optimizing the Fabrication of High-Quality Perovskite Films. *J Am Chem Soc* **138**, 9919–9926 (2016).

20. Chao, L. *et al.* Solvent Engineering of the Precursor Solution toward Large-Area Production of Perovskite Solar Cells. *Advanced Materials* **2005410**, 1–23 (2021).

21. Hamill, J. C., Schwartz, J. & Loo, Y. L. Influence of Solvent Coordination on Hybrid Organic-Inorganic Perovskite Formation. *ACS Energy Lett* **3**, 92–97 (2018).

22. Deng, Y. *et al.* Tailoring solvent coordination for high-speed, room-temperature blading of perovskite photovoltaic films. *Sci Adv* **5**, (2019).

23. Doolin, A. J. *et al.* Sustainable solvent selection for the manufacture of methylammonium lead triiodide (MAPbI 3) perovskite solar cells. *Green Chemistry* **23**, 2471–2486 (2021).

24. Prat, D. *et al.* CHEM21 selection guide of classical- and less classical-solvents. *Green Chemistry* **18**, 288–296 (2015).

25. Prat, D., Hayler, J. & Wells, A. A survey of solvent selection guides. *Green Chemistry* **16**, 4546–4551 (2014).

26. Zhang, M. *et al.* Green Anti-Solvent Processed Planar Perovskite Solar Cells with Efficiency Beyond 19%. *Solar RRL* (2018) doi:10.1002/solr.201700213.

27. Vidal, R. *et al.* Assessing health and environmental impacts of solvents for producing perovskite solar cells. *Nat Sustain* **4**, 277–285 (2021).

28. Wang, Z., Richter, S. M., Gates, B. D. & Grieme, T. A. Safety concerns in a pharmaceutical manufacturing process using dimethyl sulfoxide (DMSO) as a solvent. *Org Process Res Dev* **16**, 1994–2000 (2012).

29. Reichardt, C. *Solvents and Solvent Effects in Organic Chemistry*. (Wiley-VCH, 2002). doi:10.1002/3527601791.

30. Yaws, C. *Matheson Gas Data Book*. (McGraw-Hill, 2001). doi:1001/cat/bib/4480977.

31. International council for harmonisation of technical requirements for pharmaceuticals for human use. Guideline for Residual Solvents: Q3C(R8). *ICH* (2021).

32. International council for harmonisation of technical requirements for pharmaceuticals for human use. Guideline for Residual Solvents: Q3C(R8) - PDE for 2-methyltetrahydrofuran, cyclopentyl methyl ether, and tertiary-butyl alcohol. *ICH* **31**, (2020).

33. Mao, L., Stoumpos, C. C. & Kanatzidis, M. G. Two-Dimensional Hybrid Halide Perovskites: Principles and Promises. *J Am Chem Soc* **141**, 1171–1190 (2019).

34. Luo, D. *et al.* Enhanced photovoltage for inverted planar heterojunction perovskite solar cells. *Science (1979)* **360**, 1442–1446 (2018).

35. Yang, W. S. *et al.* High-performance photovoltaic perovskite layers fabricated through intramolecular exchange. *Science (1979)* **348**, 1234–1237 (2015).

36. Palosz, B. The structure of PbI2 polytypes 2H and 4H: A study of the 2H-4H transition. *Journal of Physics: Condensed Matter* **2**, 5285–5295 (1990).

37. Le Corre, V. M. *et al.* Revealing Charge Carrier Mobility and Defect Densities in Metal Halide Perovskites via Space-Charge-Limited Current Measurements. *ACS Energy Lett* **6**, 1087–1094 (2021).

38. Doherty, T. A. S. *et al.* Stabilized tilted-octahedra halide perovskites inhibit local formation of performance-limiting phases. *Science (1979)* **374**, 1598–1605 (2021).

39. Duijnstee, E. A. *et al.* Understanding the Degradation of Methylenediammonium and Its Role in Phase-Stabilizing Formamidinium Lead Triiodide. *J Am Chem Soc* **145**, 10275–10284 (2023).

40. Yamada, K. *et al.* Static and Dynamic Structures of Perovskite Halides ABX3 (B = Pb, Sn) and Their Characteristic Semiconducting Properties by a Hückel Analytical Calculation. *Bull Chem Soc Jpn* **91**, 1196–1204 (2018).

41. Zhang, Y. *et al.* From 2D to 3D: A facile and effective procedure for fabrication of planar CH3NH3PbI3 perovskite solar cells. *J Mater Chem A Mater* **6**, 17867–17873 (2018).

42. Miao, Y. *et al.* From 1D to 3D: Fabrication of CH 3 NH 3 PbI 3 Perovskite Solar Cell Thin Films from (Pyrrolidinium)PbI 3 via Organic Cation Exchange Approach. *Energy Technology* **2000148**, 1–5 (2020).

43. Adjokatse, S., Fang, H. H., Duim, H. & Loi, M. A. Scalable fabrication of high-quality crystalline and stable FAPbI3 thin films by combining doctor-blade coating and the cation exchange reaction. *Nanoscale* **11**, 5989–5997 (2019).

44. Conings, B. *et al.* Intrinsic Thermal Instability of Methylammonium Lead Trihalide Perovskite. *Adv Energy Mater* **5**, 1–8 (2015).

45. Turren-Cruz, S. H., Hagfeldt, A. & Saliba, M. Methylammonium-free, high-performance, and stable perovskite solar cells on a planar architecture. *Science (1979)* **362**, 449–453 (2018).

46. McMeekin, D. P. *et al.* A mixed-cation lead mixed-halide perovskite absorber for tandem solar cells. *Science (1979)* **351**, 151–155 (2016).

47. Schwenzer, J. A. *et al.* Thermal Stability and Cation Composition of Hybrid Organic-Inorganic Perovskites. *ACS Appl Mater Interfaces* **13**, 15292–15304 (2021).

48. Sun, Q. *et al.* Role of Microstructure in Oxygen Induced Photodegradation of Methylammonium Lead Triiodide Perovskite Films. *Adv Energy Mater* **7**, (2017).

49. Shin, S. *et al.* Enhancing Stability of Efficient Perovskite Solar Cells (PCE ≈ 24.5%) by Suppressing PbI2 Inclusion Formation. *Adv Funct Mater* **33**, 1–10 (2023).

50. Kim, M. *et al.* Methylammonium Chloride Induces Intermediate Phase Stabilization for Efficient Perovskite Solar Cells. *Joule* 1–14 (2019) doi:10.1016/j.joule.2019.06.014.

51. Gutierrez-partida, E. *et al.* Large-grain double cation perovskites with 18 μs lifetime and high luminescence yield for efficient inverted perovskite solar cells. *submitted to ACS Energy Letters* (2020) doi:10.1021/acsenergylett.0c02642.

52. McMeekin, D. P. *et al.* A mixed-cation lead mixed-halide perovskite absorber for tandem solar cells. *Science (1979)* (2016) doi:10.1126/science.aad5845.

53. Min, H. *et al.* Efficient, stable solar cells by using inherent bandgap of a-phase formamidinium lead iodide. *Science (1979)* **366**, 749–753 (2019).

54. Xu, J. *et al.* Triple-halide wide-band gap perovskites with suppressed phase segregation for efficient tandems. *Science (1979)* **367**, 1097–1104 (2020).

55. Saliba, M. *et al.* Cesium-containing triple cation perovskite solar cells: Improved stability, reproducibility and high efficiency. *Energy Environ Sci* **9**, 1989–1997 (2016).

56. Holzhey, P. *et al.* A chain is as strong as its weakest link – Stability study of MAPbI3 under light and temperature. *Materials Today* **29**, 10–19 (2019).

57. Noel, N. K. *et al.* A low viscosity, low boiling point, clean solvent system for the rapid crystallisation of highly specular perovskite films. *Energy Environ Sci* **10**, 145–152 (2017).

58. Jang, Y. W. *et al.* Intact 2D/3D halide junction perovskite solar cells via solid-phase in-plane growth. *Nat Energy* **6**, 63–71 (2021).

59. Azmi, R. *et al.* Damp heat – stable perovskite solar cells with tailored-dimensionality 2D / 3D heterojunctions. *Science (1979)* **376**, 73–77 (2022).

60. Wang, Z. *et al.* Efficient ambient-air-stable solar cells with 2D-3D heterostructured butylammonium-caesium-formamidinium lead halide perovskites. *Nat Energy* **2**, 1–10 (2017).

61. Juarez-Perez, E. J., Ono, L. K. & Qi, Y. Thermal degradation of formamidinium based lead halide perovskites into: Sym -triazine and hydrogen cyanide observed by coupled thermogravimetry-mass spectrometry analysis. *J Mater Chem A Mater* **7**, 16912–16919 (2019).

62. Schaefer, F. C., Hechenbleikner, I., Peters, G. A. & Wystrach, V. P. Synthesis of the sym-Triazine System. I. Trimerization and Cotrimerization of Amidines. *J Am Chem Soc* **81**, 1466–1470 (1959).

63. Wang, S., Jiang, Y., Juarez-Perez, E. J., Ono, L. K. & Qi, Y. Accelerated degradation of methylammonium lead iodide perovskites induced by exposure to iodine vapour. *Nat Energy* **2**, 1–8 (2017).

64. García-Fernández, A. *et al.* Benchmarking Chemical Stability of Arbitrarily Mixed 3D Hybrid Halide Perovskites for Solar Cell Applications. *Small Methods* **2**, 1800242 (2018).

65. Wong-Stringer, M. *et al.* High-Performance Multilayer Encapsulation for Perovskite Photovoltaics. *Adv Energy Mater* **8**, 1–11 (2018).
