# Supplementary material for: A green solvent enables precursor phase engineering of stable formamidinium lead triiodide perovskite solar cells
Source: Nat Commun. 2024 Nov 22;15:10110. doi: 10.1038/s41467-024-54113-4 (PMC11582696; doi:10.1038/s41467-024-54113-4)
Supplement: Supplementary file 3 — Reporting Summary [file 41467_2024_54113_MOESM3_ESM.pdf]

nature portfolio

Corresponding author(s): Prof. Dr Henry Snaith

Solar Cells Reporting Summary

Nature Portfolio wishes to improve the reproducibility of the work that we publish. This form is intended for publication with all accepted papers reporting the characterization of photovoltaic devices and provides structure for consistency and transparency in reporting. Some list items might not apply to an individual manuscript, but all fields must be completed for clarity.

For further information on Nature Research policies, including our data availability policy, see Authors & Referees.

Experimental design

Please check the following details are reported in the manuscript, and provide a brief description or explanation where applicable.

1. Dimensions

Area of the tested solar cells

- [x] Yes — 0.25 cm2 and 1.00 cm2
- [ ] No — *Explain why this information is not reported/not relevant.*

Method used to determine the device area

- [x] Yes — Shadow mask area, measured by visible light microscopy
- [ ] No — *Explain why this information is not reported/not relevant.*

2. Current-voltage characterization

Current density-voltage (J-V) plots in both forward and backward direction

- [x] Yes — Fig. 4; SI Fig. 14, SI Fig. 27
- [ ] No

Voltage scan conditions

- [x] Yes — Methods
- [ ] No — *Explain why this information is not reported/not relevant.*

Test environment

- [x] Yes — Methods
- [ ] No — *Explain why this information is not reported/not relevant.*

Protocol for preconditioning of the device before its characterization

- [x] Yes — Methods
- [ ] No — *Explain why this information is not reported/not relevant.*

Stability of the J-V characteristic

- [x] Yes — Fig. 4; SI Fig. 14, SI Fig. 27
- [ ] No — *Explain why this information is not reported/not relevant.*

3. Hysteresis or any other unusual behaviour

Description of the unusual behaviour observed during the characterization

- [ ] Yes — *Provide a description of hysteresis or any other unusual behaviour observed during the characterization.*
- [x] No — N/A

Related experimental data

- [ ] Yes — *Provide a description of the related experimental data.*
- [x] No — N/A

4. Efficiency

External quantum efficiency (EQE) or incident photons to current efficiency (IPCE)

- [x] Yes — Methods; SI Fig. 9
- [ ] No — *Explain why this information is not reported/not relevant.*

A comparison between the integrated response under the standard reference spectrum and the response measure under the simulator

- [x] Yes — SI Fig. 9
- [ ] No — *Explain why this information is not reported/not relevant.*

For tandem solar cells, the bias illumination and bias voltage used for each subcell

☐ Yes — *Provide a description of the measurement conditions.*
☒ No — N/A

5. Calibration

Light source and reference cell or sensor used for the characterization

☒ Yes — Methods
☐ No — *Explain why this information is not reported/not relevant.*

Confirmation that the reference cell was calibrated and certified

☒ Yes — Methods
☐ No — *Explain why this information is not reported/not relevant.*

Calculation of spectral mismatch between the reference cell and the devices under test

☒ Yes — Methods
☐ No — *Explain why this information is not reported/not relevant.*

6. Mask/aperture

Size of the mask/aperture used during testing

☒ Yes — 0.25 cm2 and 1.00 cm2
☐ No — *Explain why this information is not reported/not relevant.*

Variation of the measured short-circuit current density with the mask/aperture area

☒ Yes — Fig. 4 and Methods
☐ No — *Explain why this information is not reported/not relevant.*

7. Performance certification

Identity of the independent certification laboratory that confirmed the photovoltaic performance

☐ Yes — *Identify the independent certification laboratory.*
☒ No — Not certified

A copy of any certificate(s)

☐ Yes — *Certificate copies should be provided in the Supplementary information. Please state the supplementary item number.*
☒ No — Not certified

8. Statistics

Number of solar cells tested

☒ Yes — Main text and figure captions
☐ No — *Explain why this information is not reported/not relevant.*

Statistical analysis of the device performance

☒ Yes — Main text and figure captions
☐ No — *Explain why this information is not reported/not relevant.*

9. Long-term stability analysis

Type of analysis, bias conditions and environmental conditions

☒ Yes — Described in full in the main text and SI.
☐ No — *Explain why this information is not reported/not relevant.*
